# A cortical circuit for orchestrating oromanual food manipulation

**DOI:** 10.21203/rs.3.rs-2338599/v1

**Published:** 2025-05-20

**Authors:** Z. Josh Huang, Xu An, Yi Li, Katherine Matho, Hemanth Mohan, X. Hermione Xu, Ian Whishaw, Adam Kepecs

## Abstract

The seamless coordination of hands and mouth — whether in humans eating corn on the cob or mice extracting sunflower seeds — represents one of evolution's most sophisticated motor achievements. Whereas spinal and brainstem circuits implement basic forelimb and orofacial actions, whether there is a specialized cortical circuit that assembles these actions to enable skilled oromanual manipulation remains unclear. Here, we discover a cortical area and its cell-type-specific circuitry that govern oromanual food manipulation in mice. An optogenetic screen of cortical areas and projection neuron types identified a rostral forelimb-orofacial area (RFO), wherein activation of pyramidal tract (PT ^Fezf2^ ) and intratelencephalic (IT ^PlxnD1^ ) neurons induced concerted posture, forelimb and orofacial movements resembling eating. In a freely moving pasta-eating behavior, pharmacological RFO inactivation impaired the sitting posture, hand recruitment, and oromanual coordination in pasta eating. RFO PT ^Fezf2^ and IT ^PlxnD1^ activity was closely correlated with oromanual pasta manipulation and hand-assisted biting. Optogenetic inhibition revealed that PTs ^Fezf2^ regulate dexterous hand and mouth movements while ITs ^PlxnD1^ play a more prominent role in oromanual coordination. RFO forms the hub of an extensive network, with reciprocal connections to cortical forelimb and orofacial sensorimotor areas, as well as insular and visceral areas. Within this cortical network, RFO PTs ^Fezf2^ project unilaterally to multiple subcortical, brainstem and spinal areas associated with forelimb and orofacial control, while ITs ^PlxnD1^ project bilaterally to the entire network and the ventrolateral striatum, and can mediate concurrent forelimb and mouth movement in part through their striatal projection. Together, these findings uncover the cell-type-specific implementation of a cortical circuit that orchestrates oromanual manipulation, essential for skilled feeding.

Xu An[1,2], Katherine Matho[2], Yi Li[1,2], Hemanth Mohan[1,2], X. Hermione Xu[1,5], Ian Q. Whishaw[3], Adam Kepecs[4*], Z. Josh Huang[1,2,5*]

[1]Department of Neurobiology, Duke University Medical Center, Durham, NC 27710, USA
[2]Cold Spring Harbor Laboratory, Cold Spring Harbor, NY 11724, USA
[3]Department of Neuroscience, Canadian Centre for Behavioural Research, University of Lethbridge, Lethbridge, AB, T1K 3M4, Canada
[4]Department of Neuroscience and Department of Psychiatry, Washington University in St. Louis, St. Louis, MO, USA.
[5]Department of Biomedical Engineering, Duke University Pratt School of Engineering, Durham, NC 27708, USA

[*]corresponding authors: josh.huang@duke.edu; akepecs@wustl.edu

### ABSTRACT

Cooperative forelimb and mouth movements during eating contribute to diet selection among vertebrates including the oromanual manipulatory skills in rodents and primates. Whereas spinal and brainstem circuits implement forelimb and orofacial actions, whether there is a specialized cortical circuit that flexibly assembles these to achieve cross-body and oromanual coordination for skilled manipulation remains unclear. Here we discover a cortical region and its cell-type-specific circuitry that orchestrates body postures and oromanual coordination for food manipulation in mice. An optogenetic screen of cortical areas and projection neuron types identified a rostral forelimb-orofacial area (RFO), wherein activation of pyramidal tract ($PT^{Fezf2}$) and intratelencephalic ($IT^{PlxnD1}$) neurons induced concurrent posture, forelimb and orofacial eating-like movements. In a pasta-eating behavior, RFO $PT^{Fezf2}$ and $IT^{PlxnD1}$ activity were closely correlated with picking up the pasta, adopting a sitting posture, oromanual manipulation, and hand-assisted biting. RFO inactivation and inhibition of RFO $PTs^{Fezf2}$ and $ITs^{PlxnD1}$ impaired posture and oromanual coordination, leading to deficient pasta manipulation and biting. RFO is reciprocally connected to forelimb and orofacial sensorimotor areas as well as insular and visceral areas. Within this network, $ITs^{PlxnD1}$ project bilaterally to the entire network and the ventrolateral striatum and $PTs^{Fezf2}$ project to multiple subcortical areas associated with forelimb and orofacial control. These results suggest that $ITs^{PlxnD1}$ select and coordinate the feeding program involving multiple body parts and $PTs^{Fezf2}$ implement the fine details of movements. Our study reveals a neural circuit basis of hand-mouth coordination for object manipulation.

### INTRODUCTION

Using the hands to assist feeding is characteristic of many vertebrate orders and amongst Euarchontoglires such as rodents and primates, features a sitting posture associated with cooperative food handling by the hands and the mouth [1-3]. This characteristic of feeding is a behavioral innovation that has diversified dietary options, relaxing constrains imposed by environmental niches [1,3]. The adoption of a sitting posture releases forelimbs from body support and allows for flexible coordination of hand and mouth movements. These movements feature the manipulation of food by the hands so that it can be oriented for transfer into the mouth, the transfer of food from the mouth to the hands for acts such as holding while chewing, and the cooperation of the hands and mouth in food preparation acts such as biting [3-5]. The neural circuitry that contributes to the orchestration of these skilled movements across multiple body parts, especially the coordination of hand and mouth in manipulation, is almost entirely unknown. Nevertheless, it is likely that the elaboration of this neural circuitry contributes to evolution of the diversity of hand skills in serial order displayed by higher primates including humans [6,7].

In the hierarchically organized vertebrate motor control infrastructure [8-10], lower-level controllers in brainstem regions are capable of issuing commands that mediate diverse actions such as reach, grasp, lick, bite, and chew [11]. How these actions are flexibly coordinated to achieve food retrieval and food manipulation toward an integrated behavior such as feeding is largely unclear. Although major insight has been gained from studying relatively isolated and well-trained forelimb movements, such as reach and grasp in non-human primates [12-15] and rodents [16-20], more complex and flexible natural behaviors to achieve ethological goals [21,22] have rarely been examined. In particular, little attention has been directed toward understanding the integrated movements of hands and mouth with body posture required for the complex behavior of food manipulation. This is due in part to the involvement of multiple body parts making it challenging to study the underlying brain circuit mechanisms.

The present study uses the laboratory mouse, which displays sophisticated sensorimotor behaviors that enable feeding on a wide variety of otherwise non-accessible food items, such as shelled seeds and nutrient-rich body parts of captured insects, through oromanual manipulation [5,23-26]. Thus, the mouse represents a valuable experimental model for exploring the neural basis of manipulation and enables the application of the full suite of genetic tools for neural circuit analysis [27,28]. Here, combining a systematic optogenetic screen of projection neuron (PN) types and cortical areas with a quantitative analysis of a natural feeding behavior, cell-type resolution neural recording, functional manipulation, and input-output circuit mapping, we describe a cortical area and its associated brain circuits that orchestrate body postures and oromanual coordination for food manipulation.

### RESULTS

#### Optogenetic identification of a cortical area that elicits oromanual fictive eating

We performed an optogenetic activation screen to identify cortical regions involved in coordinated forelimb and orofacial movements. Classic micro-stimulation experiments in humans [29], non-human primates [30,31], and rodents [32-34] have revealed topographic motor maps of cortical areas that induce body part movement. Recent optogenetic activation studies in mice have probed more restricted cortical cell populations in motor control [35-37], but these have been limited to a few mostly mixed neuronal populations (e.g. Thy1 transgenic lines), and thus have yet to achieve neuron type and neural circuit resolution. We have recently generated a suite of mouse knock-in driver lines targeting hierarchically organized cortical PNs, including pyramidal tract (PT), corticothalamic (CT), and intratelencephalic (IT) classes, and subpopulations therein [38]. To systematically examine the role of different cortical areas and PN types in forelimb and orofacial motor control, we used these drive lines to express channelrhodopsin (ChR2) in 8 different neural populations, including subpopulations of PT (*Fezf2, Tcerg1l, Sema3e*), IT (*PlxnD1, Tbr2*), and CT (*Tle4*) neurons, with comparisons to a broad PN line (*Emx1*) and a previously used *Thy1* transgenic line 18 (*Thy1-Tg18*) targeting mixed PN populations [39] (**Fig. 1a**). Using a head-fixed preparation, we directed a laser beam (473 nm, 50 Hz, 0.5 s) through thinned skull to activate each of 128 sites on a 375-µm resolution grid within a 3 mm x 6 mm region of the right dorsal cortex while recording forelimb and orofacial movements using high-speed cameras (**Fig. 1b, c**). Among the 8 driver lines screened (**Fig. 1d, Extended Data Fig. 1a-f**), $PT^{Fezf2}$ and $IT^{PlxnD1}$ activation induced robust and coordinated forelimb and orofacial movements; we thus focused subsequent investigation on these two cell types.

The *Fezf2-CreER* driver line captures a majority of corticofugal neurons projecting to striatal, thalamic, collicular, brainstem, and spinal targets [38]. Activation of $PTs^{Fezf2}$ across the dorsal cortex of *Fezf2;Ai32* mice (expressing ChR2 in $PTs^{Fezf2}$) revealed a topographic motor map of contralateral forelimb and orofacial movements organized along a postero-medial to antero-lateral axis (**Fig. 1d, Extended Data Fig. 2a, b, g, i, j**). Posterior caudal forelimb area (pCFA) stimulation induced lateral forelimb abduction with elbow extension as well as digit opening and extension (**Fig. 1d, Extended Data Fig. 2d, g, Supplementary Video 1**). Medial caudal forelimb area (mCFA) stimulation evoked rhythmic forelimb treading (up-down) movements (**Extended Data Fig. 2a-c, e, Supplementary Video 2**). Anterior caudal forelimb area (aCFA) stimulation induced stepping or reaching-like forelimb movements involving sequential elbow, wrist, and digit flexion followed by extension (**Fig. 1d, Extended Data Fig. 2f, g, Supplementary Video 3**). Notably, $PT^{Fezf2}$ activation in an area anterolateral to the CFA induced robust and concurrent forelimb-orofacial movements, which included contralateral forelimb adduction to the body midline with hand supination and digit flexing and closing, jaw opening, and tongue protrusion (**Fig. 1d-j, Extended Data Fig. 2g, Supplementary Video 4**). The sequence of the forelimb and jaw movements appeared to reflect a coordinated behavior suitable for delivering food to the mouth (**Fig. 1e**). We named this area the Rostral Forelimb Orofacial area (RFO). RFO lies at partially overlapped location with the tongue-jaw motor cortex (tjM1), previously identified by examining only orofacial movements [40].

The *PlxnD1-CreER* driver line captures a major IT population in L2/3/5A that projects bilaterally to the cortex and striatum [38]. $IT^{PlxnD1}$ activation in most cortical areas only induced weak or no observable forelimb movement (**Fig. 1d, Extended Data Fig. 2a, b, h**). Strikingly, $IT^{PlxnD1}$ activation in the RFO generated highly coordinated bilateral forelimb-orofacial movements that resembled eating (**Fig. 1d-j,**

**Extended Data Fig. 2h, Supplementary Video 5**). These movements included jaw opening with concurrent bilateral (5/13 mice) or unilateral (8/13 mice) hand-to-mouth withdraw, flexing and closing of the digits of both hands (**Fig. 1e-g, j**). The bilateral forelimb movements may be attributable to the bilateral projections of ITs$^{PlxnD1}$ to the cortex and striatum [38]. At the end of RFO IT$^{PlxnD1}$ and PT$^{Fezf2}$ activation, the contralateral hand was invariably moved to a consistent position close to the mouth regardless of its start positions (**Fig. 1f, h, i**), suggesting that the induced hand movement is mouth directed. IT$^{PlxnD1}$ and PT$^{Fezf2}$ activation in a more lateral part of the RFO induced rhythmic jaw movements along with hand-to-mouth withdraw (**Fig. 1d, i, Extended Data Fig. 2g-l**). The forelimb and orofacial movements induced by PT$^{Fezf2}$ and IT$^{PlxnD1}$ activation were robust to different stimulation frequencies and were induced primarily by long-duration stimulation (500 ms), whereas short-duration stimulation (100 ms) only induced brief restricted movements (**Fig. 1d, Extended Data Fig. 1g-k**).

Because optogenetic stimulation of RFO in *Fezf2;Ai32* and *PlxnD1;Ai32* mice could also activate axons of passage of ChR2-expressing PNs from other areas, we repeated these experiments using a viral strategy to express ChR2 specifically in RFO PTs$^{Fezf2}$ or ITs$^{PlxnD1}$ (**Extended Data Fig. 3a**). Activating RFO PTs$^{Fezf2}$ or ITs$^{PlxnD1}$ was sufficient to induce synergistic forelimb and orofacial movements similar to those observed in *Fezf2;Ai32* and *PlxnD1;Ai32* mice (**Extended Data Fig. 3b-i**). Thus, our results reveal a specific cortical area (RFO, **Fig. 1k**), where the activation of PT or IT PNs induce forelimb-orofacial movements that resemble natural eating behavior.

Among the 6 other driver lines we screened, PN$^{Emx1}$ activation induced forelimb and orofacial movements in the most wide-spread cortical areas (**Extended Data Fig. 1a, e, f**). The PN$^{Thy1\text{-}Tg18}$ forelimb motor map was diffuse and less topographically organized compared to that of PT$^{Fezf2}$ (**Fig. 1d, Extended Data Fig. 1b, e**). Activation of L2/3 ITs$^{Tbr2\text{-}E17}$ produced motor maps similar to those of ITs$^{PlxnD1}$, but the movements were weaker (**Fig. 1d, Extended Data Fig. 1c, e, f**). CT$^{Tle4}$ activation induced forelimb and orofacial movements mostly in the lateral areas relative to Bregma (**Extended Data Figs. 1d-f**). Neither PTs$^{Tcerg1l}$ nor PTs$^{Sema3E}$ induced significant movements (**Extended Data Fig. 1e, f**).

### RFO IT$^{PlxnD1}$ activation induces fictive eating with coordinated body and oromanual movements

To further explore the role of RFO in coordinating whole body movements associated with eating, we stimulated RFO PTs$^{Fezf2}$ and ITs$^{PlxnD1}$ in freely-moving mice. PT$^{Fezf2}$ activation induced a shoulder adduction that raised the contralateral hand toward the body midline, with associated hand supination and digit flexion. In addition, a concurrent ipsiversive head turning and lowering brought the snout to contact the radial surface of the left hand, while the ipsilateral hand maintained body support (**Fig. 1l-o, Supplementary Video 6**). Activation of RFO ITs$^{PlxnD1}$ induced a sitting posture and concomitant bilateral shoulder adduction that brought both hands to the body midline. During the adduction, the digits flexed and closed and contacted the mouth (**Fig. 1l-o, Supplementary Video 7**). These results reveal that RFO PNs, ITs$^{PlxnD1}$ in particular, mediate whole body movements for eating as well as the head, mouth, forelimb, hand, and digit movements of eating. Compared with head-fixed stimulation, RFO PT$^{Fezf2}$ and IT$^{PlxnD1}$ stimulation in free-moving mice had a lower probability of inducing hand-to-mouth movement but a high probability of inducing head-to-hand movements (**Fig. 1j, p, q**). Together, these results indicate that RFO-induced movements bring together the hand and the mouth (i.e., instead of bringing the hand to the mouth as in head-fixed mice) and this goal can be achieved in different ways according to behavioral context.

**Pasta eating requires oromanual dexterity and coordination in food handling**

To explore the role of RFO in food handling and eating, we established a behavioral task, a "Mouse Restaurant", in which mice retrieve and consume food items (**Fig. 2a, Extended Data Fig. 4a-d, Supplementary Video 8**). This setup featured automated, self-initiated trials (including automated food item delivery) that enable efficient testing of animals with minimal experimenter involvement (**Extended Data Fig. 4a-c**). Behavior was filmed by 3-synchronized video cameras, together with concurrent sound recording that allowed registering the biting events associated with oromanual movements (**Fig. 2a, Extended Data Fig. 4a, e**). In the task, mice were able to manipulate and eat different kinds of food items (pellets, angel-hair pasta, sunflower seeds, oats, etc.) largely without training (**Supplementary Videos 9, 10**) and learned to shuttle between the waiting and dining areas within 1-3 sessions (see Methods). Amongst food items tested, the angel-hair pasta presented several advantages. It has a consistent shape and length (15 mm), when bitten the sound is audible, and pasta-eating behavior has been previously characterized [41-44]. The Mouse Restaurant provided recordings of thousands of trials and millions of video frames of pasta-eating behavior. Using DeepLabCut [45], we labeled 12,623 images to track 10 body parts of the eating mice and three parts of the pasta. These included the left and right eyes, hands, ankles, nose, tongue, jaw, and tail base and the top, center, and bottom of the pasta (**Fig. 2b, Supplementary Video 11**). We analyzed over 4 million video frames to identify and annotate re-used units of movement, the action motifs [21], and sensorimotor events (**Extended data Fig. 5**). We then designed an actogram, which presents overlays of the location and action of key body parts and sensorimotor events, and co-registered biting events across an entire trial in a single graph (**Fig. 2c**).

The angel-hair pasta eating behavior was organized into several stages, each comprising multiple characteristic action motifs involving multiple body parts (**Fig. 2c, d**). Upon entering the dining area, mice *approach* the pasta and most often *retrieve* it from the floor by licking and then grasping it with the teeth (**Fig. 2c, d, Extended Data Figs. 4d, 5a, b**). They then immediately adopt a *sitting posture* on the haunches and subsequently transfer the pasta to their hands with both hands reaching for it (**Fig. 2c, d, Extended Data Fig. 5c, d**). The mice consume the pasta in repeated *handle-eat* bouts (**Fig. 2c, d**). After a piece of pasta is eaten, the mice *leave* the dining area.

The *handle-eat* bout was characterized by highly coordinated and dexterous manipulatory movements, with continual oromanual movements to appropriately position the pasta for eating. Each bout started with a hand withdraw that brought pasta to the opening mouth (**Fig. 2c, d, Extended Data Fig. 5e**). Hand movements resulted in a mouse using specialized grasp movements with each hand. One hand made a guide grasp, which held proximal end of the pasta in the mouth, most likely by pressing the pasta with the thumb. The other hand made a support grasp, in which the tips of the digits held the pasta more distally from the mouth and directed the pasta further into the mouth after each bite [43] (**Fig. 2b**). To advance the pasta into the mouth as it was reduced by biting, mice made frequent release and re-grasp movements with one or both hands to reposition the hand on the pasta (**Fig. 2c, d, Extended Data Fig. 5f, g**). These hand adjustments most often occurred just before the first bite of each bout in order to position the pasta between the teeth for the bite (**Fig. 2c, e, Extended Data Fig. 6a**). Frame-by-frame analysis further revealed that mice tended to make hand adjustments with pasta clenched by the mouth (**Fig. 2d, f, Extended Data Figs. 5f, g, 6b**), suggesting cooperative oromanual movements for pasta positioning, usually with a characteristic oblique angle between the hands and the teeth (**Fig. 2d**).

Analysis of hand movements shortly before and after biting revealed a rapid downward movement of both hands before a bite, suggesting that the hands exert a fulcrum-like action on the pasta to aid the bite (**Fig. 2g, h, Extended Data Fig. 6c-e**). A movement phase analysis uncovered that pasta biting was achieved by a cooperative hand and jaw action that *snapped* the pasta, producing an audible snapping sound (**Fig. 2i-l**). Pasta that was snapped from the stem was then chewed (**Fig. 2d**). Thus, pasta insertion into the mouth, positioning after insertion, and biting all involved coordinated movements between both the hands and the mouth (**Fig. 2d**). The various movements of eating can be described as action motifs (pick-up, sit and transfer to hands, withdraw toward the mouth, handle and bite, chew) because although varying from pasta to pasta and for each pasta as it is reduced in length with each bite, they are always recognizable and measurable.

**RFO is necessary for hand recruitment and oromanual manipulation in pasta eating**

To determine whether RFO was involved in pasta eating, we suppressed neural activity by bilateral infusion of $GABA_A$ receptor agonist, muscimol (**Extended Data Fig. 7a, b**). Following infusion, the mice were able to approach and locate the pasta in a seemingly normal way, but they showed deficits in grasping the pasta by licking (**Extended Data Fig. 7c-f**). For mice that managed to grasp the pasta by mouth and adopted a sitting posture, their hand recruitment was severely impaired. They usually failed to manipulate the pasta into a proper orientation for mouth grasping and biting. In attempting to eat, they displayed a hunched posture related to their difficulty with oromanual movements, and frequently dropped the pasta during consumption (**Fig. 2m-o, Extended Data Fig. 7c, d, Supplementary Video 12**). One mouse didn't adopt a sitting posture and consumed all of the pasta from the floor using only its mouth (**Extended Data Fig. 7g, Supplementary Video 13**). These impairments resulted in mice taking significantly longer to eat (**Fig. 2m, Extended Data Fig. 7c, d**), losing the pasta (e.g. pasta was thrown out of the dining area due to clumsiness of oromaunal movements), or leaving the dining area without finishing a piece of pasta. On the other hand, there were no deficits in hand grip force and bite force (**Extended Data Fig. 7h**). Together, these results indicate that RFO contributes to multiple movement modules from sitting posture to hand recruitment and oromanual coordination that are together required for coordinated eating behavior.

**RFO neural activity correlate with oromanual pasta manipulation**

To examine neural activity patterns within the RFO during pasta eating in freely-moving mice, we used fiber photometry to record population calcium dynamics from $PTs^{Fezf2}$ or $ITs^{PlxnD1}$ in the right RFO and, as a comparison, the left aCFA - an area involved in forelimb movement (**Fig. 3a-d, Supplementary Videos 14, 15**). $PT^{Fezf2}$ and $IT^{PlxnD1}$ activity patterns were broadly similar, we thus refer to their activity together as $PN^{Fezf2/PlxnD1}$ (**Fig. 3c, d**). As mice entered the dining area (marked by stepping across an elevated bar, **Fig. 2a**) to approach the pasta, $PN^{Fezf2/PlxnD1}$ activity in aCFA was higher than that in RFO, suggesting a role of aCFA in locomotion (**Extended Data Fig. 8**). Immediately following retrieval, as mice took a sitting posture and transferred the pasta from the mouth to the hands, RFO PN$^{Fezf2/PlxnD1}$ activity sharply increased and then fell and rose in proportion to food handling vigor (**Fig. 3c-f, h, i**). During the same period, aCFA PN$^{Fezf2/PlxnD1}$ activity decreased to baseline levels (i.e., levels when mice were resting in the waiting area; **Fig. 3c, d, f, i**). After the pasta was consumed and as a mouse left the dining area, RFO activity dropped whereas aCFA activity increased (**Fig. 3c, d**).

We next analyzed RFO PT$^{Fezf2}$ and IT$^{PlxnD1}$ activity patterns during the handle-bite periods and the chewing periods that were automatically identified by using a hidden Markov model (**Extended Data Fig. 9**). We found that elevated RFO PT$^{Fezf2}$ and IT$^{PlxnD1}$ activity was specifically correlated with handle-bite periods (**Fig. 3e, g, h, j**). The RFO activity increase was best correlated with the pasta positioning movement of getting pasta into the mouth but was not related to the movement of removing the pasta from the mouth after a bite (**Fig. 3e, g, h, j)**. Both PT$^{Fezf2}$ and IT$^{PlxnD1}$ activity rose after the onset of hand withdraw, with a shorter delay for ITs$^{PlxnD1}$ compared to that for PTs$^{Fezf2}$ (**Fig. 3g, j, k**), indicating that IT$^{PlxnD1}$ activity leads PT$^{Fezf2}$ activity in each handle-eat bout. In addition, a cross-correlation analysis revealed that the elevation of RFO activity reliably followed hand withdraw, measured as decreasing hand-to-nose distance (**Fig. 3l, m**), suggesting that PN$^{Fezf2/PlxnD1}$ activity was associated with controlling oromanual movements during the handle-bite period. Importantly, the correlation coefficient of IT$^{PlxnD1}$ activity was significantly higher than that of PT$^{Fezf2}$ activity (**Fig. 3m**), suggesting that ITs$^{PlxnD1}$ may compose an overarching sensorimotor program of oromanual manipulation whereas PTs$^{Fezf2}$ may broadcast commands for the execution of specific actions. RFO activity increase was also correlated with hand adjustments that advanced the pasta for a bite and with pasta biting/snapping (**Fig. 3e, g, h, j**). Activity declined sharply during chewing (**Fig. 3e, g, h, j**). Together, these results indicate that RFO PT$^{Fezf2}$ and IT$^{PlxnD1}$ activity are associated with the oromanual movements of positioning the pasta in the mouth and of biting it, and IT$^{PlxnD1}$ activity likely initiates the coordinated oromanual movements for food handling.

To further clarify whether RFO PN$^{Fezf2/PlxnD1}$ activity were associated with oromanual coordination or with eating using mouth only, we fed mice 1-mm long pieces of angel-hair pasta, which were eaten without sitting up and handling (**Fig. 3n, q, Supplementary Videos 16, 17**). RFO PT$^{Fezf2}$ and IT$^{PlxnD1}$ activity rose immediately as the mice picked up the pasta by mouth but then quickly decreased to baseline with chewing (**Fig. 3n-s**). These results indicate that RFO PT$^{Fezf2}$ and IT$^{PlxnD1}$ activity are associated with coordinated mouth and hand movements of inserting pasta into the mouth and manipulating the pasta, in addition to eating with mouth.

**Division of labor between RFO PN types in oromanual manipulation**

To examine the role of RFO PN types in pasta eating, we suppressed the activity of all projection neurons (PNs$^{Emx1}$), pyramidal tract neurons (PTs$^{Fezf2}$), or intratelencephalic neurons (ITs$^{PlxnD1}$) at different stages of the pasta-eating behavior (**Fig. 4a, Extended Data Fig. 10a**). Bilateral inhibition of these PN types as the mice approached the pasta did not perturb the approach (**Fig. 4b, Extended Data Fig. 10b**). Inhibition of PNs$^{Emx1}$ or PTs$^{Fezf2}$, but not ITs$^{PlxnD1}$, delayed pasta pick-up and increased lick attempts (**Fig. 4b-d, Supplementary Videos 18-20**), likely due to impairments in tongue grasp movements. Following mouth pick-up and transfer of pasta to the hands, inhibition of PNs$^{Emx1}$ and ITs$^{PlxnD1}$, but not PTs$^{Fezf2}$, significantly increased the time taken to make the first bite

(**Fig. 4b, e**). This was due to uncoordinated pasta orienting with the hands and difficulty in making mouth grasp of the pasta (**Fig. 4b, Extended Data Fig. 10c, d**).

Bilateral PN inhibition during the handle-eat stage significantly reduced and delayed pasta biting (**Fig. 4a, f, g**). The deficit was not due to an impairment in biting *per se*. When we presented pasta to the mice in a holding device so that the mice could bite without using their hands, RFO PN inhibition did not interfere with pasta biting (**Extended Data Fig. 11, Supplementary Videos 21-23)**. Multi-faceted quantitative analysis revealed that the deficit of RFO PN inhibition was in oromanual coordination of positioning the pasta in the mouth and of applying force to snap it (**Fig. 4h-p**).

Inhibition of PNs$^{Emx1}$ or ITs$^{PlxnD1}$ during the handle-eat stage produced excessive and uncoordinated hand movements, including unproductive bimanual adjustments (**Fig. 4f, h, Supplementary Videos 24, 25**), which led to increased but ineffective pasta orientation changes before it was grasped in the mouth (**Extended Data Fig. 10e**). Four of six *Emx1* mice were unable to position pasta for a single bite during inhibition (**Fig. 4f, g**). The difficulty in orienting the pasta was confirmed by more variable (PNs$^{Emx1}$) and more vertical orientations (PTs$^{Fezf2}$ and ITs$^{PlxnD1}$) for pasta positioning (**Fig. 4i, j).** Furthermore, PT$^{Fezf2}$ and IT$^{PlxnD1}$ inhibition altered the pasta holding position of the support hand at the time of biting (**Fig. 4k, l, Supplementary Videos 26, 27**), resulting in more vertical pasta bite orientations (**Fig. 4k, m, Extended Data Fig. 12)**. The pasta bite relied critically on the movements of incisors, as the mice always used their incisors to bite even under PN inhibition (**Extended Data Fig. 13**). Finally, PN inhibition disrupted the coordination between the bite and hand movement (i.e., the phase relationship) for snapping the pasta. With respect to the phase of the hand and mouth movements for snapping pasta, PT$^{Fezf2}$ inhibition resulted in delayed bite in relation to downward hand movement and IT$^{PlxnD1}$ inhibition resulted in increased variability of this phase relationship (**Fig. 4n-p**). Altogether, these results indicate that PNs$^{Emx1}$, PTs$^{Fezf2}$, and ITs$^{PlxnD1}$ in the RFO orchestrate the online coordination of oromanual manipulation in positioning the pasta in the mouth and for snapping the pasta.

**RFO PN input-output connectivity patterns reveal cortical and brain networks for oromanual coordination**

To explore RFO-centered brain circuits that contribute to oromanual manipulation for eating, we examined brain-wide input-output connectivity patterns of ITs$^{PlxnD1}$ and PTs$^{Fezf2}$. Anterograde tracing revealed that ITs$^{PlxnD1}$ project bilaterally to primary and secondary motor (MOp, MOs) and sensory (SSp, SSs) orofacial (especially mouth) and forelimb (especially upper limb) areas, and to dorsal agranular insular cortex (AId), visceral cortex (VISC), and the capsular part of the central amygdala nucleus (CEAc) (**Fig. 5a, b, e, Extended Data Fig. 14a, b, d, Supplementary Video 28**). ITs$^{PlxnD1}$ also project bilaterally to the ventrolateral striatum (**Fig. 5b, e, Extended Data Fig. 14b, d**), a region implicated in feeding and food handling [46-48]. In contrast, PTs$^{Fezf2}$ have sparse axon projections to other cortical regions and striatum but project prominently to multiple ipsilateral or contralateral subcortical targets in the thalamus, lateral superior colliculus (lSC), pons, and medulla (**Fig. 5b, e, Extended Data Fig. 14b-d, Supplementary Video 29**). This projection crosses at the pyramidal decussation to innervate the spinal cord (**Extended Data Fig. 14c**). The brainstem targets of PTs$^{Fezf2}$ include multiple command centers for forelimb and orofacial actions such as reaching (PARN) [49,50], grasping (PARN,

MDRN) [49-51], jaw opening (PSV, SPV, IRN) [52-54], licking (PSV, SPV, IRN) [52-54], and whisking (PSV, SPV, IRN) [53-55].

Retrograde monosynaptic rabies tracing revealed that cortical inputs to ITs$^{PlxnD1}$ and PTs$^{Fezf2}$ of the RFO derived almost exclusively from their projection targets (i.e., forelimb and orofacial sensorimotor areas, AId, and VISC; **Fig. 5c-e, Extended Data Fig. 15a, b, d, f, Supplementary Videos 30, 31**). In addition, ITs$^{PlxnD1}$ and PTs$^{Fezf2}$ receive major subcortical inputs from the thalamus, including the ventral anterior-lateral complex and posterior complex (**Fig. 5d, e, Extended Data Fig. 15b, e, f**). Another weak yet reliable subcortical input source is the external segment of the globus pallidus (**Extended Data Fig. 15c, f**).

Collectively, these results reveal several hallmarks of RFO connectivity. Within the cortex, RFO forms a reciprocally connected network involving primary and secondary forelimb and orofacial sensorimotor areas as well as insular and visceral areas, and receives additional inputs from the thalamus and basal ganglia. Whereas ITs$^{PlxnD1}$ target the ventrolateral striatum, thereby contributing to a cortico-striatal-thalamic loop, PTs$^{Fezf2}$ broadcast cortical outputs to all levels of the subcortical structures. This RFO-centered brain network appears well suited to coordinate motor actions across multiple body parts according to online multi-modal sensory inputs (somatosensory and visceral for taste quality) for orchestrating food manipulation during eating. The involvement of VISC and CEAc might further engage valence, incentive, and emotional systems associated with eating.

### DISCUSSION

We have examined the cortical circuit contribution to a naturalistic behavior with inherent ethological relevance, mouse manipulating and eating diverse food items of various configurations and textures. Eating took place in a Mouse Restaurant that provided three dimensional filming and sound recording for capturing, analyzing, and understanding this complex freely-moving behavior. Our analysis describes pasta eating as a sequence of readily identifiable stages, each comprising recognizable action motifs. Our analyses revealed microscale fast movements of hand adjustments, oromanual manipulation, and biting in the context of a macroscale action sequence comprising food retrieval and eating. Because oromanual movements are conserved within rodents and primates [3,4,56], the results are relevant to understanding the complexity of primate and human oromanual movements. Although pose estimation algorithms, such as DeepLabCut [45], can automate the tracking of body parts that are visible, occluded body parts and fine scale movements of the digits are prone to tracking errors. Other challenges include identifying interpretable action motifs and accurately delineating their time course and relationships. Our manual annotation of action motifs from over 4 million video frames presented in the form of actograms provide a ground truth and publicly accessible dataset, which should inspire future machine learning algorithms. Future incorporation of X-ray based fluoroscopy [57] may further capture internal oral actions of the tongue, teeth, and jaw movements. As natural behavior is the “language” of the brain, an understanding of the organization of its syllables and grammar provides a pathway to exploring its neural circuits [21,22,58].

Lesion [59,60], anatomical [61-63], and physiological [64] studies have focused on the role of primary (M1) and secondary (M2) motor cortices in control of relatively isolated and well-trained forelimb movements (e.g. reach and grasp) in primates [61,65] and rodents [66]. These studies have revealed correlations of cortical neuron activity with a range of movement parameters (e.g. force [13,15] and kinematics [14,67]) and have suggested motor cortex as a dynamic system for activity pattern generation [68]. Nevertheless, the role of cortical networks beyond M1 and M2 and the cellular and circuitry basis in orchestrating more complex ethological behaviors in freely moving animals, such as oromanual coordination to place food in the mouth and to manipulate food for biting, have remained poorly understood. Leveraging mouse genetic tools [38], our optogenetic screen with PN-type resolution across the dorsal cortex combined with a non-hypothesis driven assay of forelimb and orofacial movements revealed the RFO and its role in food manipulation. Previous studies of rodent cortex have characterized the anterolateral and more posteromedial areas (ALM and CFA) that control separate orofacial, lick [69,70] vs forelimb reaching [71] movements, in head-fixed animals. The juxtaposition of RFO between these two distinct areas suggests its plausible origin, an evolutionary expansion and overlap of orofacial and forelimb areas shaping a novel area with distinct connectivity patterns to both orofacial and forelimb sensorimotor areas that support a novel behavioral function. In this respect it is noteworthy that stimulation of the macaque precentral gyrus, a region juxtaposed between mouth and hand motor areas, also induces coordinated oromanual movements [30] and the human precentral gyrus contains neurons that respond to mouth stimuli and elicit concurrent hand-to-mouth and mouth movements when stimulated [72]. Together, these findings suggest that a conserved RFO contributes to the food manipulation behavior in rodents and primates including humans.

Among diverse cortical PN classes, IT and PT manifest distinct molecular, anatomical, and physiological properties and represent intracortical processing streams and subcortical output channels, respectively [73]. Leveraging reliable genetic access to $\text{ITs}^{\text{PlxnD1}}$ and $\text{PTs}^{\text{Fezf2}}$ in combination with fine-grained quantitative analysis of an ethological behavior, here we reveal categorical distinctions of IT and PT functions that are highly congruent with and rooted in their anatomical distinctions. As a main RFO output channel, $\text{PTs}^{\text{Fezf2}}$ mainly project unilaterally to multiple subcortical, especially brainstem and spinal, areas implicated in regulating forelimb and orofacial actions [35,50,53]. Within the RFO local circuitry, $\text{ITs}^{\text{PlxnD1}}$ likely provide excitatory inputs to $\text{PTs}^{\text{Fezf2}}$ as ITs are overall upstream of PTs [73,74]. More importantly, $\text{ITs}^{\text{PlxnD1}}$ project bilaterally to several other cortical areas and the ventrolateral striatum, which together may constitute a forelimb-orofacial corticostriatal sensorimotor network. Consistent with this overarching anatomical framework, $\text{PT}^{\text{Fezf2}}$ activation induced contralateral and relatively limited forelimb-orofacial movements. In contrast, $\text{IT}^{\text{PlxnD1}}$ activation elicited bilateral and highly concerted movements that integrate body posture with head, orofacial, forelimb, and digit movements that constitute fictive eating. This is likely achieved by recruiting the extended RFO network that includes forelimb and orofacial sensory and motor areas. Furthermore, whereas $\text{PT}^{\text{Fezf2}}$ inhibition mainly disrupted the execution of skilled oral (e.g. lick-to-retrieve) and forelimb actions, $\text{IT}^{\text{PlxnD1}}$ inhibition predominantly disrupted oromanual coordination. We interpret the lack of a complete impairment of oromanual manipulation by RFO PN inhibition to reflect that a distributed network involving multiple other areas supports this behavior; and redundancy in the network controlling such a fundamental behavior would be highly adaptive, as shown in other motor behaviors [69,75].

Notably, $PTs^{Fezf2}$ and $ITs^{PlxnD1}$ receive inputs from common thalamic and cortical areas, suggesting their coordinated modulation by multi-sensory feedback and motor efference within an RFO-centered cortical network. Thus, contrary to lower-level brainstem command centers that mostly elicited isolated and relatively stereotyped actions and were modulated by local somatosensory inputs from within the same body part [11,53], RFO PNs receive multi-modal sensory inputs, process sensorimotor information within an extended cortico-striatal-thalamic network, and broadcast outputs across subcortical levels to coordinate movements across the body toward orchestrating a dexterous ethological behavior. Importantly, compared with $PT^{Fezf2}$, $IT^{PlxnD1}$ activity in RFO rose earlier after hand withdraw and was more strongly correlated with handle-bite periods, suggesting its crucial role in coordinating oromanual movements for pasta manipulation and biting. Thus it is possible that $ITs^{PlxnD1}$ may select, coordinate, and monitor an overarching feeding program, while $PTs^{Fezf2}$ contribute to the implementation of fine movements. Future work could reveal whether the feeding network described here contributes to the many other rodent behaviors that involve hand mouth cooperation, including self-grooming, pup cleaning, nest building, and play.

Our work establishes an experimental paradigm for exploring the neural circuitry underlying dexterous sensorimotor control in unconstrained animals, with implications for studying primate dexterity [76] and robotic manipulation [77]. We reveal the circuitry implementation of a neural architecture that reflects several core principles of hierarchical motor control [10]. Indeed, partial autonomy of brainstem command centers, information factorization between brainstem and cortical controllers, amortized higher level control, and inter-region communication may together facilitate multi-joint, full-scale body control. This neural architecture is well suited for generating a robust, flexible, and versatile behavioral repertoire toward achieving ethological goals under variable and changing circumstances. Future work could explore whether similar neural circuitry may mediate other complex behaviors in which animals coordinate the action of different body parts, including serial action of human hand and mouth movements used for the languages [6,7].

### ACKNOWLEDGMENTS

We thank Yongjun Qian for making the GtACR1 AAV vector and virus; Joshua Hatfield for helping with STP imaging and video annotations; Priscilla Wu, Joshua Hatfield, and Bao-Xia Han for animal preparation and maintenance; Robert Eifert at the machine shop of CSHL for the fabrication of custom-designed parts; and William Galbavy, Shreyas M. Suryanarayana, and Jason Tucciarone for helpful discussions. We thank Richard Mooney for comments on the manuscript. This work was supported in part by NIMH grants 1DP1MH129954-01 and 5U19MH114821-03 to Z.J.H.

### AUTHOR CONTRIBUTIONS

X.A. and Z. J. H. conceived this study. Z.J.H. supervised the study. X.A. designed the research and performed the majority of the experiments, and analyzed data. K.M. performed STP imaging and anatomy analyses. Y.L. performed in vivo electrophysiology recording. H.M. provided advice for data analysis. X.H.X. analyzed behavioral videos. A.K. and I.Q.W. made contributions to data analysis and discussion. Z.J.H. and X.A. wrote the manuscript with inputs from I.Q.W. and A.K.

## References

1 Iwaniuk, A. N. & Whishaw, I. Q. On the origin of skilled forelimb movements. *Trends Neurosci* **23**, 372-376 (2000). https://doi.org:10.1016/s0166-2236(00)01618-0

2 Sustaita, D. *et al.* Getting a grip on tetrapod grasping: form, function, and evolution. *Biol Rev Camb Philos Soc* **88**, 380-405 (2013). https://doi.org:10.1111/brv.12010

3 Whishaw, I. Q. a. K., J.M. in *Feeding in Vertebrates: Evolution, Morphology, Behavior, Biomechanics* Ch. 6, 159-186 (Springer, 2019).

4 Hirsche, L. A., Cenni, C., Leca, J.-B. & Wishaw, I. Q. Two Types of Withdraw-to-Eat Movement Related to Food Size in Long-Tailed Macaques (Macaca fascicularis): Insights into the Evolution of the Visual Control of Hand Shaping in Anthropoid Primates. *Animal Behavior and Cognition* **9**, 176-195 (2022). https://doi.org:10.26451/abc.09.02.02.2022

5 Barrett, J. M., Raineri Tapies, M. G. & Shepherd, G. M. G. Manual dexterity of mice during food-handling involves the thumb and a set of fast basic movements. *PLoS One* **15**, e0226774 (2020). https://doi.org:10.1371/journal.pone.0226774

6 Kolodny, O. & Edelman, S. The problem of multimodal concurrent serial order in behavior. *Neuroscience & Biobehavioral Reviews* **56**, 252-265 (2015). https://doi.org:https://doi.org/10.1016/j.neubiorev.2015.07.009

7 Lashley, K. S. *The problem of serial order in behavior*. Vol. 21 (Bobbs-Merrill Oxford, 1951).

8 Suryanarayana, S. M., Robertson, B. & Grillner, S. The neural bases of vertebrate motor behaviour through the lens of evolution. *Philos Trans R Soc Lond B Biol Sci* **377**, 20200521 (2022). https://doi.org:10.1098/rstb.2020.0521

9 Arber, S. & Costa, R. M. Connecting neuronal circuits for movement. *Science* **360**, 1403-1404 (2018). https://doi.org:10.1126/science.aat5994

10 Merel, J., Botvinick, M. & Wayne, G. Hierarchical motor control in mammals and machines. *Nat Commun* **10**, 5489 (2019). https://doi.org:10.1038/s41467-019-13239-6

11 Arber, S. & Costa, R. M. Networking brainstem and basal ganglia circuits for movement. *Nat Rev Neurosci* **23**, 342-360 (2022). https://doi.org:10.1038/s41583-022-00581-w

12 Churchland, M. M. *et al.* Neural population dynamics during reaching. *Nature* **487**, 51-56 (2012). https://doi.org:10.1038/nature11129

13 Evarts, E. V. Pyramidal tract activity associated with a conditioned hand movement in the monkey. *J Neurophysiol* **29**, 1011-1027 (1966). https://doi.org:10.1152/jn.1966.29.6.1011

14 Georgopoulos, A. P., Kalaska, J. F., Caminiti, R. & Massey, J. T. On the relations between the direction of two-dimensional arm movements and cell discharge in primate motor cortex. *J Neurosci* **2**, 1527-1537 (1982).

15 Kakei, S., Hoffman, D. S. & Strick, P. L. Muscle and movement representations in the primary motor cortex. *Science* **285**, 2136-2139 (1999). https://doi.org:10.1126/science.285.5436.2136

16 Galinanes, G. L., Bonardi, C. & Huber, D. Directional Reaching for Water as a Cortex-Dependent Behavioral Framework for Mice. *Cell Rep* **22**, 2767-2783 (2018). https://doi.org:10.1016/j.celrep.2018.02.042

17 Hyland, B. Neural activity related to reaching and grasping in rostral and caudal regions of rat motor cortex. *Behav Brain Res* **94**, 255-269 (1998). https://doi.org:10.1016/s0166-4328(97)00157-5

18 Miri, A. *et al.* Behaviorally Selective Engagement of Short-Latency Effector Pathways by Motor Cortex. *Neuron* **95**, 683-696 e611 (2017). https://doi.org:10.1016/j.neuron.2017.06.042
19 Sauerbrei, B. A. *et al.* Cortical pattern generation during dexterous movement is input-driven. *Nature* **577**, 386-391 (2020). https://doi.org:10.1038/s41586-019-1869-9
20 Wang, X. *et al.* Deconstruction of Corticospinal Circuits for Goal-Directed Motor Skills. *Cell* **171**, 440-455 e414 (2017). https://doi.org:10.1016/j.cell.2017.08.014
21 Datta, S. R., Anderson, D. J., Branson, K., Perona, P. & Leifer, A. Computational Neuroethology: A Call to Action. *Neuron* **104**, 11-24 (2019). https://doi.org:10.1016/j.neuron.2019.09.038
22 Miller, C. T. *et al.* Natural behavior is the language of the brain. *Curr Biol* **32**, R482-R493 (2022). https://doi.org:10.1016/j.cub.2022.03.031
23 Galvin, L., Mirza Agha, B., Saleh, M., Mohajerani, M. H. & Whishaw, I. Q. Learning to cricket hunt by the laboratory mouse (Mus musculus): Skilled movements of the hands and mouth in cricket capture and consumption. *Behav Brain Res* **412**, 113404 (2021). https://doi.org:10.1016/j.bbr.2021.113404
24 Gu, Z. *et al.* Control of species-dependent cortico-motoneuronal connections underlying manual dexterity. *Science* **357**, 400-404 (2017). https://doi.org:10.1126/science.aan3721
25 Whishaw, I. Q., Sarna, J. R. & Pellis, S. M. Evidence for rodent-common and species-typical limb and digit use in eating, derived from a comparative analysis of ten rodent species. *Behav Brain Res* **96**, 79-91 (1998). https://doi.org:10.1016/s0166-4328(97)00200-3
26 Barrett, J. M., Martin, M. E. & Shepherd, G. M. G. Manipulation-specific cortical activity as mice handle food. *Current Biology* **32**, 4842-4853.e4846 (2022). https://doi.org:https://doi.org/10.1016/j.cub.2022.09.045
27 Huang, Z. J. Toward a genetic dissection of cortical circuits in the mouse. *Neuron* **83**, 1284-1302 (2014). https://doi.org:10.1016/j.neuron.2014.08.041
28 Luo, L., Callaway, E. M. & Svoboda, K. Genetic Dissection of Neural Circuits: A Decade of Progress. *Neuron* **98**, 256-281 (2018). https://doi.org:10.1016/j.neuron.2018.03.040
29 PENFIELD, W. & BOLDREY, E. SOMATIC MOTOR AND SENSORY REPRESENTATION IN THE CEREBRAL CORTEX OF MAN AS STUDIED BY ELECTRICAL STIMULATION1. *Brain* **60**, 389-443 (1937). https://doi.org:10.1093/brain/60.4.389
30 Graziano, M. S., Taylor, C. S. & Moore, T. Complex movements evoked by microstimulation of precentral cortex. *Neuron* **34**, 841-851 (2002). https://doi.org:10.1016/s0896-6273(02)00698-0
31 Stepniewska, I., Fang, P. C. & Kaas, J. H. Microstimulation reveals specialized subregions for different complex movements in posterior parietal cortex of prosimian galagos. *Proc Natl Acad Sci U S A* **102**, 4878-4883 (2005). https://doi.org:10.1073/pnas.0501048102
32 Brown, A. R. & Teskey, G. C. Motor cortex is functionally organized as a set of spatially distinct representations for complex movements. *J Neurosci* **34**, 13574-13585 (2014). https://doi.org:10.1523/JNEUROSCI.2500-14.2014
33 Neafsey, E. J. *et al.* The organization of the rat motor cortex: a microstimulation mapping study. *Brain Res* **396**, 77-96 (1986). https://doi.org:10.1016/s0006-8993(86)80191-3
34 Tennant, K. A. *et al.* The organization of the forelimb representation of the C57BL/6 mouse motor cortex as defined by intracortical microstimulation and cytoarchitecture. *Cereb Cortex* **21**, 865-876 (2011). https://doi.org:10.1093/cercor/bhq159
35 Mercer Lindsay, N. *et al.* Orofacial Movements Involve Parallel Corticobulbar Projections from Motor Cortex to Trigeminal Premotor Nuclei. *Neuron* **104**, 765-780 e763 (2019). https://doi.org:10.1016/j.neuron.2019.08.032
36 Hira, R., Terada, S.-I., Kondo, M. & Matsuzaki, M. Distinct Functional Modules for Discrete and Rhythmic Forelimb Movements in the Mouse Motor Cortex. *The Journal of Neuroscience* **35**, 13311 (2015). https://doi.org:10.1523/JNEUROSCI.2731-15.2015

37 Harrison, T. C., Ayling, O. G. & Murphy, T. H. Distinct cortical circuit mechanisms for complex forelimb movement and motor map topography. *Neuron* **74**, 397-409 (2012). https://doi.org:10.1016/j.neuron.2012.02.028

38 Matho, K. S. *et al.* Genetic dissection of the glutamatergic neuron system in cerebral cortex. *Nature* **598**, 182-187 (2021). https://doi.org:10.1038/s41586-021-03955-9

39 Arenkiel, B. R. *et al.* In vivo light-induced activation of neural circuitry in transgenic mice expressing channelrhodopsin-2. *Neuron* **54**, 205-218 (2007). https://doi.org:10.1016/j.neuron.2007.03.005

40 Mayrhofer, J. M. *et al.* Distinct Contributions of Whisker Sensory Cortex and Tongue-Jaw Motor Cortex in a Goal-Directed Sensorimotor Transformation. *Neuron* **103**, 1034-1043 e1035 (2019). https://doi.org:10.1016/j.neuron.2019.07.008

41 Allred, R. P. *et al.* The vermicelli handling test: a simple quantitative measure of dexterous forepaw function in rats. *J Neurosci Methods* **170**, 229-244 (2008). https://doi.org:10.1016/j.jneumeth.2008.01.015

42 Whishaw, I. Q. & Coles, B. L. Varieties of paw and digit movement during spontaneous food handling in rats: postures, bimanual coordination, preferences, and the effect of forelimb cortex lesions. *Behav Brain Res* **77**, 135-148 (1996). https://doi.org:10.1016/0166-4328(95)00209-x

43 Whishaw, I. Q. *et al.* The syntactic organization of pasta-eating and the structure of reach movements in the head-fixed mouse. *Sci Rep* **7**, 10987 (2017). https://doi.org:10.1038/s41598-017-10796-y

44 Whishaw, I. Q., Ghasroddashti, A., Mirza Agha, B. & Mohajerani, M. H. The temporal choreography of the yo-yo movement of getting spaghetti into the mouth by the head-fixed mouse. *Behav Brain Res* **381**, 112241 (2020). https://doi.org:10.1016/j.bbr.2019.112241

45 Mathis, A. *et al.* DeepLabCut: markerless pose estimation of user-defined body parts with deep learning. *Nat Neurosci* **21**, 1281-1289 (2018). https://doi.org:10.1038/s41593-018-0209-y

46 Hintiryan, H. *et al.* The mouse cortico-striatal projectome. *Nat Neurosci* **19**, 1100-+ (2016).

47 Salamone, J. D., Mahan, K. & Rogers, S. Ventrolateral Striatal Dopamine Depletions Impair Feeding and Food Handling in Rats. *Pharmacol Biochem Be* **44**, 605-610 (1993).

48 Bakshi, V. P. & Kelley, A. E. Dopaminergic Regulation of Feeding-Behavior .2. Differential-Effects of Amphetamine Microinfusion into 3 Striatal Subregions. *Psychobiology* **19**, 233-242 (1991).

49 Ruder, L. & Arber, S. Brainstem Circuits Controlling Action Diversification. *Annu Rev Neurosci* **42**, 485-504 (2019). https://doi.org:10.1146/annurev-neuro-070918-050201

50 Ruder, L. *et al.* A functional map for diverse forelimb actions within brainstem circuitry. *Nature* **590**, 445-450 (2021). https://doi.org:10.1038/s41586-020-03080-z

51 Esposito, M. S., Capelli, P. & Arber, S. Brainstem nucleus MdV mediates skilled forelimb motor tasks. *Nature* **508**, 351-356 (2014). https://doi.org:10.1038/nature13023

52 Dempsey, B. *et al.* A medullary centre for lapping in mice. *Nat Commun* **12**, 6307 (2021). https://doi.org:10.1038/s41467-021-26275-y

53 Stanek, E. t., Cheng, S., Takatoh, J., Han, B. X. & Wang, F. Monosynaptic premotor circuit tracing reveals neural substrates for oro-motor coordination. *Elife* **3**, e02511 (2014). https://doi.org:10.7554/eLife.02511

54 Takatoh, J. *et al.* Constructing an adult orofacial premotor atlas in Allen mouse CCF. *Elife* **10** (2021). https://doi.org:10.7554/eLife.67291

55 Moore, J. D. *et al.* Hierarchy of orofacial rhythms revealed through whisking and breathing. *Nature* **497**, 205-210 (2013). https://doi.org:10.1038/nature12076

56 Peckre, L. R., Fabre, A.-C., Wall, C. E., Pouydebat, E. & Whishaw, I. Q. Two types of hand withdraw movement to place food in the mouth mediated by somatosensation in 22-species of strepsirrhines. *bioRxiv*, 2022.2003.2013.484147 (2022). https://doi.org:10.1101/2022.03.13.484147

57 Lever, T. E. *et al.* Videofluoroscopic Validation of a Translational Murine Model of Presbyphagia. *Dysphagia* **30**, 328-342 (2015). https://doi.org:10.1007/s00455-015-9604-7

58 Krakauer, J. W., Ghazanfar, A. A., Gomez-Marin, A., MacIver, M. A. & Poeppel, D. Neuroscience Needs Behavior: Correcting a Reductionist Bias. *Neuron* **93**, 480-490 (2017). https://doi.org:10.1016/j.neuron.2016.12.041
59 Lawrence, D. G. & Kuypers, H. G. The functional organization of the motor system in the monkey. I. The effects of bilateral pyramidal lesions. *Brain* **91**, 1-14 (1968). https://doi.org:10.1093/brain/91.1.1
60 Whishaw, I. Q., Pellis, S. M., Gorny, B., Kolb, B. & Tetzlaff, W. Proximal and distal impairments in rat forelimb use in reaching follow unilateral pyramidal tract lesions. *Behav Brain Res* **56**, 59-76 (1993). https://doi.org:10.1016/0166-4328(93)90022-i
61 Strick, P. L., Dum, R. P. & Rathelot, J. A. The Cortical Motor Areas and the Emergence of Motor Skills: A Neuroanatomical Perspective. *Annu Rev Neurosci* **44**, 425-447 (2021). https://doi.org:10.1146/annurev-neuro-070918-050216
62 Munoz-Castaneda, R. *et al.* Cellular anatomy of the mouse primary motor cortex. *Nature* **598**, 159-166 (2021). https://doi.org:10.1038/s41586-021-03970-w
63 Lemon, R. N. Descending pathways in motor control. *Annu Rev Neurosci* **31**, 195-218 (2008). https://doi.org:10.1146/annurev.neuro.31.060407.125547
64 Omrani, M., Kaufman, M. T., Hatsopoulos, N. G. & Cheney, P. D. Perspectives on classical controversies about the motor cortex. *J Neurophysiol* **118**, 1828-1848 (2017). https://doi.org:10.1152/jn.00795.2016
65 Georgopoulos, A. P. & Carpenter, A. F. Coding of movements in the motor cortex. *Curr Opin Neurobiol* **33**, 34-39 (2015). https://doi.org:10.1016/j.conb.2015.01.012
66 Warriner, C. L., Fageiry, S. K., Carmona, L. M. & Miri, A. Towards Cell and Subtype Resolved Functional Organization: Mouse as a Model for the Cortical Control of Movement. *Neuroscience* **450**, 151-160 (2020). https://doi.org:10.1016/j.neuroscience.2020.07.054
67 Hatsopoulos, N. G., Xu, Q. & Amit, Y. Encoding of movement fragments in the motor cortex. *J Neurosci* **27**, 5105-5114 (2007). https://doi.org:10.1523/JNEUROSCI.3570-06.2007
68 Shenoy, K. V., Sahani, M. & Churchland, M. M. Cortical control of arm movements: a dynamical systems perspective. *Annu Rev Neurosci* **36**, 337-359 (2013). https://doi.org:10.1146/annurev-neuro-062111-150509
69 Li, N., Daie, K., Svoboda, K. & Druckmann, S. Robust neuronal dynamics in premotor cortex during motor planning. *Nature* **532**, 459-464 (2016). https://doi.org:10.1038/nature17643
70 Bollu, T. *et al.* Cortex-dependent corrections as the tongue reaches for and misses targets. *Nature* **594**, 82-87 (2021). https://doi.org:10.1038/s41586-021-03561-9
71 Guo, J. Z. *et al.* Cortex commands the performance of skilled movement. *Elife* **4**, e10774 (2015). https://doi.org:10.7554/eLife.10774
72 Desmurget, M. *et al.* Neural representations of ethologically relevant hand/mouth synergies in the human precentral gyrus. *Proc Natl Acad Sci U S A* **111**, 5718-5722 (2014). https://doi.org:10.1073/pnas.1321909111
73 Harris, K. D. & Shepherd, G. M. The neocortical circuit: themes and variations. *Nat Neurosci* **18**, 170-181 (2015). https://doi.org:10.1038/nn.3917
74 Kiritani, T., Wickersham, I. R., Seung, H. S. & Shepherd, G. M. Hierarchical connectivity and connection-specific dynamics in the corticospinal-corticostriatal microcircuit in mouse motor cortex. *J Neurosci* **32**, 4992-5001 (2012). https://doi.org:10.1523/JNEUROSCI.4759-11.2012
75 Morandell, K. & Huber, D. The role of forelimb motor cortex areas in goal directed action in mice. *Sci Rep* **7**, 15759 (2017). https://doi.org:10.1038/s41598-017-15835-2
76 Sobinov, A. R. & Bensmaia, S. J. The neural mechanisms of manual dexterity. *Nat Rev Neurosci* **22**, 741-757 (2021). https://doi.org:10.1038/s41583-021-00528-7
77 Billard, A. & Kragic, D. Trends and challenges in robot manipulation. *Science* **364** (2019). https://doi.org:10.1126/science.aat8414

78 Chen, S. *et al.* High-Throughput Strategy for Profiling Sequential Section With Multiplex Staining of Mouse Brain. *Frontiers in Neuroanatomy* **15** (2021). https://doi.org:10.3389/fnana.2021.771229
79 Tyson, A. L. *et al.* Accurate determination of marker location within whole-brain microscopy images. *Scientific Reports* **12**, 867 (2022). https://doi.org:10.1038/s41598-021-04676-9
80 Niedworok, C. J. *et al.* aMAP is a validated pipeline for registration and segmentation of high-resolution mouse brain data. *Nature Communications* **7**, 11879 (2016). https://doi.org:10.1038/ncomms11879
81 Ayling, O. G. S., Harrison, T. C., Boyd, J. D., Goroshkov, A. & Murphy, T. H. Automated light-based mapping of motor cortex by photoactivation of channelrhodopsin-2 transgenic mice. *Nature Methods* **6**, 219-224 (2009). https://doi.org:10.1038/nmeth.1303
82 Sun, Z. *et al.* Sox2 and Lef-1 interact with Pitx2 to regulate incisor development and stem cell renewal. *Development* **143**, 4115-4126 (2016). https://doi.org:10.1242/dev.138883
83 Qian, Y. *et al.* Programmable RNA sensing for cell monitoring and manipulation. *Nature* **610**, 713-721 (2022). https://doi.org:10.1038/s41586-022-05280-1
84 Martianova, E., Aronson, S. & Proulx, C. D. Multi-Fiber Photometry to Record Neural Activity in Freely-Moving Animals. *J Vis Exp* (2019). https://doi.org:10.3791/60278
85 Han, W. *et al.* Integrated Control of Predatory Hunting by the Central Nucleus of the Amygdala. *Cell* **168**, 311-324.e318 (2017). https://doi.org:10.1016/j.cell.2016.12.027
86 Takeshita, H. *et al.* Modified forelimb grip strength test detects aging-associated physiological decline in skeletal muscle function in male mice. *Scientific Reports* **7**, 42323 (2017). https://doi.org:10.1038/srep42323
87 Zhang, X. *et al.* Genetically identified amygdala–striatal circuits for valence-specific behaviors. *Nat Neurosci* **24**, 1586-1600 (2021). https://doi.org:10.1038/s41593-021-00927-0
88 Wang, B.-S. *et al.* Retinal and Callosal Activity-Dependent Chandelier Cell Elimination Shapes Binocularity in Primary Visual Cortex. *Neuron* **109**, 502-515.e507 (2021). https://doi.org:https://doi.org/10.1016/j.neuron.2020.11.004

### FIGURE LEGENDS

**Figure 1. Cell-type optogenetic activation screen identifies a rostral forelimb orofacial area.**
**a.** PNs comprise hierarchically organized classes, each comprising multiple subpopulations defined by marker gene expression. See **Extended Data Fig. 1** for description of all subpopulations. IT, intratelencephalic; ET, extratelencephalic; PT, pyramidal tract; CT, corticothalamic.
**b.** Schematic of optogenetic motor mapping in head-fixed mice (see Methods). Nose tip is the coordinate origin.
**c.** Schematic of the 3mm x 6mm area mapped by optogenetic activation. See appendix for abbreviations.
**d.** Vector maps of hand (blue) and jaw (red) movement direction (arrow) and distance (arrow length) following activation of $PTs^{Fezf2}$ and $ITs^{PlxnD1}$ across different locations in the boxed area in **c**. Distance was averaged across mice and normalized (hand: 13 *Fezf2* and 7 *PlxnD1* mice; jaw: 11 *Fezf2* and 7 *PlxnD1* mice).
**e.** Representation of forelimb and mouth movements following RFO $PT^{Fezf2}$ and $IT^{PlxnD1}$ stimulation. Arrows indicate movements. See **Supplementary Videos 4, 5**.
**f.** Hand and jaw movement trajectories following RFO $PT^{Fezf2}$ and $IT^{PlxnD1}$ activation (circle in **d**). Black trajectory represents average. Purple triangle in the left panels denotes jaw position at stimulation onset. Circle and square indicate start and end positions, respectively. Colors in trajectories indicate time. Jaw trajectories were normalized relative to the start position (16 and 18 trials for hand and jaw trajectories for $PTs^{Fezf2}$ and 15 trials for $ITs^{PlxnD1}$).
**g.** Changes in hand-to-nose and hand-to-hand distances upon RFO $PT^{Fezf2}$ and $IT^{PlxnD1}$ stimulation (gray shading). Bilateral and contralateral hand-to-mouth movements were induced with $IT^{PlxnD1}$ and $PT^{Fezf2}$ activation, respectively. Darker trajectories depict averages (18 trials for $PTs^{Fezf2}$ and 17 trials for $ITs^{PlxnD1}$).
**h.** Maps of spatial dispersion of hand positions at the end of activation (averaged across 13 *Fezf2* and 7 *PlxnD1* mice).
**i.** Maps of hand-to-nose distance after activation (averaged across 13 *Fezf2* and 7 *PlxnD1* mice).
**j.** Probability of observing contralateral and/or bilateral hand-to-mouth eating-like movement in a 1-s window immediate before (pre) and during RFO stimulation (13 *Fezf2* and 13 *PlxnD1* mice).
**k.** Schematic of RFO location in relation to other motor areas. ALM, anterolateral motor cortex; RFA, rostral forelimb area; CFA, caudal forelimb area.
**l.** Schematic of body movements induced by RFO $PT^{Fezf2}$ and $IT^{PlxnD1}$ activation (blue bars) in freely-moving mice. Red, blue, and green arrow points to the jaw, contralateral and ipsilateral hand respectively. See **Supplementary Videos 6, 7**.
**m.** Single-trial movement trajectories of different body parts induced by $PT^{Fezf2}$ and $IT^{PlxnD1}$ activation in freely-moving mice (circle and square indicate start and end positions and color saturation indicates time).
**n.** Changes in hand-to-nose and hand-to-hand distances following RFO $PT^{Fezf2}$ and $IT^{PlxnD1}$ stimulation (gray shade) in free-moving mice. Bilateral and contralateral hand-to-mouth movements were induced with $IT^{PlxnD1}$ and $PT^{Fezf2}$ activation, respectively. Darker trajectories depict averages (9 trials for $PTs^{Fezf2}$ and 9 trials for $ITs^{PlxnD1}$).
**o.** Distance of contralateral hand to nose following activation in *Fezf2* (n = 3) and *PlxnD1* (n = 5) mice (*$p < 0.05$, two-sided paired t-test).
**p.** Probability of observing contralateral and/or bilateral hand-to-mouth eating-like movement in a 1-s window immediate before (pre) and during RFO stimulation in free-moving mice (3 *Fezf2* and 5 *PlxnD1* mice).
**q.** Probability of observing head-to-hand movement in a 1-s window immediate before (pre) and during RFO stimulation (3 *Fezf2* and 5 *PlxnD1* mice).

Stars indicate Bregma in **c, d**, **h, i**, **k**. Scale bars, 1 mm in **c, d**, **h, i**, **k**. Data are mean ± s.e.m in **j**, **p**, and **q**. Shades around mean denote ± s.e.m in **g**, **n**. A, anterior; P, posterior; D, dorsal; V, ventral; M, medial; L, lateral. The mouse drawing in **b** was adapted from scidraw.io (https://scidraw.io/drawing/44).

**Extended Data Fig. 1 | Different PN types exhibit distinct motor maps.** Related to Fig. 1

**a-d.** Vector maps of hand (blue) and jaw (red) movement direction and distance following optogenetic activation of PNs$^{Emx1}$ (**a**), PNs$^{Thy1\text{-}Tg18}$ (**b**), ITs$^{Tbr2\text{-}E17}$ (**c**), and CTs$^{Tle4}$ (**d**) in different locations of the dorsal cortex. Movement direction and distance along each axis is represented by arrow direction and length (distance averaged and normalized in 2 *Emx1*, 2 *Thy1-Tg18*, 4 *Tbr2-E17*, and 5 *Tle4* mice).

**e.** Maps of hand movement distance (linear travel distance, measured from start to end). No clear hand movement was induced from *Tcerg1l* and *Sema3E* mice (Maps averaged from 2 *Emx1*, 2 *Thy1-Tg18*, 13 *Fezf2*, 5 *Tcerg1l*, 5 *Sema3E*, 7 *PlxnD1*, 4 *Tbr2-E17*, and 5 *Tle4* mice).

**f.** Maps of total jaw movement distance. No clear jaw movement was induced from *Tcerg1l* and *Sema3E* mice (Maps averaged from 2 *Emx1*, 2 *Thy1-Tg18*, 11 *Fezf2*, 5 *Tcerg1l*, 5 *Sema3E*, 7 *PlxnD1*, 4 *Tbr2-E17*, and 5 *Tle4* mice).

**g.** Schematic of in vivo electrophysiological recording with optical tagging.

**h, i.** Light-evoked spikes from electrophysiological recordings in the secondary motor cortex (MOs) in a *Fezf2-* (**h**) or *PlxnD1-CreER;Ai32* (**i**) mouse (5 light pulses were delivered at 10 Hz for 0.5 s).

**j, k.** Vector maps of hand (blue) and jaw (red) movement direction and distance with optogenetic activation of PTs$^{Fezf2}$ (**j**) and ITs$^{PlxnD1}$ (**k**) using different stimulation parameters (compare with maps of 50 Hz, 0.5 s stimulation in **Fig. 1d)**. Movement direction and distance along each axis are represented by arrow direction and length, respectively. Distance was averaged across mice and normalized to that from 10 Hz, 0.5 s stimulation (10 Hz, 0.5 s: n = 5 mice for PTs$^{Fezf2}$ or ITs$^{PlxnD1}$; 50 Hz, 0.1 s: n = 4 mice for PTs$^{Fezf2}$ or ITs$^{PlxnD1}$).

A, anterior; P, posterior; D, dorsal; V, ventral; M, medial; L, lateral. Stars indicate Bregma. Scale bars, 1 mm. The mouse drawing in **g** was adapted from scidraw.io (https://scidraw.io/drawing/44).

**Extended Data Fig. 2 | Characterization of forelimb and jaw movements induced by optogenetic activation of PTs$^{Fezf2}$ and ITs$^{PlxnD1}$.** Related to Fig. 1

**a-c.** Maps of hand linear travel distance measured from start to end (**a**), total travel distance (**b**), and straightness index (**c**) (straightness index = linear travel distance/total travel distance, with smaller index = more rhythmic movement).

**d-f.** Hand trajectories following PT$^{Fezf2}$ activation at three sites as indicated by the three circles in **c.** Red circle at pCFA from 15 trials (**d**); yellow circle at mCFA from 19 trials and trajectory graphs of repetitive movements (**e**); green circle at aCFA from17 trials (**f**). Lighter trajectories represent averages in **d**, **f**. Black trajectories in **e** indicate averages. Circle and square indicate start and end positions respectively in **d, f**. Note: the left hand is lifted and open after stimulation (white arrow in **f**).

**g, h.** 2D projections of hand trajectories from optogenetic activation of PTs$^{Fezf2}$ (**g**) and ITs$^{PlxnD1}$ (**h**). Projected trajectories were color coded based on stimulation location (top right panel in **g**), normalized to the start position (top left panel in **g**), and averaged across 13 *Fezf2* and 7 *PlxnD1* mice. Square indicates end position.

**i-k.** Maps of jaw linear travel distance measured from start to end (**i**), total travel distance (**j**), and straightness index (**k**) (Straightness index = linear travel distance/total travel distance, with smaller index = more rhythmic movement).

**l.** Example jaw trajectories following PT$^{Fezf2}$ or IT$^{PlxnD1}$ activation at two sites as indicated by the two circles in **k** (green circle for 20 PT$^{Fezf2}$ trials; orange circle for 16 IT$^{PlxnD1}$ trials). Black trajectories indicate averages.

Maps were averaged for 13 *Fezf2* and 7 *PlxnD1* mice in **a-c**. Maps were averaged for 11 *Fezf2* and 7 *PlxnD1* mice in **i-k**. Blue bar in **e**, **l** represents stimulation window. Stars indicate Bregma. Scale bars, 1

mm in **a-c**, **g**, **i-k**; 5 mm in **d, f**. A, anterior; P, posterior; D, dorsal; V, ventral; M, medial; L, lateral. The mouse drawing in **g** was adapted from scidraw.io (https://scidraw.io/drawing/44).

**Extended Data Fig. 3 | Activating AAV-targeted PTs$^{Fezf2}$ or ITs$^{PlxnD1}$ in RFO induces hand-to-mouth and mouth movements.** Related to Fig. 1
**a.** Schematic of the approach (left panel) and images of coronal sections showing PTs$^{Fezf2}$ and ITs$^{PlxnD1}$ infected by AAV-DIO-ChR2-eYFP injected into the right RFO (right panels). Scale bar, 1 mm.
**b, c.** Example movement trajectories for the left hand or both hands for 20 PT$^{Fezf2}$ trials (**b**) and 19 IT$^{PlxnD1}$ trials (**c**). Lighter trajectories represent averages. Circle and square indicate start and end positions respectively. Note: **b** (yellow arrow) left hand is closed and **b** (white arrows) jaw opens to the contralateral side after stimulation. Scale bar, 5 mm.
**d, e.** Movement trajectories of the jaw from 16 PT$^{Fezf2}$ trials (**d**) and 8 IT$^{PlxnD1}$ trials (**e**).
**f, g.** 2D projections of left-hand trajectories after stimulation of 5 *Fezf2* mice (**f**) and 6 *PlxnD1* mice (**g**). Square indicates end position. A, anterior; P, posterior; D, dorsal; V, ventral; M, medial; L, lateral.
**h, i.** Movement trajectories of the jaw following stimulation of 5 *Fezf2* mice (**h**) and 6 *PlxnD1* mice (**i**). Movement trajectories were normalized to the start position in **d-i**. Darker trajectories represent averages in **d-i**. Blue bar in **d, e**, **h, i** represents stimulation window.

**Figure 2. RFO is necessary for hand recruitment and oromanual coordination in pasta eating.**
**a.** Schematic of the Mouse Restaurant. A table mounted on an XZ stage brings food to the dining area. Three cameras record movement and a microphone records bite sound. Note: mouse crosses a small elevated step to enter the dining area. See **Extended Data Fig. 4a-c** and Methods for details.
**b.** Pasta-eating schematic showing tracking of different body parts and the pasta (colored dots). Z axis is the dorsal-ventral axis. Mice handle the pasta with a support grasp (purple arrow) and a guide grasp (red arrow).
**c.** Actogram of a mouse retrieving and eating a 15-mm angel-hair pasta piece. Key sensorimotor events (colored and annotated at the bottom) are superimposed upon Z-axis trajectories of nose (gray) and right (dark gray) and left (black) hands throughout the trial.
**d.** Ethogram of pasta eating, which proceeds in a sequence of stages, each consisting of multiple action motifs (top); mice consume pasta in repeated handle-eat bouts. Bottom schematic depicts a typical sequence of four major coordinated hand (blue ring) and oral (red ring) actions in a handle-eat bout. Red arrow in sketches indicates direction of hand movement. Legends for labels in the upper left corner of each drawing are the same as those in **c**.
**e.** Probability distribution of the time of the first hand-adjustment and the first bite in each handle-eat bout (n = 7 mice). Time 0 is the onset of hand withdraw, which marks the start of each bout.
**f.** Neary all hand adjustments (97.0 ± 0.8 %; n = 9 mice) were made with pasta clenched in the mouth, thus involving oromanual coordination.
**g.** Average hand-to-nose distance begins to increase (red arrow) before bite onset (time 0, n = 9 mice).
**h.** Schematic of hand movements immediately before and during pasta bite/snap (arrow indicates movement direction along Z axis; arrow length indicates speed).
**i.** The relationship between up-down hand movements and bite, shown as the Z-axis left-hand trajectory overlaid with bite events. Left ankle was used as the reference to compute the trajectory, which was then band-pass filtered (0.4 - 10 Hz, lower panel) to compute the hand movement phase.
**j.** Probability distribution for the phases of left-hand movement at the time of bites from an example trial in **i**.
**k**, **l**. Average hand movement phase at the time of bite (**k**) and selectivity index of the phases (**l**). The narrower the probability distribution of phases the larger the selectivity index (n = 9 mice).
**m.** Bilateral RFO muscimol infusion resulted in increased pasta drops in each trial (upper; n = 7 mice; ***p < 0.005, two-sided paired t-test) and feeding duration for each pasta piece (lower; n = 8 mice; *p < 0.05, two-sided Wilcoxon signed-rank test).

**n, o.** Probability distribution and cumulative probability of Z-axis positions of the left hand (**n**) and nose (**o**) at the time of bites following saline and muscimol infusion (n = 5 mice; ****p < 0.001, Kolmogorov-Smirnov test). Data from left ankle after saline infusion is shown as reference. Shades around mean denote ± s.e.m in **e**, **g**, **n**, **o**. Data are mean ± s.e.m in **f**, **k-m**. Mouse drawings in **a** were adapted from scidraw.io (https://scidraw.io/drawing/122 and https://scidraw.io/drawing/96).

**Extended Data Fig. 4 | Design of the Mouse Restaurant for studying feeding behavior.** Related to Fig. 2
**a, b.** Schematic of the Mouse Restaurant. A table mounted on an XZ stage (**b**) brings food to the dining area. The food dispenser has two stacked plates, each with a capacity for 24 food items (**b**). A water port in the waiting area allows mice to drink and thus consume more food. Two pairs of infrared (IR) break-beam sensors detect a mouse moving from the waiting to dining area. A door is used to block access to the dining area during food delivery. Three cameras record mouse behavior and a microphone records bite sounds.
**c.** Events and behavioral sequence in Mouse Restaurant and signals used for task control. Behaviors in red were recorded in the dining area.
**d.** Configurations of 15-mm angel-hair pasta when delivered to the dining area. 3D-printed holders were used to load the pasta into the food dispenser in **b**. For configuration 3, mice occasionally retrieved the pasta with the hands instead of the mouth. Trials with hand retrieval were not included in the analysis due to low occurrence.
**e.** Processing of the audio signal for bite detection. Audio signal was band-pass filtered (800-8,000 Hz), rectified, smoothed (5-ms Gaussian window), and thresholded (4 × s.d. above mean) to detect bite events (purple circles). Red rectangle indicates the time window enlarged on the right. Mouse drawings in **a** were adapted from scidraw.io (https://scidraw.io/drawing/122 and https://scidraw.io/drawing/96).

**Extended Data Fig. 5 | Action motifs and sensorimotor events in pasta eating.** Related to Fig. 2
**a-d.** Image sequences showing manually labeled action motifs observed in angel-hair pasta eating. Images in each panel represent the start (left), middle, and end (right) of each action. Red arrows in **a** point to the jaw as it opens to retrieve the pasta. A food-in-mouth event is labeled when the pasta is clearly lifted from the floor (blue arrow in **a**). Arrows in **b** point to the tongue as it brings the pasta into the mouth. Arrows in **c** indicate the upward body movement leading to the sitting posture. After mouth retrieval, mice make reaching movements to grasp pasta with the hands (arrows in **d**).
**e.** Image of a hand-withdraw event, in which mice raise their hands toward mouth (arrow) to start a handle-eat bout after the previous chewing phase. Right panel shows Z-axis trajectory of the right hand before and after a hand-withdraw event, with the cyan line indicating the time of withdraw shown in the left image.
**f, g.** Image sequences showing unimanual (**f**) and bimanual (**g**) adjustments through release and re-grasp movements to reposition the hands on the pasta. Arrows in **f**, **g** point to release (middle) and re-grasp (right) hand movements.

**Extended Data Fig. 6 | Hand adjustment and pasta bite both involve oromanual coordination.** Related to Fig. 2
**a.** Probability distribution of time from hand adjustments to the first bite in each handle-eat bout. The proportion of hand adjustments made before the first bite for 7 mice is 69.2 ± 2.2 %, indicating hand adjustments mainly occur before the first bite. Data are mean ± s.e.m.
**b.** Action sequences for unimanual and bimanual adjustments.
**c, d.** Average Z-axis position (**c**) and speed (**d**) of nose and both hands aligned to bite onset (vertical dashed line) showing that pasta biting involves joint bimanual and jaw movement. Note that the downward hand movement starts before the bite (arrows in **c, d**). The two peaks in **d** is likely due to the breaking of pasta.

**e.** Average hand-nose orientation aligned to bite onset, showing a downward hand movement relative to the nose (mouth) before the bite. The schematic depicts the angle of hand-nose orientation (left panel). Shades around mean denote ± s.e.m in **c-e** for 9 mice.

**Extended Data Fig. 7 | Muscimol inhibition in RFO impairs hand recruitment in pasta eating but not bite and grip force.** Related to Fig. 2

**a.** Schematic of bilateral muscimol infusion into the RFO.

**b.** Representative diffusion pattern of BODIPY-tagged muscimol (red; 1 µl) in the RFO of coronally sectioned (75 µm) tissue stained with DAPI (blue). Scale bar, 500 µm.

**c, d.** Actograms of exemplar trials of a mouse following bilateral saline (**c**) or muscimol (**d**) infusion. In muscimol trials, the mouse usually did not adopt a sitting posture, bit the pasta on the ground without recruiting hands, and often dropped the pasta (red arrows) during eating. In muscimol trials feeding time is prolonged, a mouse sometimes left the dining area without finishing the pasta, or pasta flew out of the dining area after a bite due to uncoordinated oromanual movements. Three exemplar time windows of a muscimol trial are shown in **d1-d3**. Black arrow in **d2** indicates the bite corresponding to the posture image in the bottom right panel. Note the mouse's hunched posture; red arrow in the image points to the nose close to the floor. Also see **Supplementary Videos 12, 13**.

Muscimol inhibition did not impair pasta detection (**e**, n = 8 mice), increased mouth retrieval attempts (**f**, n = 7 mice; *$p < 0.05$, two-sided paired t-test), increased number of trials in which mice consumed the pasta without sitting on haunches (**g**; n = 8 mice, with one mouse never adopting a sitting posture), and did not impair grip force or bite force (**h**; n = 6 mice). Data are mean ± s.e.m. NS, not significant, two-sided paired t-test.

**Figure 3. PT$^{Fezf2}$ and IT$^{PlxnD1}$ activity in RFO correlate with pasta manipulation and eating.**

**a.** Schematic depicting fiber photometry from the right RFO and left aCFA. Star indicates Bregma. Scale bar, 1 mm.

**b.** Coronal sections showing PTs$^{Fezf2}$ and ITs$^{PlxnD1}$ in the RFO and aCFA expressing GCaMP7f from AAV infection. Scale bar, 500 µm.

**c, d.** Single-trial calcium activity traces of PTs$^{Fezf2}$ (**c**) and ITs$^{PlxnD1}$ (**d**) in the RFO (black) and aCFA (gray) of mice eating 15-mm angel-hair pasta. Actograms were overlaid on activity traces. Example time windows are expanded in **c1-c3** and **d1-d3**. Time 0 is the entry of the dining area. The rise of aCFA activity at time 0 (dashed line) correlates with crossing the step for entering the dining area (**Fig. 2a**).

**e, h.** Heat maps of RFO PT$^{Fezf2}$ (**e**) and IT$^{PlxnD1}$ (**h**) population activity aligned to retrieval start (left), hand withdraw (middle), and bite (right). Activity traces were sorted by the earliest hand withdraw (left), chew (middle), and hand adjustment (right) events, respectively.

**f, g, i, j.** Averaged PT$^{Fezf2}$ (**f, g**) and IT$^{PlxnD1}$ (**i, j**) population activity in the RFO and aCFA aligned to retrieval start (**f**, **i**; left panels) and hand withdraw, hand adjustment, bite, and chew (**g**, **j**; left panels). Vertical dashed lines indicate average time to the first hand withdraw in **f**, **i**. Changes in population activity are shown in the right panels. RFO PT$^{Fezf2}$ and IT$^{PlxnD1}$ activity rise after the onset of hand withdraw with a lag (red arrow in the expanded window of **g**, **j)**. n = 6 mice for PTs$^{Fezf2}$ and 6 mice for ITs$^{PlxnD1}$; *$p < 0.05$, ***$p < 0.005$, two-sided paired t-test.

**k.** Time from the onset of hand withdraw to the rise of population activity (n = 6 mice for PTs$^{Fezf2}$ and 6 mice for ITs$^{PlxnD1}$; *$p < 0.05$, two-sided Wilcoxon rank-sum test).

**l.** Correlation between RFO IT$^{PlxnD1}$ population activity and hand-to-nose distance. Boxed time window is expanded on the right. Green arrows indicate the onset of signal rise.

**m.** Averaged correlation coefficient of RFO population activity with hand-to-nose distance shifted in time from a *PlxnD1* mouse. Peak correlation coefficient is shown in the right panel (n = 6 mice for PTs$^{Fezf2}$ and 6 mice for ITs$^{PlxnD1}$; *$p < 0.05$, two-sided Wilcoxon rank-sum test).

**n, o, q, r.** Single-trial calcium activity in the RFO and aCFA as *Fezf2* (**n**) and *PlxnD1* (**q**) mice consumed 1-mm angel-hair pasta without sitting up or hand recruitment. Key sensorimotor events

(colored annotations) were overlaid on the activity traces. Time 0 is the entry to the dining area. Corresponding heat maps (**o**, **r**) were aligned to retrieval start for 1-mm pasta.
**p, s.** Averaged RFO $PT^{Fezf2}$ (**p**) and $IT^{PlxnD1}$ (**s**) population activity aligned to retrieval start for 15-mm and 1-mm angel-hair pasta (n = 7 mice for $PTs^{Fezf2}$ and 3 mice for $ITs^{PlxnD1}$). Vertical dashed lines indicate the average time to establish the sitting posture when eating 15-mm pasta. Activity levels remained high when mice handled and ate 15-mm pasta but declined when eating 1-mm pasta. Shading around mean denotes ± s.e.m in **f, g**, **i, j**, **m**, **p**, **s**. Data are mean ± s.e.m in **f, g**, **i-k**.

**Extended Data Fig. 8 | $PT^{Fezf2}$ and $IT^{PlxnD1}$ activity in aCFA correlate with skilled stepping.** Related to Fig. 3
**a, d.** Single-trial calcium activity of $PTs^{Fezf2}$ (**a**) and $ITs^{PlxnD1}$ (**d**) in the RFO and aCFA during dining area entry and pasta retrieval. Actograms were overlaid on the activity traces.
**b, e.** Heat maps of $PT^{Fezf2}$ (**b**) and $IT^{PlxnD1}$ (**e**) population activity in the RFO and aCFA aligned to crossing the entry step (see **Extended Data Fig. 4a**) and sorted by pasta retrieval start.
**c, f.** Averaged population activity of $PTs^{Fezf2}$ (**c**) and $ITs^{PlxnD1}$ (**f**) in the RFO and aCFA aligned to crossing the step to the dining area (n = 6 mice for $PTs^{Fezf2}$ and 6 mice for $ITs^{PlxnD1}$). Vertical dashed lines indicate average time to the retrieval start. Shading around mean denotes ± s.e.m.

**Extended Data Fig. 9 | Automated identification of handle-bite and chew periods in each handle-eat bout.** Related to Fig. 3
**a.** Hand-to-nose distances plotted in a pasta-eating trial superimposed with alternating handle-bite (green) and chew (red) periods. A two-state hidden Markov model (HMM) was used to identify the handle-bite and chew periods. Vertical dashed lines indicate manually labeled hand-withdraw events. An example time window is enlarged in **a'**.
**b.** Probability distribution of errors between hand-withdraw timestamps labeled manually and computed from HMM. The proportion of |error| < 0.1 s is 84.13 ± 1.96 % (6 sessions from 5 mice).
**c.** Hit rate. Hit rate is the proportion of hand-withdraw events labeled both manually and by HMM over all hand-withdraw events labeled manually (6 sessions from 5 mice). Data are mean ± s.e.m.

**Figure 4. RFO $PTs^{Fezf2}$ and $ITs^{PlxnD1}$ contribute to distinct components of oromanual manipulation.**
**a.** Schematic for optogenetic inhibition of PN types. AAV-DIO-GtACR1-eYFP were injected bilaterally into the RFO. Two inhibition schemes were directed to the retrieval and handle-eat stages, respectively. Green bar indicates 4s inhibition. Time 0 denotes entry into the dining area.
**b.** Actograms (legend shown in **f**) of a mouse in control (upper) and $PN^{Emx1}$ inhibition (lower; green bar) trials. Z-axis trajectories of nose (light gray), right (dark gray) and left (black) hands are shown.
**c, d.** $PN^{Emx1}$ and $PT^{Fezf2}$ inhibition interfered with pasta retrieval, measured as lengthened time from entry to retrieval (**c**) and increased number of retrieval attempts (i.e., retrieval jaw movements) (**d**).
**e.** $PN^{Emx1}$ and $IT^{PlxnD1}$ inhibition delayed the first bite after adoption of a sitting posture.
**f.** Actograms of a mouse at handle-eat stage in control (top) and $PN^{Emx1}$ inhibition (bottom) trials. Z-axis trajectories of nose and two hands are shown. $PN^{Emx1}$ inhibition led to substantially increased hand adjustments but no biting.
**g.** $PN^{Emx1}$, $PT^{Fezf2}$, and $IT^{PlxnD1}$ inhibition resulted in decreased number (left) and increased delay (right) of bites. Purple ticks in top schematic indicate bite events.
**h.** Differences in total hand adjustments (left) and bimanual adjustments (right) made for each bite with PN inhibition compared to control. Note the Y-axis for $PNs^{Emx1}$ is different from that for $PTs^{Fezf2}$ and $ITs^{PlxnD1}$.
**i.** Probability distribution of pasta orientation during handle-eat stage in control and PN inhibition trials; gray trace denotes probability distribution at the time of bite in control trials. Schematic shows exemplar pasta orientation for different conditions. XY plane is the ground plane. Orientation was normalized for each mouse based on the average bite orientation of control trials and then pooled together across mice (****$p < 0.001$, Control vs Inhibition, Kolmogorov-Smirnov test).

**j.** Probability distribution of pasta orientations in control trials was more similar to that of bite orientations in control trials compared with that of pasta orientations in inhibition trials, quantified as difference in Hellinger distance. The smaller the Hellinger distance, the more similar the two probability distributions.

**k.** Schematic of the coordinate system used for analyzing bite posture (left) and average positions of nose, eyes, hands, and pasta at the time of bite from an exemplar mouse (right). Cross indicates inferred bite location inside the mouth. Right eye is the coordinate origin. Transformed X'Y' plane denotes nose and two eyes. X' axis crosses the two eyes plane pointing toward the left eye. Y' axis points to the direction opposite to the nose. Z' axis points outward the mouse's body. Blue and black colors indicate positions with and without inhibition, respectively.

**l.** Average Y'-axis position of support and guide hands at the time of the bite, showing increased hand-to-mouth distance.

**m.** PT$^{Fezf2}$ and IT$^{PlxnD1}$ inhibition led to a vertical shift of bite orientations in the X'Y' plane.

**n.** Probability distributions of the phases of support-hand movement at the time of bites in control and PT$^{Fezf2}$ or IT$^{PlxnD1}$ inhibition trials. Schematic in the left panel depicts the coordination of hand movement with pasta bite/snap.

**o**, **p**. Average hand movement phase at the time of the bite (**o**) and selectivity index of the bite phase (**p**). The narrower the probability distribution of phase the higher the selectivity index. Results from support and guide hands were similar and thus were pooled.

Analyses in **g-p** were carried out for the same 4-s window in **a** for control and inhibition trials. Data are mean ± s.e.m in **k-m**, **o, p**. n = 6 mice for PNs$^{Emx1}$, 8 mice for PTs$^{Fezf2}$, and 9 mice for ITs$^{PlxnD1}$, for the analyses in **c-e**, **g-j**, **l, m**, **o, p**. *$p < 0.05$, **$p < 0.01$, ***$p < 0.005$, ****$p < 0.001$, two-sided paired t-test and two-sided Wilcoxon signed-rank test for **c-e**, **g, h**, **j**, **l, m**, **o, p**.

**Extended Data Fig. 10 | PTs$^{Fezf2}$ and ITs$^{PlxnD1}$ in RFO contribute to oromanual manipulation.** Related to Fig. 4

**a.** Coronal sections showing PNs$^{Emx1}$, PTs$^{Fezf2}$, and ITs$^{PlxnD1}$ in the RFO that were infected by Cre-dependent AAV-DIO-GtACR1-eYFP injection in the corresponding driver mouse. Scale bar, 1 mm.

**b.** PN inhibition did not impact the time taken for pasta detection compared to control trials.

**c.** PN$^{Emx1}$ and IT$^{PlxnD1}$ inhibition increased total hand adjustment preceding the first bite.

**d.** IT$^{PlxnD1}$ inhibition increased bimanual adjustment preceding the first bite.

**e.** PN$^{Emx1}$ and PT$^{Fezf2}$ inhibition led to a significant increase in pasta-orientation change rate during the handle-eat stage. Left panel shows a schematic for quantifying pasta orientations. XY plane is the ground plane.

n = 6 mice for PNs$^{Emx1}$, 8 mice for PTs$^{Fezf2}$, and 9 mice for ITs$^{PlxnD1}$. *$p < 0.05$, **$p < 0.01$, two-sided paired t-test and two-sided Wilcoxon signed-rank test.

**Extended Data Fig. 11 | Inhibiting PTs$^{Fezf2}$ or ITs$^{PlxnD1}$ in RFO does not impair the bite.** Related to Fig. 4

**a.** Schematic of pasta-bite apparatus. Angel-hair pasta is inserted into a metal tube and secured in place with a screw. A small segment (~ 3 mm) of the pasta projects from the tube allowing the mouse to bite off the pasta segment without hand use. Bottom panel shows inhibition scheme, which covers the whole trial period (green bar).

**b.** Number of bites (left panel) and duration taken (right panel) to bite a pasta segment. Purple ticks in the schematic indicate bite events. n = 7 mice for PTs$^{Fezf2}$ and 3 mice for ITs$^{PlxnD1}$. The mouse drawing in **a** was adapted from scidraw.io (https://scidraw.io/drawing/94).

**Extended Data Fig. 12 | Analyses of correlation between hand-mouth distance and pasta orientation.** Related to Fig. 4

**a**. Correlations between the Y'-axis positions of support or guide hands with pasta orientation in the X'Y' plane at the time of the bite in a *Fezf2* mouse. Black and blue lines are linear fittings and yellow line indicates pasta orientation. The support hand is more strongly correlated with the pasta orientation at the time of the bite than is the guide hand. Schematic on the right shows that the more the support hand deviates from the mouth along the Y' axis, the more vertical the X'Y' orientation of the pasta at the time of a bite.

**b.** Correlation coefficient between Y'-axis positions of support and guide hand with pasta orientation in the X'Y' plane at the time of the bite for control trials (3 *Fezf2* and 6 *PlxnD1* mice) and inhibition trials (2 *Fezf2* and 5 *PlxnD1* mice). The support hand is more strongly correlated to pasta orientation than the guide hand, irrespective of PN inhibition.

**Extended Data Fig. 13 | Analyses of pasta bite location.** Related to Fig. 4

**a, b.** Pasta and hand-mouth relationship at the time of the bite in control trials of two exemplar mice. Pasta was positioned to the mouth from right side by the mouse in **a** and from either side by the mouse in **b**. Spatial 3D coordinate system is as described in **Fig. 4k**.

**c.** Schematic showing that if a mouse repeatedly bites pasta at a same location inside its mouth, the new variables x and y, transformed based on the top and bottom coordinates of the pasta, should have a linear relationship (see Methods for details).

**d, e.** Mice bit the pasta at a same location in the mouth (i.e., with the incisors) irrespective of pasta orientation and positioning from the left or right side. Linear fittings for the new variables x and y, transformed from pasta locations presented in **a, b**, are shown. Even in cases when pasta was positioned from either side (**b**), the new variables x and y still have a strong linear relationship (**e**). Yellow lines in **e** indicate the sides of pasta positioning.

**f, g.** Bite location in the mouth in relation to average positions of nose, eyes, hands, and the pasta. Black cross indicates bite location computed from the linear fitting in **d, e**. The bite location corresponds to incisor tips.

**h.** Mice bit pasta with the incisors in control and PN inhibition trials. $R^2$ values of linear fittings (e.g., those in **d, e**) across mice are shown (see Methods for details). n = 8 mice for $PTs^{Fezf2}$ and 8 mice for $ITs^{PlxnD1}$.

**Figure 5. Input-output tracing of $PTs^{Fezf2}$ and $ITs^{PlxnD1}$ in RFO reveal the brain network for oromanual coordination.**

**a.** Schematic for anterograde tracing of $PTs^{Fezf2}$ and $ITs^{PlxnD1}$ in the RFO.

**b.** Axon projection matrix from RFO to 315 ipsilateral and 315 contralateral targets (in rows), each grouped under 12 major categories (left column) for *Fezf2* and *PlxnD1* mice. Color shades in each column represent fraction of total axon signal averaged from 2 *Fezf2* and 2 *PlxnD1* mice.

**c.** Schematic for retrograde monosynaptic rabies tracing of $PTs^{Fezf2}$ and $ITs^{PlxnD1}$ in the RFO.

**d.** Monosynaptic input matrix to RFO from 315 ipsilateral and 315 contralateral targets (in rows), each grouped under 12 major categories (left column) for *Fezf2* and *PlxnD1* mice. Color shades in each column represent fraction of total input cells averaged from 4 *Fezf2* and 5 *PlxnD1* mice.

**e.** A summary wiring diagram of efferent from (solid line) and afferent to (dashed line) $PTs^{Fezf2}$ and $ITs^{PlxnD1}$ in right RFO. Related results are shown in **Extended Data Figs. 14, 15**. See text for detailed description. AId, agranular insular area, dorsal part; APN, anterior pretectal nucleus; CB, cerebellum; CEAc, central amygdalar nucleus, capsular part; CL, central lateral nucleus of the thalamus; CP, caudoputamen; GPe, globus pallidus, external segment; GPi, globus pallidus, internal segment; GRN, gigantocellular reticular nucleus; HPF, hippocampal formation; HY, hypothalamus; IRN, intermediate reticular nucleus; MD, mediodorsal nucleus of the thalamus; MDRN, medullary reticular nucleus; MOp, primary motor area; MOs, secondary motor area; MRN, midbrain reticular nucleus; OLF, olfactory areas; PAL, pallidum; PARN, parvicellular reticular nucleus; PCN, paracentral nucleus; PF, parafascicular nucleus; PG, pontine gray; PO, posterior complex of the thalamus; PPN, pedunculopontine nucleus; PSV, principal sensory nucleus of the trigeminal; SC, superior colliculus; SCm, superior colliculus, motor related; SMT, submedial nucleus of the thalamus; sp, cortical subplate; SPV, spinal nucleus of the trigeminal; SSp-m, primary somatosensory area, mouth; SSp-ul, primary somatosensory area, upper limb; SSs, secondary somatosensory area; STN, subthalamic nucleus; STR, striatum; VAL, ventral anterior-lateral complex of the thalamus; VISC, visceral area; VM, ventral medial nucleus of the thalamus; ZI, zona incerta.

**Extended Data Fig. 14 | Brian-wide projection targets of PTs$^{Fezf2}$ and ITs$^{PlxnD1}$ in RFO.** Related to Fig. 5
**a.** Strategy and timeline for anterograde tracing of PTs$^{Fezf2}$ and ITs$^{PlxnD1}$ in the RFO. TM, tamoxifen.
**b.** Images at the RFO injection site (first row) and selected projection targets: eGFP expression from Flp-activated viral vector (green) and background autofluorescence (red). PTs$^{Fezf2}$ show a weak projection to the cortex and striatum whereas ITs$^{PlxnD1}$ show a strong bilateral projection to the cortex and striatum.
**c.** Images of selected subcortical projection targets of PTs$^{Fezf2}$. Left panels show eGFP expression from Flp-activated viral vector (green) and background autofluorescence (red). Right panels show mCherry expression from Flp-activated viral vector (red) and Nissl staining (blue). PT$^{Fezf2}$ axons form the pyramidal decussation and enter the spinal cord (bottom right panel).
**d.** Schematic depicting main RFO efferent targets for PTs$^{Fezf2}$ and ITs$^{PlxnD1}$. ITs$^{PlxnD1}$ project bilaterally to multiple cortical areas, the ventrolateral striatum, and CEAc. PTs$^{Fezf2}$ project weakly within the cerebral cortex and striatum but project strongly to subcortical structures at all levels. Scale bar, 500 µm.
AId, agranular insular area, dorsal part; APN, anterior pretectal nucleus; CEAc, central amygdalar nucleus, capsular part; CL, central lateral nucleus of the thalamus; CP, caudoputamen; GPe, globus pallidus, external segment; GPi, globus pallidus, internal segment; GRN, gigantocellular reticular nucleus; IRN, intermediate reticular nucleus; MD, mediodorsal nucleus of the thalamus; MdD, medullary reticular nucleus, dorsal part; MDRN, medullary reticular nucleus; MdV, medullary reticular nucleus, ventral part; MOp, primary motor area; MOs, secondary motor area; MRN, midbrain reticular nucleus; PARN, parvicellular reticular nucleus; PCN, paracentral nucleus; PF, parafascicular nucleus; PG, pontine gray; PO, posterior complex of the thalamus; PPN, pedunculopontine nucleus; PSV, principal sensory nucleus of the trigeminal; pyx, pyramidal decussation; SC, superior colliculus; SMT, submedial nucleus of the thalamus; Spd, spinal cord; SPV, spinal nucleus of the trigeminal; SSp-m, primary somatosensory area, mouth; SSp-n, primary somatosensory area, nose; SSp-ul, primary somatosensory area, upper limb; SSs, secondary somatosensory area; STN, subthalamic nucleus; V, motor nucleus of trigeminal; VAL, ventral anterior-lateral complex of the thalamus; VII, facial motor nucleus; VISC, visceral area; VM, ventral medial nucleus of the thalamus; ZI, zona incerta.

**Extended Data Fig. 15 | Brian-wide monosynaptic inputs to PTs$^{Fezf2}$ and ITs$^{PlxnD1}$ in RFO.** Related to Fig. 5
**a.** Strategy and timeline for retrograde monosynaptic rabies tracing of PTs$^{Fezf2}$ and ITs$^{PlxnD1}$ in the RFO. TM, tamoxifen.
**b.** Images at RFO injection site (first row) and selected afferent sources: mCherry expression from rabies viral vector (red) and eGFP expression from Cre-activated starter virus (green). Both PTs$^{Fezf2}$ and ITs$^{PlxnD1}$ receive afferents from cortical areas and the thalamus.
**c.** Images showing input cells in the GPe that monosynaptically connect to PTs$^{Fezf2}$ (left panel) and ITs$^{PlxnD1}$ (right panel) in the RFO.
**d, e.** Proportion of input cells in cortical areas and thalamic nuclei (4 *Fezf2* and 5 *PlxnD1* mice). Data are mean ± s.e.m.

**f.** Schematic depicting input sources to PTs$^{Fezf2}$ and ITs$^{PlxnD1}$ in the RFO from cortical areas, the thalamus, and basal ganglia. Size of the nodes reflect input cell number. Scale bar, 500 µm. AId, agranular insular area, dorsal part; CL, central lateral nucleus of the thalamus; CM, central medial nucleus of the thalamus; CP, caudoputamen; FRP, frontal pole; GPe, globus pallidus, external segment; GU, gustatory areas; MD, mediodorsal nucleus of the thalamus; MOp, primary motor area; MOs, secondary motor area; ORBl, orbital area, lateral part; PCN, paracentral nucleus; PF, parafascicular nucleus; PO, posterior complex of the thalamus; SI, substantia innominata; SMT, submedial nucleus of the thalamus; SSp-bfd, primary somatosensory area, barrel field; SSp-m, primary somatosensory area, mouth; SSp-n, primary somatosensory area, nose; SSp-ul, primary somatosensory area, upper limb; SSp-un, primary somatosensory area, unassigned; SSs, secondary somatosensory area; VAL, ventral anterior-lateral complex of the thalamus; VISC, visceral area; VM, ventral medial nucleus of the thalamus; VPM, ventral posteromedial nucleus of the thalamus.

### SUPPLEMENTARY VIDEOS

**Supplementary Video 1. Optogenetic activation of PTs$^{Fezf2}$ in posterior Caudal Forelimb Area of head-fixed mouse.** Optogenetic activation (PTs$^{Fezf2}$ in pCFA: P 1.125, L 1.125; 0.5 s) in a head-fixed mouse induces a lateral abduction of the left forelimb, with digit opening and extension and elbow extension. Associated facial movements include vibrissae whisking and eyelid opening.

**Supplementary Video 2. Optogenetic activation of PTs$^{Fezf2}$ in medial Caudal Forelimb Area of head-fixed mouse.** Optogenetic activation (PTs$^{Fezf2}$ in mCFA: A 0, L 1.5; 0.5 s) in a head-fixed mouse induces treading (up/down) movements of the left forelimb. With stimulation onset the forelimb is raised by elbow flexion and then lowered by elbow extension (repeated a number of times). Digit flexion follows elbow flexion and digit extension leads elbow extension. Vibrissae whisk with a similar rhythm to the treading movement. The movement has features of a placing response in which a hand attempts to contact and obtain support from a surface.

**Supplementary Video 3. Optogenetic activation of PTs$^{Fezf2}$ in anterior Caudal Forelimb Area of head-fixed mouse.** Optogenetic activation (PTs$^{Fezf2}$ in aCFA: A 0.75, L 1.875; 0.5 s) in a head-fixed mouse induces a stepping or reaching-like forelimb movement. The upward movement involves sequentially, elbow, wrist, and digit flexion followed by extension. At the apex of the movement the limb is in a relaxed posture. Eyelid opening and whisking accompany the movement. The movement has features resembling reaching or stepping.

**Supplementary Video 4. Optogenetic activation of PTs$^{Fezf2}$ in Rostral Forelimb Orofacial area of head-fixed mouse.** Optogenetic activation (PTs$^{Fezf2}$ in RFO: A 1.5, L 2.25; 0.5 s) in a head-fixed mouse induces left hand adduction to the body midline with hand supination and digit flexing and closing. Associated facial movement includes two cycles of jaw opening and closing with lateral leftward tongue protrusion.

**Supplementary Video 5. Optogenetic activation of ITs$^{PlxnD1}$ in Rostral Forelimb Orofacial area of head-fixed mouse.** Optogenetic activation (ITs$^{PlxnD1}$ in RFO: A 1.5, L 2.25; 0.5 s) in a head-fixed mouse induces bilateral digit flexion and closing followed by elbow flexion and adduction of both hands toward the body midline. Adduction and flexion at the shoulders then raise both hands to the mouth. The movement has features of eating or grooming.

**Supplementary Video 6. Optogenetic activation of PTs$^{Fezf2}$ in Rostral Forelimb Orofacial area of free-moving mouse.** Optogenetic activation (PTs$^{Fezf2}$ in RFO: A 1.125, L 1.9; 0.5 s) in a free-moving mouse induces shoulder adduction that carries the left hand, with associated hand supination, toward the body midline. Ipsiversive head turning and lowering bring the snout to contact the radial surface of the hand. The right hand maintains body postural support.

**Supplementary Video 7. Optogenetic activation of ITs$^{PlxnD1}$ in Rostral Forelimb Orofacial area of free-moving mouse.** Optogenetic activation (ITs$^{PlxnD1}$ in RFO: A 2, L 2.625; 0.5 s) in a free-moving mouse interrupts right-hindlimb scratching of the head. The mouse then adopts a sitting posture and concomitant bilateral shoulder adduction brings both hands, with the left hand slightly in the lead, to the body midline. During adduction, the digits flex and close and contact the mouth. At stimulation termination, the hands are replaced on the floor and scratching with the right hindlimb resumes.

**Supplementary Video 8. A dorsal view of the Mouse Restaurant**. The mouse leaves the waiting area, proceeds down a corridor and steps down a small step to enter the dining area to find and eat a food pellet.

The mouse's movements are enabled by opening the "door" to allow access to the dining area and by positioning a "table", containing a food item e.g., food pellet, angel-hair pasta, in the dining area.

**Supplementary Video 9. Sunflower seed eating.** The mouse is able to pick up and eat the sunflower seed in the first session of sunflower seed eating.

**Supplementary Video 10. Husk-intact oat eating.** The mouse enters the dining area, picks up the oat seed from the floor, removes the skin, and eats it.

**Supplementary Video 11. Angel-hair pasta eating after RFO saline infusion.** After saline infusion (Rostral Forelimb Orofacial area), a mouse enters the dining area and finds a 15mm piece of angel-hair pasta. It sniffs and whisks the pasta and then directs its snout to an end of the pasta where with tongue/mouth movements it grasps the pasta with the incisors. Pasta positioning in the mouth induces the adoption of a sitting posture on the haunches and concurrent raising of both hands to grasp the pasta. Bilateral hand adjustments with assistance of the mouth position the pasta in the mouth in an oblique orientation for biting. The pasta is consumed by repeated acts of positioning, biting, and chewing mediated by coordinated oromanual movements. The tracking of different body parts and the pasta are shown.

**Supplementary Video 12. Angel-hair pasta eating after RFO muscimol infusion in Mouse 1.** After muscimol infusion (Rostral Forelimb Orofacial area) Mouse 1 identifies the pasta by sniffing. It is clumsy in picking up the pasta by mouth, does not seek out the end of the pasta for mouth purchase, does not use its tongue/mouth to grasp the pasta and makes little use of its hands for food retrieval from the mouth or pasta manipulation. The pasta is consumed from the floor mainly using mouth movements.

**Supplementary Video 13. Angel-hair pasta eating after RFO muscimol infusion in Mouse 2.** After muscimol infusion (Rostral Forelimb Orofacial area) Mouse 2 identifies the pasta by sniffing, does not seek out the end of the pasta for tongue/mouth purchase, and picks it up in the middle with its mouth. It lifts the hands to grasp the pasta but fails to manipulate the pasta or remove it from its mouth to reorient it into a position for biting. The mouse ends up breaking the pasta in half.

**Supplementary Video 14. Fiber photometry during 15mm angel-hair pasta eating in a *Fezf2* mouse.** Top: A mouse sniffs angel-hair pasta (15mm), grasps it with its tongue, and manipulates it with its mouth and hands into a position for biting. Bottom: Fiber photometry of $\mathrm{PTs}^{\mathrm{Fezf2}}$ in right Rostral Forelimb Orofacial area (RFO: black trace) and left aCFA (grey trace). Legend is the same as that in **Fig. 3c**. Note: relatively greater activity in RFO is associated with oromanual movements of pasta eating.

**Supplementary Video 15. Fiber photometry during 15mm angel-hair pasta eating in a *PlxnD1* mouse.** Top: A mouse sniffs angel-hair pasta (15mm), grasps it with its tongue, and manipulates it with its mouth and hands into a position for biting. Bottom: Fiber photometry of $\mathrm{ITs}^{\mathrm{PlxnD1}}$ in right Rostral Forelimb Orofacial area (RFO: black trace) and left aCFA (grey trace). Legend is the same as that in **Fig. 3c**. Note: RFO exhibits greater activity during eating and activity peaks are associated with oromanual manipulation.

**Supplementary Video 16. Fiber photometry during 1mm angel-hair pasta eating in a *Fezf2* mouse.** Top: A mouse sniffs angel-hair pasta (1mm) and grasps it with its tongue for ingestion (circa 1.7 sec). Bottom: Fiber photometry of $\mathrm{PTs}^{\mathrm{Fezf2}}$ in right RFO (black trace) and left aCFA (grey trace). Legend is the same as that in **Fig. 3n**.

**Supplementary Video 17. Fiber photometry during 1mm angel-hair pasta eating in a *PlxnD1* mouse.** Top: A mouse sniffs angel-hair pasta (1mm) and grasps it with its tongue for ingestion (circa 2.4 sec). Bottom:

Fiber photometry of $ITs^{PlxnD1}$ in right RFO (black trace) and left aCFA (grey trace). Legend is the same as that in **Fig. 3n**.

**Supplementary Video 18. Retrieval stage of pasta eating in a control trial.** A mouse grasps angel-hair pasta (15 mm) by orienting its head so that it can grasp the end of the pasta. The mouse then immediately adopts a sitting posture, uses its hands to take the pasta to help orient the pasta in its mouth. Using oromanual manipulation, it proceeds to bite pieces from the pasta.

**Supplementary Video 19. Optogenetic inhibition of RFO $PNs^{Emx1}$ during retrieval stage of pasta eating.** Optogenetic inhibition of RFO (Rostral Forelimb Orofacial area) $PNs^{Emx1}$ (4 sec duration top left; 15mm-angel hair pasta) starting with mouse entry to the dining area. The mouse does not orient the mouth to the end of the pasta and grasps the pasta with its mouth after the $5^{th}$ attempt. It then immediately adopts a sitting posture and grasps the pasta with its hands, but does not orient its mouth to the end of the pasta but bites the pasta in its middle.

**Supplementary Video 20. Optogenetic inhibition of RFO $PTs^{Fezf2}$ during retrieval stage of pasta eating.** Optogenetic inhibition of RFO (Rostral Forelimb Orofacial area) $PTs^{Fezf2}$ (4 sec duration top left; 15mm-angel hair pasta) begins as the mouse enters the dining area. The mouse orients its mouth to the end of the pasta but only grasps the pasta after the $6^{th}$ attempt. Once the pasta is grasped, the mouse immediately adopts a sitting posture and orients its mouth to the end of pasta to bite.

**Supplementary Video 21. Pasta-bite test.** Control trial in the pasta-bite test, in which a *Fezf2* mouse approaches, detects, orients its mouth, and successfully bites a piece of angel-hair pasta that projects horizontally from a holder located in the aperture.

**Supplementary Video 22. Pasta-bite test with RFO $PTs^{Fezf2}$ inhibition**. Optogenetic inhibition ($PTs^{Fezf2}$; whole trial) of Rostral Forelimb Orofacial area does not affect approach, detection, head orient, and successful bite of a piece of angel-hair pasta that projects horizontally from a holder located in the aperture.

**Supplementary Video 23. Pasta-bite test with RFO $ITs^{PlxnD1}$ inhibition**. Optogenetic inhibition ($ITs^{PlxnD1}$; whole trial) of Rostral Forelimb Orofacial area does not affect approach, detection, mouth orient, and successful bite of a piece of angel-hair pasta that projects horizontally from a holder located in the aperture.

**Supplementary Video 24. Control pasta-eating in the handle-eat stage.** The mouse makes coordinated oromanual movements to position and bite the 15mm-angel hair pasta.

**Supplementary Video 25. Optogenetic inhibition of RFO $PNs^{Emx1}$ during handle-eat stage of pasta-eating.** Optogenetic inhibition ($PNs^{Emx1}$, 4 s - top white bar, 15mm-angel hair pasta) of the Rostral Forelimb Orofacial area disrupts pasta handling. Mouth orienting to the end of the pasta is interrupted so that eventual biting is directed to the middle of the pasta. Posture is maintained and hand manipulation continues.

**Supplementary Video 26. Optogenetic inhibition of RFO $PTs^{Fezf2}$ during handle-eat stage of pasta-eating.** Optogenetic inhibition of the Rostral Forelimb Orofacial area ($PTs^{Fezf2}$, 4 s - top white bar, 15mm-angel hair pasta) alters pasta holding position of the support hand and impairs oromanual manipulation to bite/snap the pasta.

**Supplementary Video 27. Optogenetic inhibition of RFO $ITs^{PlxnD1}$ during handle-eat stage of pasta-eating.** Optogenetic inhibition of the Rostral Forelimb Orofacial area ($ITs^{PlxnD1}$, 4 s - top white bar, 15mm angel hair pasta) alters pasta holding position of the support hand and impairs oromanual coordination to bite/snap the pasta.

**Supplementary Video 28**. **Anterograde axon projections of RFO ITs$^{PlxnD1}$.** Whole-brain stacked images of Flp-activated eGFP virus injection in a *PlxnD1* mouse showing axons in cortical areas (e.g., MOs, MOp, SSp-ul, SSp-m, SSp-n, SSs, AId, and VISC) and the ventrolateral part of the striatum of both hemispheres. In addition, ITs$^{PlxnD1}$ projected bilaterally to the capsular part of the central amygdala nucleus. MOs, secondary motor area; MOp, primary motor area; AId, agranular insular area, dorsal part; SSp-ul, primary somatosensory area, upper limb; SSp-m, primary somatosensory area, mouth; SSp-n, primary somatosensory area, nose; SSs, secondary somatosensory area; VISC, visceral area; RFO, Rostral Forelimb Orofacial area; IT, intratelencephalic.

**Supplementary Video 29**. **Anterograde axon projections of RFO PTs$^{Fezf2}$.** Whole-brain stacked images of Flp-activated eGFP virus injection in a *Fezf2* mouse showing axons mainly in the ventrolateral part of the ipsilateral striatum, thalamus (e.g., VAL, PO, and PF), lateral superior colliculus, pons, and medulla. The axons were mainly in the contralateral medulla and eventually crossed at the pyramidal decussation to innervate the spinal cord. VAL, ventral anterior-lateral complex of the thalamus; PO, posterior complex of the thalamus; PF, parafascicular nucleus; RFO, Rostral Forelimb Orofacial area; PT, pyramidal tract.

**Supplementary Video 30**. **Retrograde monosynaptic input tracing of RFO PTs$^{Fezf2}$.** Whole-brain stacked images of rabies virus injection in a *Fezf2* mouse showing input cells mainly from cortical areas (e.g., MOs, MOp, SSp-ul, SSp-m, SSp-n, SSs, AId, and VISC) and the thalamus (e.g., VAL, PO, PCN, and VM). MOs, secondary motor area; MOp, primary motor area; AId, agranular insular area, dorsal part; SSp-ul, primary somatosensory area, upper limb; SSp-m, primary somatosensory area, mouth; SSp-n, primary somatosensory area, nose; SSs, secondary somatosensory area; VISC, visceral area; VAL, ventral anterior-lateral complex of the thalamus; PO, posterior complex of the thalamus; PCN, paracentral nucleus; VM, ventral medial nucleus of the thalamus; RFO, Rostral Forelimb Orofacial area; PT, pyramidal tract.

**Supplementary Video 31. Retrograde monosynaptic input tracing of RFO ITs$^{PlxnD1}$.** Whole-brain stacked images of rabies virus injection in a *PlxnD1* mouse showing input cells mainly from cortical areas (e.g., MOs, MOp, SSp-ul, SSp-m, SSp-n, SSs, AId, and VISC) and the thalamus (e.g., VAL, PO, PCN, and VM). MOs, secondary motor area; MOp, primary motor area; AId, agranular insular area, dorsal part; SSp-ul, primary somatosensory area, upper limb; SSp-m, primary somatosensory area, mouth; SSp-n, primary somatosensory area, nose; SSs, secondary somatosensory area; VISC, visceral area; VAL, ventral anterior-lateral complex of the thalamus; PO, posterior complex of the thalamus; PCN, paracentral nucleus; VM, ventral medial nucleus of the thalamus; RFO, Rostral Forelimb Orofacial area; IT, intratelencephalic.

### METHODS

**Animals.** Adult male and female mice bred onto a C57BL/6J background were used in the experiments. Mice were housed under a 12-h light-dark cycle (7.00 to 19.00 light), with room temperature at 22 °C and humidity at 50%. The experimental procedures were approved by the Institutional Animal Care and Use Committee of Cold Spring Harbor Laboratory (CSHL) and Duke University and performed in accordance with the US National Institutes of Health (NIH) guidelines.

The *Fezf2-CreER*, *Fezf2-Flp*, *PlxnD1-CreER*, *Sema3E-CreER*, *Tcerg1l-CreER*, *Tbr2-CreER*, and *Tle4-CreER* knock-in mouse driver lines, in which the expression of the inducible Cre recombinase (CreER) or Flp are driven by endogenous promoters, were generated as previously described [38]. The *Emx1-Cre* knock-in mouse driver line was purchased from Jackson Laboratory (005628). The *Thy1-ChR2* transgenic line 18 (*Thy1-Tg18*) was a gift from Dr. Dinu Florin Albeanu at CSHL. The *Rosa26-loxp-stop-loxp-flpo* (*LSL-Flp*) reporter mice were in-house derived. The *Ai14* (*Rosa26-LSL-tdTomato*), *Ai32* (*Rosa26-LSL-ChR2-eYFP*), *Ai148* (*TIGRE-TRE2-LSL-GCaMP6f-LSL-tTA2*), and *Snap25-LSL-2A-EGFP-D* reporter mice were purchased from Jackson Laboratory (*Ai14*, 007908; *Ai32*, 024109; *Ai148*, 030328; *Snap25-LSL-2A-EGFP-D*, 021879). CreER or Cre driver mice were crossed with *Ai32* or *Ai148* reporter mice for optogenetic stimulation and fiber photometry respectively.

**Viral vectors.** The AAV9-Ef1a-DIO-ChR2-eYFP and AAV9-syn-FLEX-jGCaMP7f-WPRE were purchased from Addgene. The AAV2/8-Ef1a-fDIO-TVA-mCherry, AAV2/8-Ef1a-fDIO-TVA-eGFP, and AAVDJ-DIO-GtACR1-eYFP were produced in house. The AAV8-hSyn-FLEX-TVA-P2A-eGFP-2A-oG and EnVA-dG-Rabies-mCherry were purchased from Salk GT3 Vector Core (La Jolla, California). All viral vectors were aliquoted and stored at -80 °C until use.

**Stereotaxic surgery.** Mice, anesthetized with isoflurane (2-5 % at the beginning and 0.8-1.2 % for the rest of the surgical procedure), were positioned in a stereotaxic frame and their body temperature was maintained at 34-37 °C with a heating pad. Lidocaine (2%) was applied subcutaneously to the scalp prior to surgery. Ketoprofen (5 mg/kg) was administered intraperitonially (IP) as an analgesic before and after surgery. A vertical incision was made through the scalp and connective tissue to expose the dorsal surface of the skull. The skin was pushed aside, and the skull surface was cleared using saline. A digital mouse brain atlas, linked to the stereotaxic frame, guided the identification and targeting of different brain areas (Angle Two Stereotaxic System, Leica Biosystems). Coordinates for injections and/or implantations in the RFO were 1.5-1.88 mm anterior from Bregma, 2.25-2.63 mm lateral from the midline; aCFA: 0.5 mm anterior from Bregma, 1.5 mm lateral from the midline.

For viral injection, a small burr hole was drilled in the skull and brain surface was exposed. A pulled glass pipette, with a tip of 20-30 μm, containing the viral suspension was lowered into the brain. A 300-400 nl volume was delivered at a rate of 10-30 nl/min using a Picospritzer (General Valve Corp). The pipette remained in place for 5 min, to prevent backflow, prior to retraction. Injections were made at depths of 0.3 and 0.6 mm for *PlxnD1* mice, 0.5 and 0.8 mm for *Fezf2* mice, and 0.3, 0.6, and 0.8 mm for *Emx1* mice. The incision was closed with Tissueglue (3M Vetbond) or 5/0 nylon suture thread (Ethilon Nylon Suture, Ethicon). The mice were kept warm on a heating pad during recovery.

For optogenetic activation, an optical fiber (diameter 200 μm; NA, 0.22 or 0.39) was implanted in the right RFO. For optogenetic inhibition, optical fibers (diameter 400 μm; NA, 0.37) were implanted bilaterally in the RFO. For fiber photometry, optical fibers (diameter 200 μm; NA, 0.39) were implanted in the right RFO and left aCFA. The optical fibers were implanted with their tips touching the brain surface. For three *Fezf2* mice used for fiber photometry, the optical fibers were implanted at a depth of 400 and 500 µm from the cortical surface in the aCFA and RFO respectively. For drug infusion, two stainless-steel guide cannulae (24-gauge, 62002, RWD Life Science) were implanted bilaterally into the RFO 0.3 mm below the brain surface. To fix the implants to the skull, a silicone adhesive (Kwik-Sil, WPI) was applied to cover the hole, followed by a layer of dental cement (C&B Metabond, Parkell), black instant adhesive (Loctite 426), and dental cement (Ortho-Jet, Lang Dental). A titanium head bar was fixed to the skull near Lambda using dental cement. A plug cannula (62102, RWD Life Science) was inserted into the guide cannula to prevent clogging and reduce the risk of infection.

For thin-skull window preparation, the skull of the right hemisphere was thinned in a 6 mm × 3 mm window preparation (+/- 3 mm AP from Bregma, 3 mm lateral to Bregma) using a micro drill until brain vasculature became visible after saline application. Bregma was then marked in blue. A thin layer of translucent dental cement (C&B Metabond, Parkell) was applied to the thinned skull, followed by nail polish. A titanium head bar was fixed to the skull near Lambda using dental cement (Ortho-Jet, Lang Dental).

**Tamoxifen induction.** Tamoxifen (T5648, Sigma) was dissolved in corn oil (20 mg/ml) by stirring with a magnetic bead at room temperature overnight or by applying a sonication pulse for 60 s, followed by constant rotation overnight at 37 °C. Individual aliquots (1.5 ml each) were stored at 4 °C. For viral injected CreER driver mice, tamoxifen induction was performed via intraperitoneal injections at a dose of 100 mg/kg. The first induction was given one day after the viral injection and subsequent inductions were given once every 2 days for 2-3 times. To drive the reporter gene expression, mice were injected (IP, 100-200 mg/kg) 2-3 times at P21, 28, and/or 35. To identify embryonic day 17 (E17) for tamoxifen induction, female and male mice were housed together overnight and females were checked for a vaginal plug between 8-9 am the following morning. Following light isoflurane anesthesia, pregnant females were given oral gavage administration of tamoxifen (dose: 3 mg / 30 g of body weight) at gestational day E17.

**Immunohistochemistry.** Adult mice were anaesthetized (using 2.5% Avertin) and intracardially perfused with 25-30 ml PBS followed by 25-30 ml 4% paraformaldehyde (PFA) in 0.1 M PB. After overnight post-fixation at 4 °C, brains were rinsed three times with PBS and sectioned at a thickness of 50-75 µm with a Leica 1000s vibratome. Sections were placed in a blocking solution containing 10% normal goat serum (NGS) and 0.1% Triton-X100 in PBS1X for 1.5 h, then incubated overnight at 4 °C, or room temperature, with primary antibodies diluted in the blocking solution. Sections were rinsed three times (10 min each) in PBS and incubated for 2h at room temperature with corresponding secondary antibodies. Sections were dry-mounted on slides using Fluoromount-G mounting medium (0100-01, SouthernBiotech). Primary antibodies of chicken anti-GFP (1:1,000 or 1:500, Aves, GFP-1020) and rabbit anti-RFP (1:1,000 or 1:500, Rockland Pharmaceuticals, 600-401-379) were used. Alexa Fluor dye-conjugated IgG secondary antibodies (1:500, Molecular Probes, catalog number A11039 for goat anti-chicken 488, A11012 for goat anti-rabbit 594) were used. In some instances, sections were incubated with Neurotrace fluorescent Nissl stain (1:300, Molecular Probes, catalog number N21479) or DAPI (1:1,000, Thermo Scientific, 62248) in secondary antibody. Imaging was performed using a Zeiss Axioimager M2 fluorescence microscope, Zeiss LSM 780 or 710 confocal microscopes, or Zeiss Axio Vert.A1 microscope.

**Retrograde monosynaptic rabies tracing.** To map brain-wide monosynaptic inputs onto $PTs^{Fezf2}$ and $ITs^{PlxnD1}$ in the RFO, we first injected the *Fezf2-CreER* or *PlxnD1-CreER* mice with the starter virus of

AAV8-hSyn-FLEX-TVA-P2A-eGFP-2A-oG (0.3 μl) in the right RFO. Tamoxifen induction was performed via intraperitoneal injections at a dose of 100 mg/kg, once every 2 days for 3 times (the first induction was one day after the starter virus injection). Three weeks after the AAV injection, mice were injected in the RFO with EnVA-dG-Rabies-mCherry (0.4 μl). Brain tissue was prepared for histologic examination 7-10 days after the rabies virus injection.

Rabies injected brains were imaged either with a Zeiss Axioimager M2 fluorescence microscope or with whole-brain STP tomography. For the wide-field epi-fluorescence imaging, 75-μm coronal sections were obtained across the anteroposterior axis of the brain and every other section was quantitatively analyzed. RFP-labeled (that is, rabies-labeled) input cells were automatically detected, and brain slices were registered to the reference Allen Brain Atlas using Serial Section Registration (http://atlas.brainsmatics.org/a/ssr2021) [78]. False- and miss-labeled cells were corrected manually. Data are presented as the ratio between the number of RFP-labeled cells in each brain area and the total number of RFP-labeled cells across the entire brain.

**Whole-brain STP tomography.** We used the whole-brain STP tomography pipeline previously described [38]. Perfused and post-fixed brains, prepared as described above, were embedded in 4% oxidized agarose in 0.05 M PB, cross-linked in 0.2% sodium borohydrate solution (in 0.05 M sodium borate buffer, pH 9.0-9.5). The entire brain was imaged in coronal sections with a 20× Olympus XLUMPLFLN20XW lens (NA 1.0) on a TissueCyte 1000 microscope (Tissuevision) with a Chameleon Ultrafast-2 Ti:Sapphire laser (Coherent). EGFP/EYFP or tdTomato/mCherry signals were excited at 910 nm or 920 nm, respectively. Whole-brain image sets were acquired as series of 12 (x) × 16 (y) tiles with 1 μm × 1 μm sampling for 230-270 z sections with a 50-μm z-step size. Images were collected by two PMTs (PMT, Hamamatsu, R3896) using a 560 nm dichroic mirror (Chroma, T560LPXR) and band-pass filters (Semrock, FF01-680/SP-25). The image tiles were corrected to remove illumination artifacts along the edges and stitched as a grid sequence. Image processing was completed using Fiji software with linear level adjustments applied only to entire images.

**Axon detection from whole-brain STP data.** For axon projection mapping, PN axon signal based on cell-type specific viral expression of EGFP or EYFP was filtered by applying a square root transformation, histogram matching to the original image, and median and Gaussian filtering using Fiji/ImageJ software to maximize signal detection while minimizing background auto-fluorescence [38]. A normalized subtraction of the autofluorescent background channel was applied and the resulting thresholded images were converted to binary maps. Projections were quantified as the fraction of pixels in each brain structure relative to each whole projection.

**Registration of whole-brain STP image datasets.** Registration of brain-wide datasets to the Allen reference Common Coordinate Framework (CCFv3) was performed either by 3D affine registration followed by a 3D B-spline registration using Elastix software, according to established parameters [38] or by brainreg software [79,80]. For axon projection analysis, we registered the CCFv3 to each dataset to report pixels from axon segmentation in each brain structure without warping the imaging channel.

**Axon-projection and monosynaptic-input diagrams from whole-brain imaging data.** To generate diagrams of axon projections and monosynaptic inputs for a given driver line, axon- and cell-detection outputs from all individual experiments were compared (sorting the values from high to low) and analyzed side-by-side with low-resolution image stacks (and the CCFv3 registered to the low-resolution dataset for brain area definition) to get a general picture of the injection and high-resolution images for specific brain areas.

**In vivo electrophysiology and data analysis.** The surgery is described in previous sections. To provide a ground reference, an M1 screw connected to a silver wire (A-M systems) was implanted into the skull above the left visual cortex during surgery.

Before the first recording session, a craniotomy was made in the secondary motor cortex (MOs, A: 1.6 mm; L: 1.4 mm) under isoflurane anesthesia. A silicon probe (ASSY-37 H4, Cambridge NeuroTech, or A1x32-5mm-25-177, A4x8-5mm-100-200-177, NeuroNexus) was slowly lowered into the cortex using a micromanipulator (MP-285, Sutter Instrument). A silicone adhesive (Kwik-Sil, World Precision Instruments) was applied over the craniotomy to stabilize the exposed brain. The brain was allowed to settle for 15-30 minutes before recording began. Voltage signals were continuously recorded at 32 kHz from all 32 channels of the silicon probe by a Digital Lynx 4SX recording system (Neuralynx). Raw data were collected and saved using Cheetah software. The neuronal activity in different channels was band-pass filtered (300-6,000 Hz) for real-time visualization. For optical tagging, 473-nm blue light pulses (2-ms or 5-ms duration) at different frequencies (0.1 or 10 Hz) were delivered through an optical fiber over the craniotomy. At the end of the session, the probe was retracted, and the craniotomy was covered with the silicone adhesive to allow a subsequent recording session on the following day.

Raw data were rearranged according to probe configurations, median-subtracted across channels and time, and saved in 16-bit binary files for spike detection and sorting using Kilosort software (https://github.com/cortex-lab/KiloSort). We used default parameters from KiloSort2 for spike detection and sorting, and further manually curated the spike clusters in phy2 (https://github.com/cortex-lab/phy). Sorted data were analyzed using custom MATLAB codes. Several parameters were taken into consideration for cluster quality control: spike shape, average spike firing frequency (> 0.05), amplitude (> 60 mV), contamination rate (< 0.2), and isolation distance (> 18). Peri-event raster plots and histograms were used to visualize the light evoked spikes from Ai32 crossed mice.

**Optogenetic motor mapping.** Optogenetic motor mapping techniques were adapted from those previously described (**Fig. 1b**) [36,37,81]. We briefly anesthetized the mice with isoflurane (2%) to attach a reflective marker on the back of the left hand and to paint their jaw red. Mice were then transferred into a tube, head fixed on a mapping stage, and allowed to fully recover from the anesthesia before stimulation began. The thin-skull window was cleaned with a duster and covered with silicone oil (378399, Sigma-Aldrich). We used a 2D motorized stage (ASI, MS-2000) controlled by MATLAB programs to localize the stimulation at different cortical sites. A 473-nm laser (5-ms pulses, 10 or 50 Hz, 5-20 mW) was used to pseudo-randomly stimulate (100-ms or 500-ms duration) a grid of 128 programmed sites at intervals of 375 µm. A plano-convex lens (focal length (FL) = 250 mm, LA1301-A, Thorlabs) coupled with a SLR photon lens (Voigtlander Nokton, 35 mm FL, f/1.2) was used to collimate the laser beam. The diameter of the laser beam was ~230 µm ($1/e^2$ diameter). A dichroic mirror (Chroma T495lpxr-UF2, round, 2-inch diameter) was used to guide the laser beam to the tissue. Two SLR lenses (the same Nokton 35 mm FL and a Nikkor 105 mm FL, f/2.0, AF), coupled front to front, were used to image the thin-skull window onto the CMOS sensor of a camera (MV1-D1312-40-G2-12, Photonfocus) with a pixel size of 2.67 µm. Bregma was used as the coordinate reference. Each site was stimulated 15-20 times per session. The inter stimulation interval was 2 s. Two cameras (FL3-U3-13E4C-C, FLIR), positioned at the front and the side of the animal, were used to take videorecordings at a frame rate of 100 Hz. The videos were time aligned by TTL signals controlled by the MATLAB programs. The video and TTL-signal states were acquired using workflows in Bonsai software. Four LED light lamps were used for illumination (2 for each camera). After mapping, the thin-skull window was covered with silicone sealant (Kwik-Cast, WPI) for protection and later mapping.

**In vivo optogenetic activation.** For head-fixed activation, mice injected with ChR2 virus in the right RFO were prepared and video recorded as described above. A fiber coupled laser (5-ms pulses, 5-20 mW; $\lambda$ = 473 nm) was used to apply stimulation at 10, 20, 30, and 50 Hz and constantly for 0.5 s. For free-moving activation, mice with an optical fiber implanted in the right RFO were placed into an acrylic activity box (14 cm × 14 cm × 16.5 cm, L × W × H). A 473-nm laser (5-ms pulses, 5-20 mW) coupled to a rotary joint (RJPFL2, Thorlabs) was used to apply stimulation at 10, 20, and 50 Hz and constantly for 0.5 s. Three cameras (FL3-U3-13S2C-CS, FLIR) were used to take video records at a frame rate of 120 Hz from two sides and the bottom of the activity box. LED light lamps adjacent to each camera provided illumination.

**Video analysis for motor mapping and optogenetic activation.** Videos of behavior from the motor mapping and head-fixed activation were analyzed either with MATLAB programs or DeepLabCut [45]. The two cameras were calibrated using the Camera Calibrator App in MATLAB. For hand and jaw tracking in MATLAB, the images were smoothed with a Gaussian low-pass filter (size 9, sigma 1.8). The centroid of the reflective marker on the hand and the tip of the painted jaw were detected by a combination of brightness and hue thresholding, then tracked by a feature-based tracking algorithm (PointTracker in Computer Vision Toolbox). The tracking results were validated manually and errors were corrected accordingly. For DeepLabCut training, 525 images were used from the frontal video record and 700 images were used from the side video record to track the movements of the jaw and hands. Trials in which mice made spontaneous movements before stimulation onset (within 0.5 s) were excluded from the analyses, based on either manual examinations or setting threshold (3 × s.d. from the mean) on average speed and acceleration distributions of all trials.

For videos obtained from free-moving activation, the tracking of different body parts was performed using DeepLabCut. The network was trained with 800 images. Eight body parts (left and right eyes, hands, ankles, nose, and tail base) were labeled in the images. The behavioral videos and tracking results were visualized and analyzed in a custom-written MATLAB app. Tracking errors were corrected using the app.

The spatial dispersion (SD) of hand positions at the end of optogenetic activation was computed as follows:

$$SD = \frac{1}{n}\sum_{i=1}^{n}\sqrt{(x_i-\bar{x})^2+(y_i-\bar{y})^2+(z_i-\bar{z})^2},$$

where $\bar{x}$, $\bar{y}$, and $\bar{z}$ are mean positions for each axis.

To quantify the hand-to-mouth movement induced by optogenetic activation in the head-fixed animals, we examined the videos from the cortical site that featured the shortest distance between the hand and the nose following the activation (coordinates for 13 *Fezf2* mice: 1.24 ± 0.12 mm anterior from Bregma, 2.39 ± 0.09 mm lateral from the midline; 7 *PlxnD1* mice: 1.66 ± 0.14 mm anterior from Bregma, 2.46 ± 0.11 mm lateral from the midline). Criteria for labeling hand-to-mouth movement were: (1) a forelimb movement that brought the hand to the mouth; (2) a wrist supination; (3) a flexion of the digits. Any intervening grooming movements were not scored as hand-to-mouth movements. We labeled head-to-hand movement by examining the videos from free-moving animals receiving optogenetic stimulation. A head movement that brought the mouth toward the hand contralateral to the stimulation site was defined as a head-to-hand movement.

**Angel-hair pasta eating behavior.** Mice given ad libitum access to water were food restricted until they reached 80 to 85% of their initial body weight. Food restriction began at least 4 days after surgery. Each day during food restriction, the mice were fed food pellets (0.3-3.5 g of 14 mg Dustless Precision Pellets, F05684, Bio-Serv) to maintain body weight. Most behavioral experiments began after the third day of food restriction, at which time body weights had reached the target level.

Feeding behavior of mice was studied in an automated Mouse Restaurant (**Fig. 2a, Extended Data Fig. 4a, b**). The apparatus has two areas, a dining area (10 cm × 10 cm × 15 cm, dimensions L × W × H) and a waiting area (15 cm × 15 cm × 15 cm), connected by a corridor (24 cm × 4 cm × 15 cm). Food items were placed on a 3D-printed plate mounted on an XZ motorized stage. The plate was moved from the dining area to a food dispenser by two stepper motors (PD42-3-1070, Trinamic Motion Control). The food dispenser, made with two stacked 3D-printed plates, placed a food item onto the table. Each of the two plates, driven by a stepper motor (NEMA-17, 324, Adafruit), could hold 24-food items, such that 48-food items can be provided in the dining area in each session. An acrylic door in the corridor was opened by a servo motor (D625MW, Hitec) to allow access to the dining area from the waiting area. In this way, mice left the waiting area entered the dining area to eat, and after eating returned to the waiting area where water was accessible from a water port in a corner. Two pairs of infrared (IR) break-beam sensors (2168, Adafruit) installed at each end of the corridor detected the movement direction of the mice. An elevated step fixed between the corridor and the dining area kept food items from being swept out of the dining area. Once mice returned to the waiting area, the door was closed, a new food item was presented and the next trial began. A session ended after all 48-food items were presented or 40 minutes elapsed. The apparatus was controlled by codes running on an Arduino Mega 2560 Rev3 (A000067, Arduino) with three shields (IO sensor shield, DFR0165, DFRobot; LCD and motor shield, 772 and 1438, respectively, Adafruit). An Arduino Uno Rev3 (A000066, Arduino), with three shields (screw shield, DFR0171, DFRobot; LCD and data logging shield, 772 and 1141, respectively, Adafruit), took signals from the IR break-beam sensors to control a laser for optogenetic stimulation and to send TTL signals to recording devices for time alignment.

The mice were pretrained to shuttle between the waiting area and the dining area for one session each day for 2-3 days, were they consumed 30-48, 20-mg pellets (Dustless Precision Pellets, F0163, Bio-Serv). On the day before a pasta-eating session, the mice were familiarized with 0.5 g of angel-hair pasta in their home cage. On the following day, 15-mm pasta pieces were loaded into the food dispenser before the session by inserting them into 3D-printed holders (10 mm × 10 mm × 2 mm, L × W × H, with a 1.5 mm diameter hole in the middle, **Extended Data Fig. 4b, d**). In the test sessions, the mice consumed 24-48 pieces of 15-mm pasta. During 1-2 sessions, concurrent fiber photometry was obtained. During 6-8 sessions, optogenetic inhibition was applied. At the completion of the 15-mm pasta-eating tests, mice received a training session in which they received 1-mm angel-hair pasta that had been manually placed on the table. Then photometry was obtained over two sessions during which 15-mm and 1-mm pasta lengths, cut using a custom-designed plate, were presented in an alternating order.

**Pasta-bite test.** Following the 15-mm pasta-eating sessions, mice used in optogenetic inhibition sessions were given a pasta-bite test. A 20-mm piece of angel-hair pasta was inserted into a metal tube and fixed in place by a screw. The apparatus was located in an aperture (15 mm × 15 mm × 15 mm, L × W × H) made of clear acrylic (**Extended Data Fig. 11a**). A mouse inserted its head into the aperture and bit off pieces of pasta (~ 3 mm). One training session was given before the inhibition session. After each trial, mice returned to the waiting area, a new piece of pasta was placed in the holder, and the next trial began.

The mice learned to bite the pasta in the first session after which, 1-2 sessions were given with optogenetic inhibition.

**Video recording for pasta eating and data analysis.** Three cameras, one on each side of the dining area, video recorded (120 Hz, FL3-U3-13S2C-CS, FLIR) behavior in the dining area. Each camera was fitted with a varifocal lens (T10Z0513CS, Computar). The cameras were time aligned by the TTL signals sent by the Arduino Uno Rev3. The videos and TTL-signal states were acquired using workflows in Bonsai software. Four LED light lamps placed around the dining area provided illumination. For fiber photometry, long-pass filters (590 nm, FGL590S, Thorlabs) were installed on the light lamps. A webcam (C920, Logitech) was installed on a post to monitor the mice from a dorsal perspective.

The cameras were calibrated using Camera Calibrator App in MATLAB and 12,623 images were pooled to train a deep neural network for tracking using DeepLabCut. Ten body parts (left and right eyes, hands, ankles, nose, tongue, jaw, and tail base) and the pasta (top, center, and bottom) were labeled in the images. The behavioral videos and tracking results were visualized and analyzed in a custom-written MATLAB app.

In the app, we labeled action motifs and sensorimotor events manually through a frame-by-frame analysis (**Extended Data Fig. 5**). Images from all three cameras were displayed for each frame and about 4 million frames were labelled. We identified the start and end frames for the following action motifs: jaw retrieve, tongue lick, left- and right-hand reach, sit, left- and right-hand adjustment. The start frame defined movement initiation and the stop frame defined movement completion. Food-in-mouth events were labeled once the pasta was clearly lifted from the floor by the mouth. A hand withdraw event was labeled as a mouse raised its hands toward the mouth after chewing. A feeding-end event was labeled when mice lowered their bodies to the floor after food consumption. For saline and muscimol infusion sessions, events in which pasta was dropped were additionally labeled.

In addition to manual labelling, hand-withdraw events and the onsets of chewing were identified with a two-state hidden Markov model (https://www.cs.ubc.ca/~murphyk/Software/HMM/hmm.html) using normalized distances of the left- and right-hand to the nose. The model was trained on data from each session with ten random initializations. Only distances from the first bite to the last bite in each trial were used for the training. The model with the largest log likelihood was used to classify the handle-bite and chew states. A hand-withdraw event was computed as the transition point from a chew state to a handle-bite state. Conversely, the onset of chewing was computed as the transition point from a handle-bite state to a chew state.

To estimate the time of pasta detection, we first computed the distances from the nose to the top, center, and bottom of the pasta at each onset of pasta retrieval in the control trials. The shortest nose-to-pasta distance at each pasta-retrieval onset was saved. The average shortest distance was used as the pasta-detection distance and computed separately for each mouse. The first time point at which the shortest nose-to-pasta distance drops below the pasta-detection distance was used as pasta-detection time and computed for all trials.

We used Hellinger distance to quantify the similarity between two probability distributions of pasta orientations. For two probability distributions $P = (p_1,\ldots,p_k)$ and $Q = (q_1,\ldots q_k)$, their Hellinger distance is computed as:

$$H(P,Q) = \frac{1}{\sqrt{2}}\sqrt{\sum_{i=1}^{k}(\sqrt{p_i} - \sqrt{q_i})^2}.$$

To analyze the phase of hand movements, we first computed the Z-axis movement trajectory using ankle position as a reference. The movement trajectory was band-pass filtered (0.4 - 10 Hz) with forward-backward-zero-phase FIR filters. Hilbert transform was then used on the filtered trajectory to acquire instantaneous phases of the movement. A vector summation was used to obtain the average phase at the time of a bite and the selectivity index of phases:

$$R = \frac{\sum_k e^{i\theta_k}}{k},$$

where $\theta_k$ is the phase at the time of a bite. The complex phase and amplitude of the resultant R represent the average phase and selectivity index respectively.

To determine how the bite was made, we used a head-based coordinate system (**Fig. 4k**). The right eye defined the coordinate origin. The X'Y' plane was defined by the plane where the nose and both eyes reside. The X' axis crossed both eyes pointing toward the left eye; the Y' axis pointed to the direction that was opposite to the nose; the Z' axis pointed outward from the mouse's body. The coordinates of the left eye, left and right hands, nose, and top and bottom of the pasta in the head-based coordinate system were computed by linear transformations. The analysis of bite location indicated that a mouse bites the pasta at a same location inside its mouth (**Extended Data Fig. 13a, b**). We thus assumed that the coordinates of the corresponding bite location in a 2D plane (e.g., X'Y' plane) are (a, b). Let (x1, y1) and (x2, y2) be the top and bottom coordinates of the pasta, respectively, at the time of a bite (**Extended Data Fig. 13c**). Given the line defined by the pasta passes through the bite location, we have,

$$m = \frac{y1-y2}{x1-x2}, (1)$$

$$\frac{y1-b}{x1-a} = m, (2)$$

where m is the slope of the line. Let y = y1-mx1, x = -m, Eq. 2 can be rewritten as y = ax+b, which indicates that the x and y, transformed from the top and bottom coordinates of the pasta, have a linear relationship. The experimental data support the assumption that mouse bites the pasta at a same location inside its mouth (**Extended Data Fig. 13d, e, h**). The coordinates (a, b) of the bite location were computed by linear fitting and plotted on the corresponding 2D planes (**Extended Data Fig. 13f, g**). Using a diagram of the oral cavity of a mouse [82], our computation of bite location corresponded with the incisor tips.

**Sound recording and signal analysis.** A microphone (AT803, Audio-Technica) on the wall of the dining area picked up the sound of pasta biting. The audio signal from the microphone was amplified (Studio Channel, PreSonus) and digitized at 96,000 Hz by a multifunctional I/O device (PCIe-6323, National Instruments) controlled by MATLAB programs. The TTL signal sent out by the Arduino Uno Rev3 was recorded for time alignment. To detect bite events, the audio signal was band-pass filtered (Butterworth filter, 800-8,000 Hz), rectified, smoothed with a Gaussian window (5 ms), and thresholded (3-5 × s.d. from the mean).

**Muscimol infusion.** After performing 1-2 sessions of 15-mm angel-hair pasta eating, mice were infused with 0.9% saline or muscimol (1 mg/ml, in 0.9% saline) bilaterally into the RFO for two consecutive pasta-eating sessions (saline given for one session, muscimol for the next, or vice versa). Mice were head fixed on a stage and the two hemispheres were infused sequentially after removal of the plug cannula. The injection cannula (28-gauge, 62202, RWD Life Science) connected to a microsyringe (80330, Hamilton) was inserted into the guide cannula to deliver 0.5 or 1 µl of the solution at a rate of 0.1 or 0.2 µl/min by a syringe pump (Legato 130, KD Scientific). After the infusion, the injection cannula was left in place for 5 min to prevent backflow and then retracted, and the plug cannula was reinserted. At the end of the experiments, muscimol diffusion in the brain tissue was determined in two mice by infusing fluorescent muscimol (BODIPY TMR-X Conjugate, 1 mg/ml, dissolved in 50% dimethyl sulfoxide in 0.9% saline; M23400, ThermoFisher Scientific) bilaterally into the RFO (0.5 and 1 µl in the left and right hemispheres respectively), with the same infusion procedure used for the pasta-eating sessions.

**In vivo optogenetic inhibition.** The implanted optical fibers were cleaned using alcohol swab sticks and connected to a rotary joint (FRJ_1x2i_FC-2FC, Doric Lenses) with two fiber patch cords (fiber core diameter, 200 µm; RWD Life Science). A fiber coupled laser (5-15 mW; $\lambda$ = 532 nm) controlled by the Arduino Uno Rev3 was used for the stimulation. For 15-mm angel-hair pasta eating sessions, the laser was turned on for 4 s at mouse entry into the dining area (early inhibition, from 0 s to 4 s) or 4 s after entry (late inhibition, from 4 s to 8 s). Thus, the late inhibition targeted oromanual handling. For late-inhibition sessions, control and inhibition trials in which mice didn't adopt a sit posture within 4 s were excluded from analysis. For the pasta-bite test, the laser was turned on at entry into the dining area and turned off at the return to the waiting area. Stimulation was given pseudo-randomly for half of the trials in each session.

**Fiber photometry and data analysis.** A commercial fiber photometry system (Neurophotometrics) was used to record calcium activity of $PTs^{Fezf2}$ and $ITs^{PlxnD1}$ in the right RFO and left aCFA at 20 Hz. A branching patch cord (fiber core diameter, 200 µm; Doric Lenses) connected the photometry system with the implanted optical fibers. The intensity of the blue light ($\lambda$ = 470 nm) for GCaMP excitation was adjusted to 20-50 µW at the tip of the patch cord. A violate light ($\lambda$ = 415 nm, 20-50 µW at the tip) was used to acquire the isosbestic control signal to detect calcium-independent artifacts. Emitted signals were band-pass filtered and focused on the sensor of a CMOS camera. Photometry signals and behavioral events were aligned based on the TTL signals generated by the Arduino Uno Rev3. Mean values of signals from the two ROIs were calculated and saved by using Bonsai software, and were exported to MATLAB for further analysis.

The recorded photometry signals were processed as previously described [83,84]. A baseline correction of each signal was made using the adaptive iteratively reweighted Penalized Least Squares (airPLS) algorithm (https://github.com/zmzhang/airPLS) to remove the slope and low frequency fluctuations in the signals. The baseline corrected signals were then standardized (Z-score) on a trial-by-trial basis using the median value and standard deviation of the baseline period (10.6 s, while mouse is waiting for food delivery). The standardized 415-nm excited isosbestic signal was fitted to the standardized 470-nm excited GCaMP signal using robust linear regression. The standardized isosbestic signal was scaled using parameters of the linear regression and regressed out from the standardized GCaMP signal to obtain calcium dependent signal.

To compute the correlation coefficient between the hand-to-nose distance and GCaMP signal, we used the average of left- and right-hand to nose distances. The hand-to-nose distance was low-pass filtered (5 Hz), shifted forward and backward in time, and downsampled to compute the correlation coefficients of different time lags from -1 s to 1 s. Data in the time window from the first bite to the last bite were used for the correlation analysis.

**Grip and bite strength analysis.** Bite strength was measured using an accurate single point load cell system (OEM Style Single Point Load Cells, Omega) [85]. The system was connected to a custom-built mouth piece with dimensions (H = 3 mm × W = 5 mm × L = 15 mm) based on the incisor morphology of adult C57BL6/J mice. Output signals were amplified (IN-UVI, Omega), digitized via a National

Instruments board (PCIe-6323), and fed into a custom MATLAB-based computer interface. A mouse was constrained in a 60-ml plastic tube with an opening on the top to accommodate the implanted cannulae. To prevent the mouse from escaping, a plunger was inserted to loosely confine the mouse. A mouth piece was presented manually and moved slowly at 0.5-1 cm/sec toward the mouth so that the mouse could bite it. Bite strength was measured for 3-4 sessions (120-240 sec per session) for each mouse.

Forelimb grip strength was measured using a custom-designed 3D-printed metal bar (L = 8 cm, diameter = 1.2 mm) attached to an accurate single point load cell system (OEM Style Single Point Load Cells, Omega). The record of the output signal was acquired following a previously described protocol [86]. In each of 3-4 tests, when a mouse grasped the bar with both hands, its tail was slowly pulled downward with increasing pressure so that the mouse was required to increase its resistance.

**Statistics and data presentation.** Significance levels used in the analyses and figures were: *$P < 0.05$, **$P< 0.01$, ***$P < 0.005$, ****$P < 0.001$, with data presented as mean ± s.e.m., except where otherwise indicated. In the statistical comparisons, data normality was checked with quantile plots and a Shapiro-Wilk normality test in MATLAB. Non-normally distributed data were subsequently compared with non-parametric tests. All statistical tests were two-tailed and adjustments were made for multiple comparisons. No statistical methods were used to predetermine sample size, but our sample sizes are similar to those reported in previous publications [87,88].

**Data availability.** The data that support the findings of this study are available from the corresponding author upon reasonable request.

**Code availability.** Custom-written scripts used in this study are available in a GitHub repository at https://github.com/XuAn-universe/Publication-source-code.

### LIST OF ABBREVIATIONS

ACAd, anterior cingulate area, dorsal part

AId, agranular insular area, dorsal part

APN, anterior pretectal nucleus

CB, cerebellum

CEAc, central amygdalar nucleus, capsular part

CL, central lateral nucleus of the thalamus

CP, caudoputamen

FRP, frontal pole

GPe, globus pallidus, external segment

GPi, globus pallidus, internal segment

GRN, gigantocellular reticular nucleus

GU, gustatory areas

HPF, hippocampal formation

HY, hypothalamus

IRN, intermediate reticular nucleus

MD, mediodorsal nucleus of the thalamus

MdD, medullary reticular nucleus, dorsal part

MDRN, medullary reticular nucleus

MdV, medullary reticular nucleus, ventral part

MOp, primary motor area

MOs, secondary motor area

MRN, midbrain reticular nucleus

OLF, olfactory areas

ORBl, orbital area, lateral part

PAL, pallidum

PARN, parvicellular reticular nucleus

PCN, paracentral nucleus

PF, parafascicular nucleus

PG, pontine gray

PL, prelimbic area

PO, posterior complex of the thalamus

PPN, pedunculopontine nucleus

PSV, principal sensory nucleus of the trigeminal pyx, pyramidal decussation

RSPagl, retrosplenial area, lateral agranular part

RSPd, retrosplenial area, dorsal part

SC, superior colliculus

SCm, superior colliculus, motor related

SI, substantia innominata

SMT, submedial nucleus of the thalamus sp, cortical subplate

Spd, spinal cord

SPV, spinal nucleus of the trigeminal

SSp-bfd, primary somatosensory area, barrel field

SSp-ll, primary somatosensory area, lower limb

SSp-m, primary somatosensory area, mouth

SSp-n, primary somatosensory area, nose

SSp-tr, primary somatosensory area, trunk

SSp-ul, primary somatosensory area, upper limb

SSp-un, primary somatosensory area, unassigned

SSs, secondary somatosensory area

STN, subthalamic nucleus

STR, striatum

V, motor nucleus of trigeminal

VAL, ventral anterior-lateral complex of the thalamus

VII, facial motor nucleus

VISa, anterior area

VISam, anteromedial visual area

VISC, visceral area

VISp primary visual area

VISpm, posteromedial visual area

VISrl, rostrolateral visual area

VM, ventral medial nucleus of the thalamus

VPM, ventral posteromedial nucleus of the thalamus

ZI, zona incerta

## Figure 1

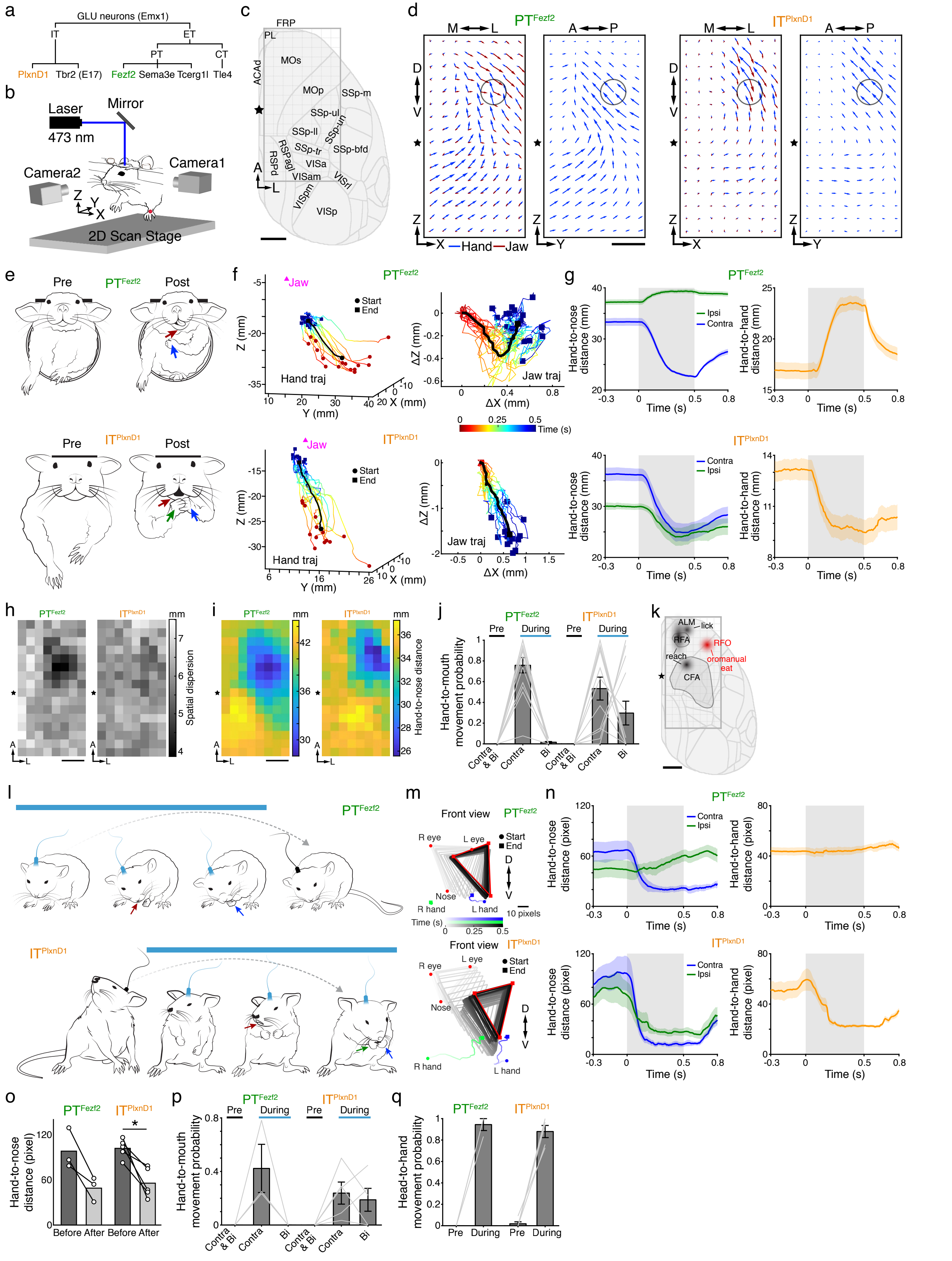

## Extended Data Figure 1

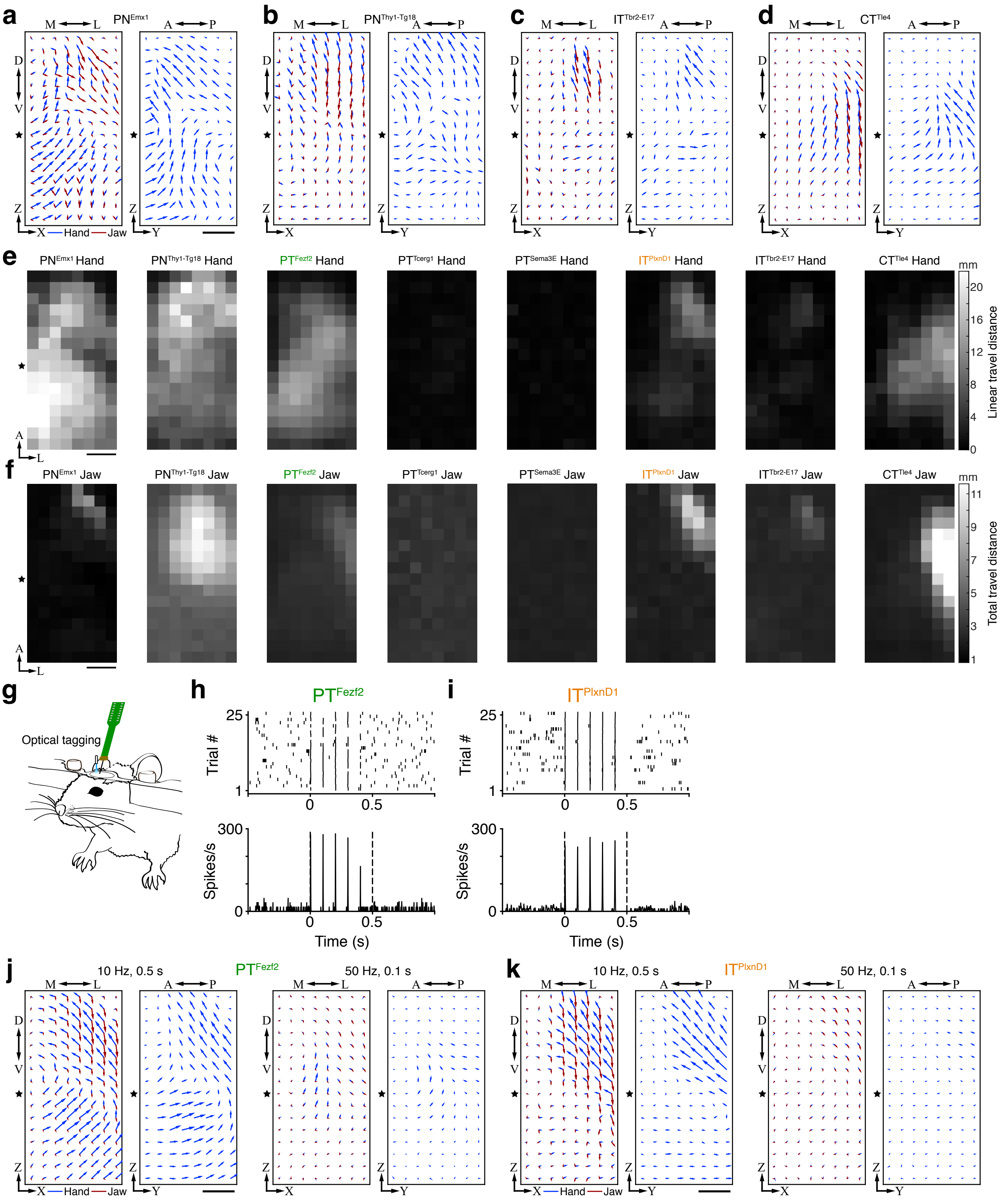

## Extended Data Figure 2

Figure 1-2

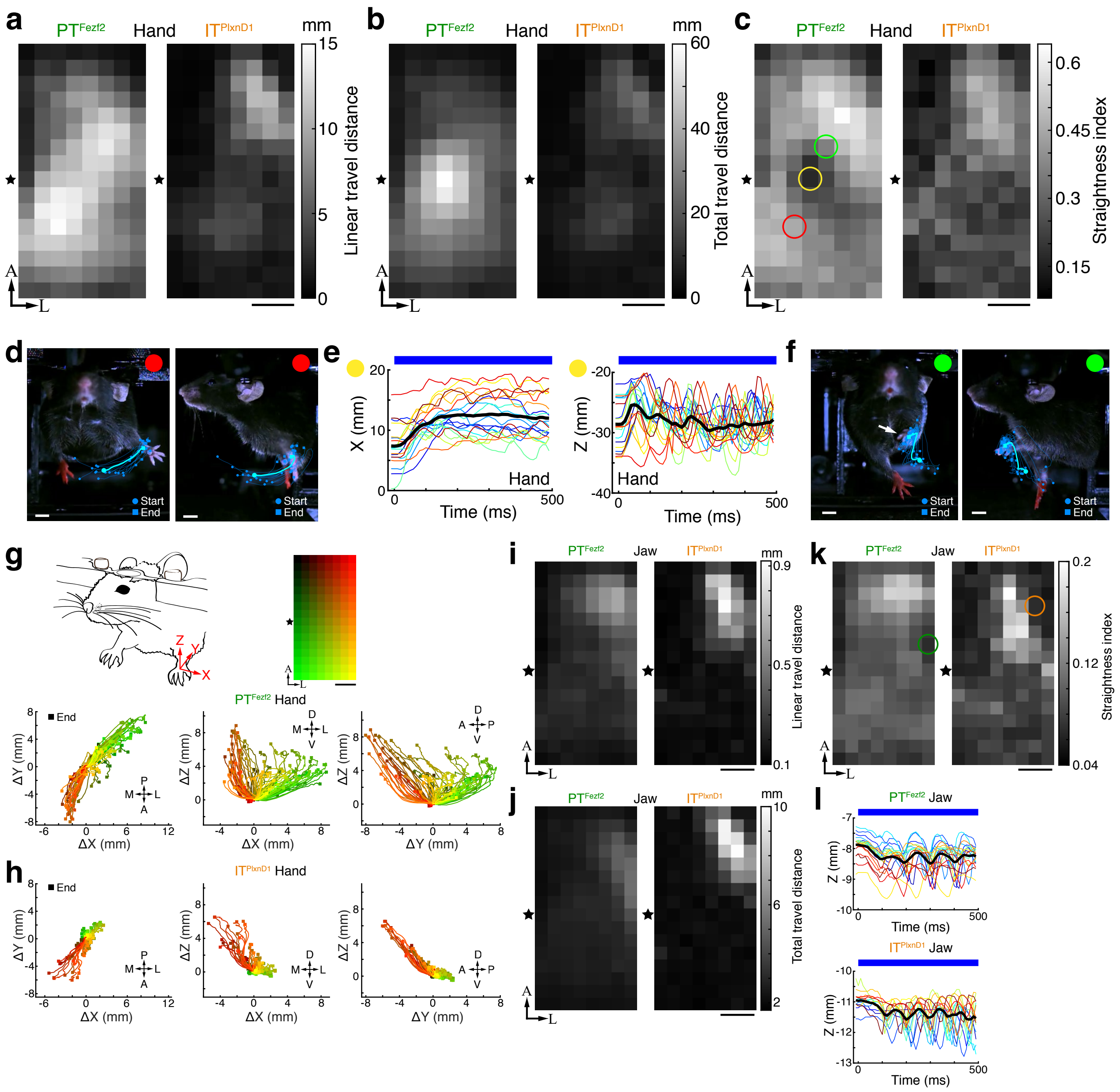

## Extended Data Figure 3

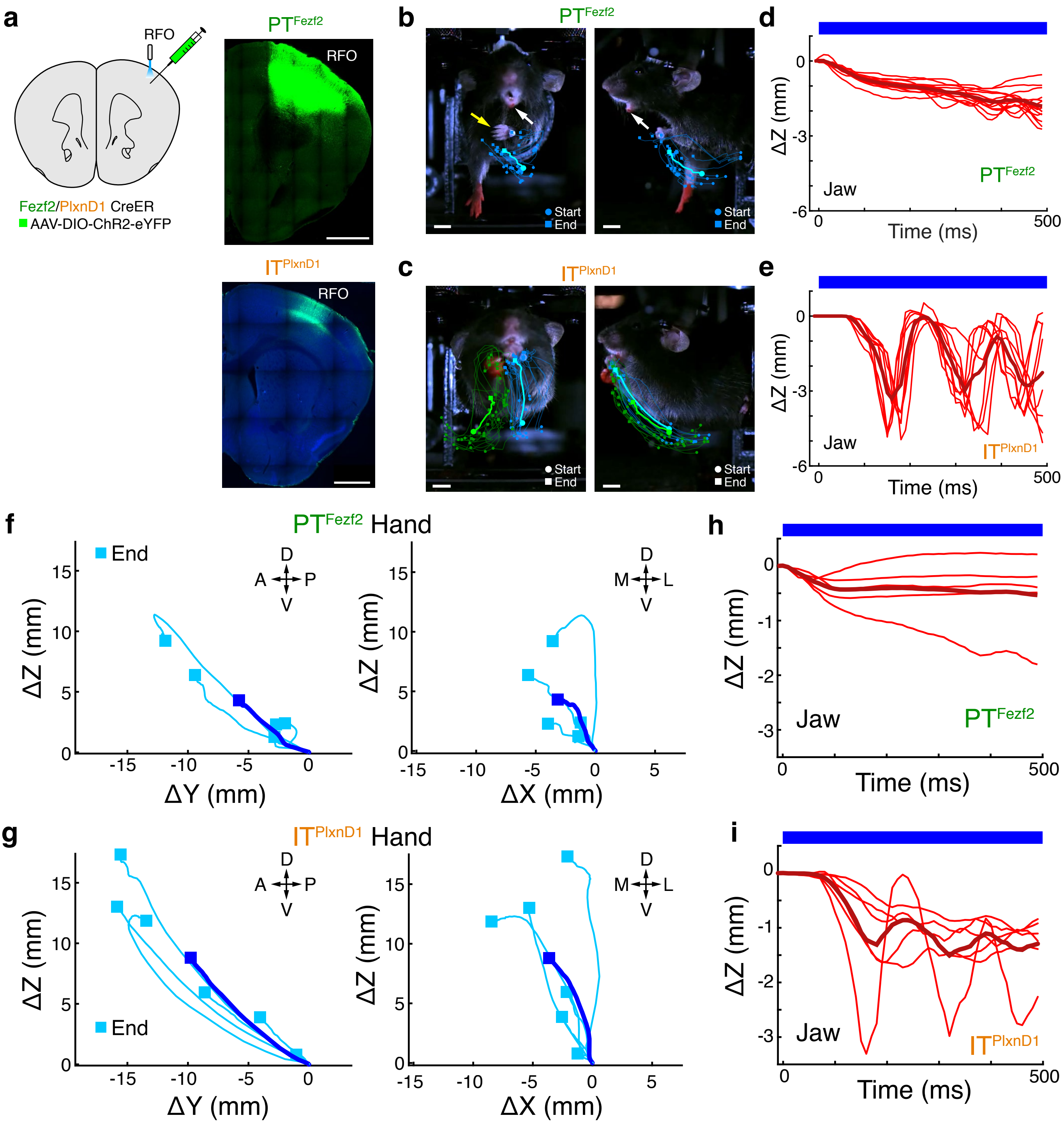

## Figure 2

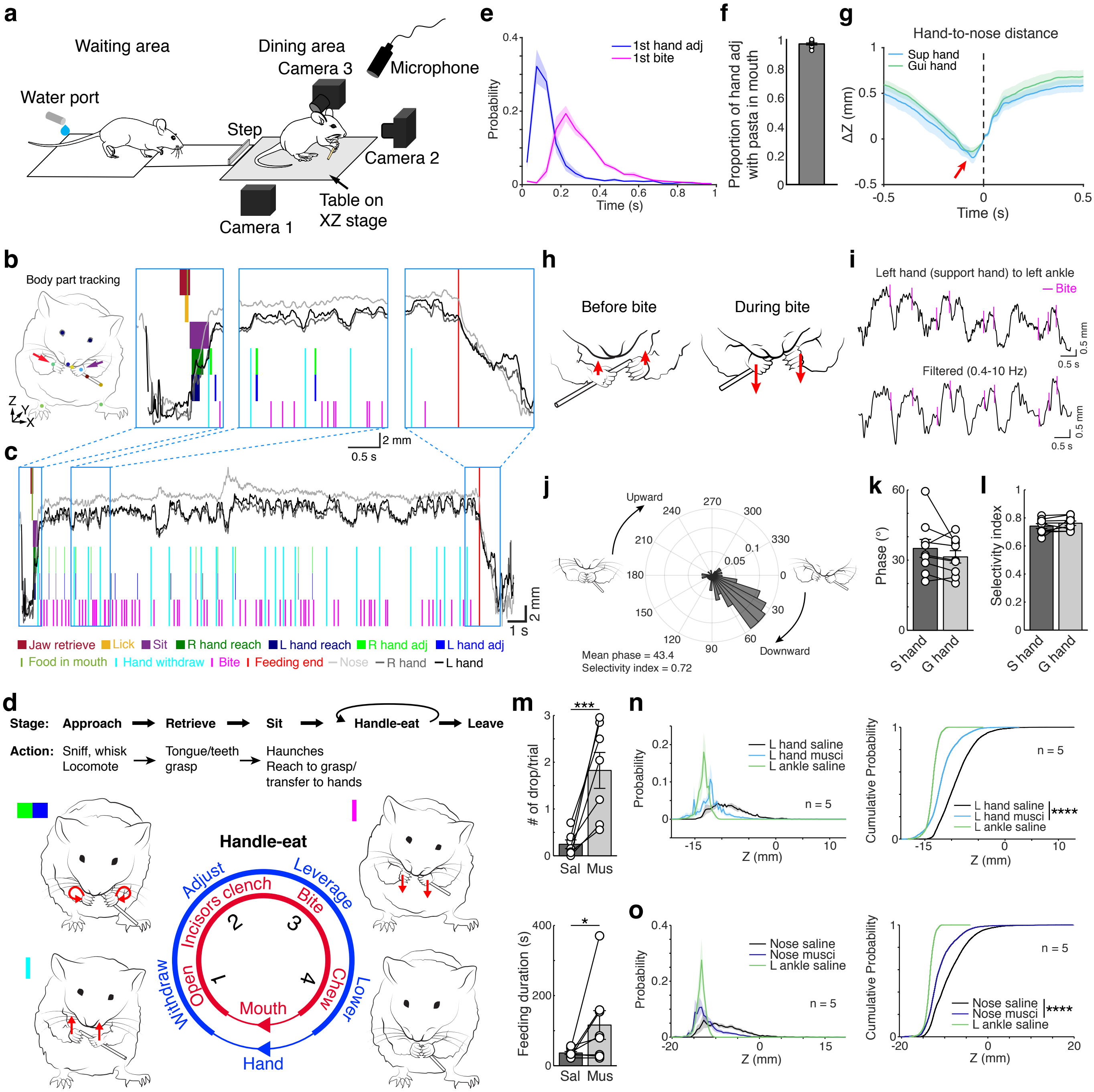

a
Waiting area
Dining area
Camera 3
Microphone
Water port
Step
Camera 2
Table on
XZ stage
Camera 1
e
1st hand adj
1st bite
Probability
Time (s)
f
Proportion of hand adj
with pasta in mouth
g
Hand-to-nose distance
Sup hand
Gui hand
ΔZ (mm)
Time (s)
b
Body part tracking
2 mm
0.5 s
c
1 s
2 mm
Jaw retrieve
Lick
Sit
R hand reach
L hand reach
R hand adj
L hand adj
Food in mouth
Hand withdraw
Bite
Feeding end
Nose
R hand
L hand
h
Before bite
During bite
i
Left hand (support hand) to left ankle
Bite
0.5 mm
0.5 s
Filtered (0.4-10 Hz)
0.5 mm
0.5 s
j
Upward
Downward
Mean phase = 43.4
Selectivity index = 0.72
k
Phase (°)
S hand
G hand
l
Selectivity index
S hand
G hand
d
Stage: Approach → Retrieve → Sit → Handle-eat → Leave
Action: Sniff, whisk Locomote → Tongue/teeth grasp → Haunches Reach to grasp/ transfer to hands
Handle-eat
Adjust
Leverage
Incisors clench
Bite
Withdraw
Open
Chew
Lower
Mouth
Hand
1
2
3
4
m
***
# of drop/trial
Sal
Mus
*
Feeding duration (s)
Sal
Mus
n
L hand saline
L hand musci
L ankle saline
Probability
n = 5
Z (mm)
Cumulative Probability
n = 5
L hand saline
L hand musci
L ankle saline
****
Z (mm)
o
Nose saline
Nose musci
L ankle saline
Probability
n = 5
Z (mm)
Cumulative Probability
n = 5
Nose saline
Nose musci
L ankle saline
****
Z (mm)

## Extended Data Figure 4

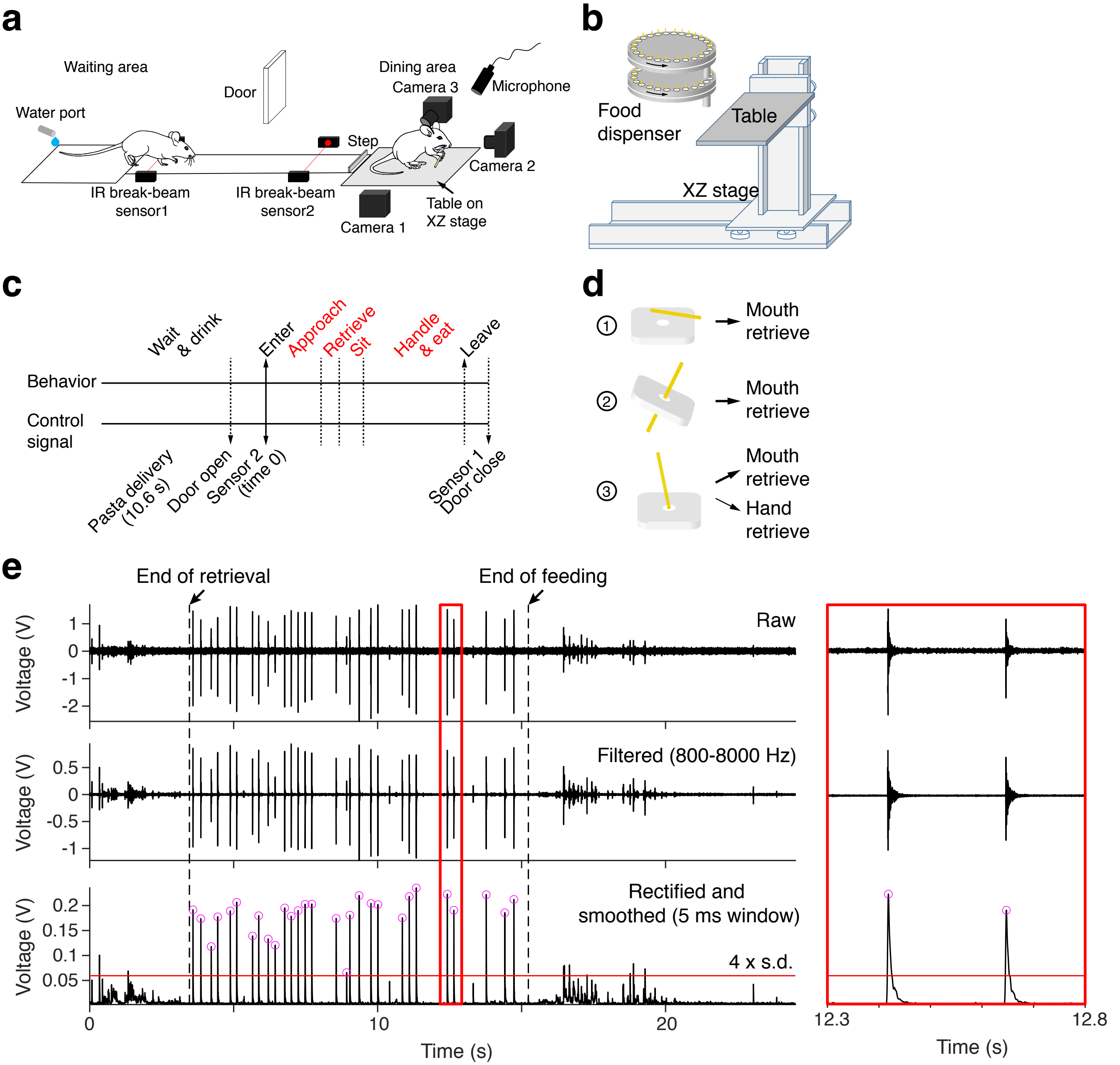

Extended Data Figure 5

Figure 2-2

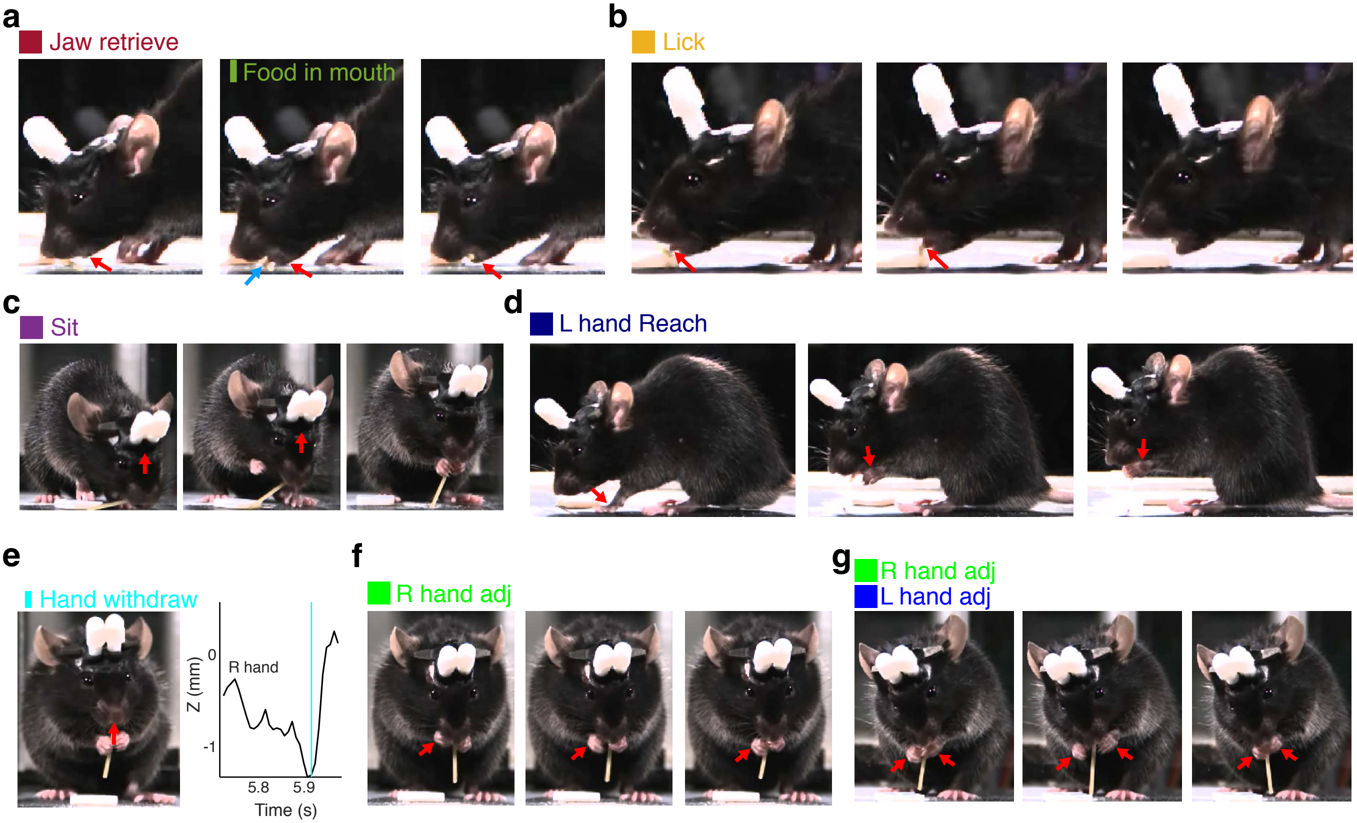

Extended Data Figure 6

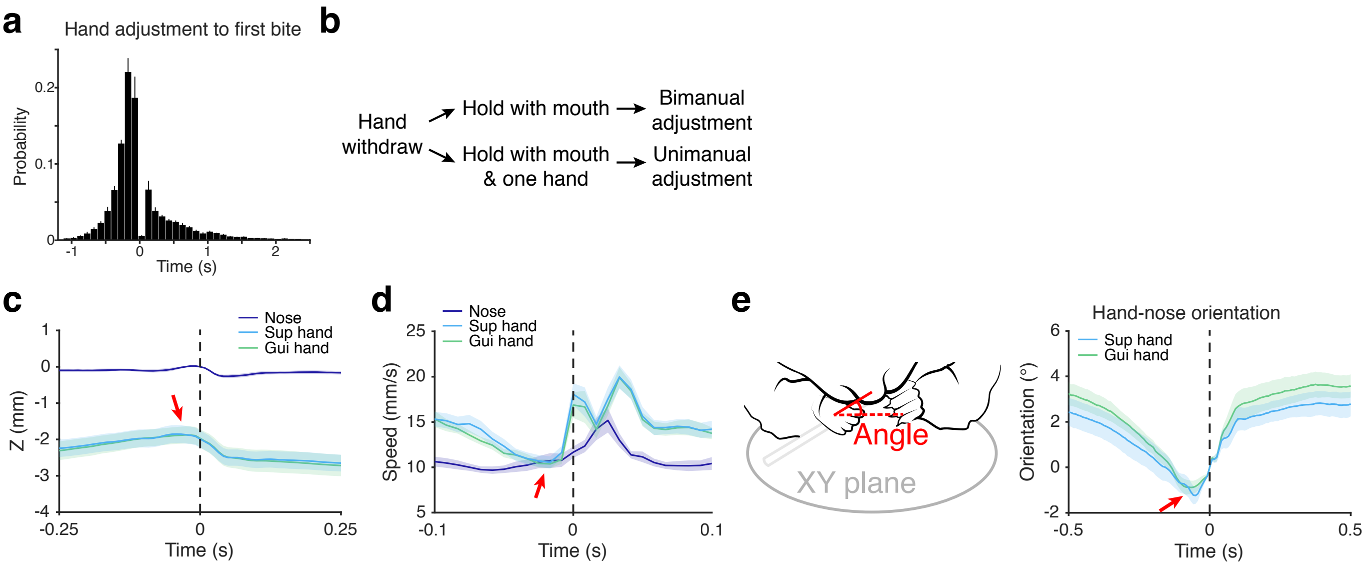

Extended Data Figure 7

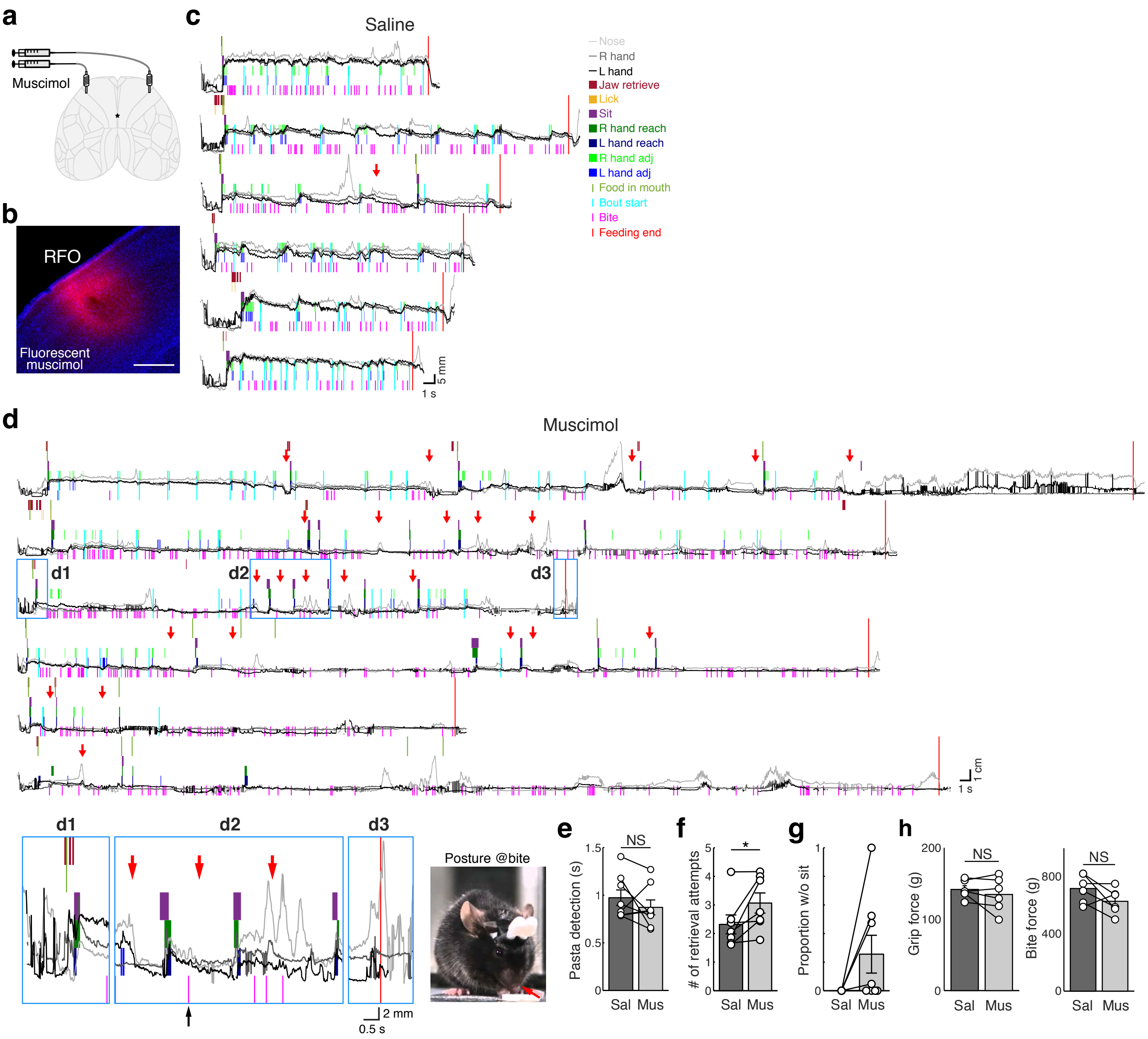

Figure 3

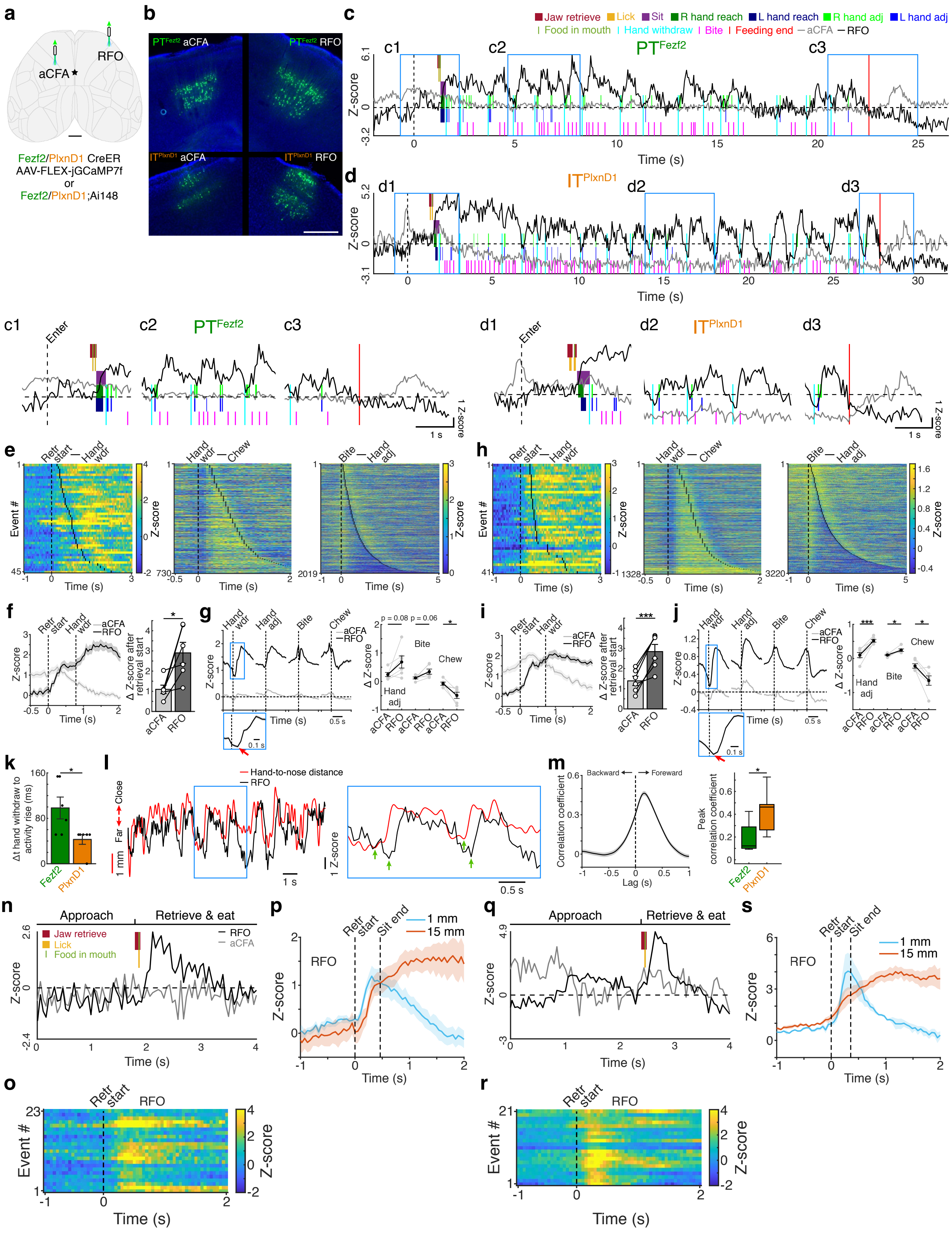

a
aCFA
RFO
Fezf2/PlxnD1 CreER
AAV-FLEX-jGCaMP7f
or
Fezf2/PlxnD1;Ai148
b
PTFezf2 aCFA
PTFezf2 RFO
ITPlxnD1 aCFA
ITPlxnD1 RFO
c
Jaw retrieve
Lick
Sit
R hand reach
L hand reach
R hand adj
L hand adj
Food in mouth
Hand withdraw
Bite
Feeding end
aCFA
RFO
PTFezf2
Z-score
Time (s)
d
ITPlxnD1
Enter
1 s
1 Z-score
e
Retr start
Hand wdr
Chew
Bite
Hand adj
Event #
Z-score
f
g
p = 0.08
p = 0.06
Δ Z-score after retrieval start
Δ Z-score
h
i
j
k
Δt hand withdraw to activity rise (ms)
Fezf2
PlxnD1
l
Hand-to-nose distance
Close
Far
1 mm
m
Backward
Forward
Correlation coefficient
Lag (s)
Peak correlation coefficient
n
Approach
Retrieve & eat
p
Sit end
1 mm
15 mm
o
q
s
r

## Extended Data Figure 8

Figure 3-1

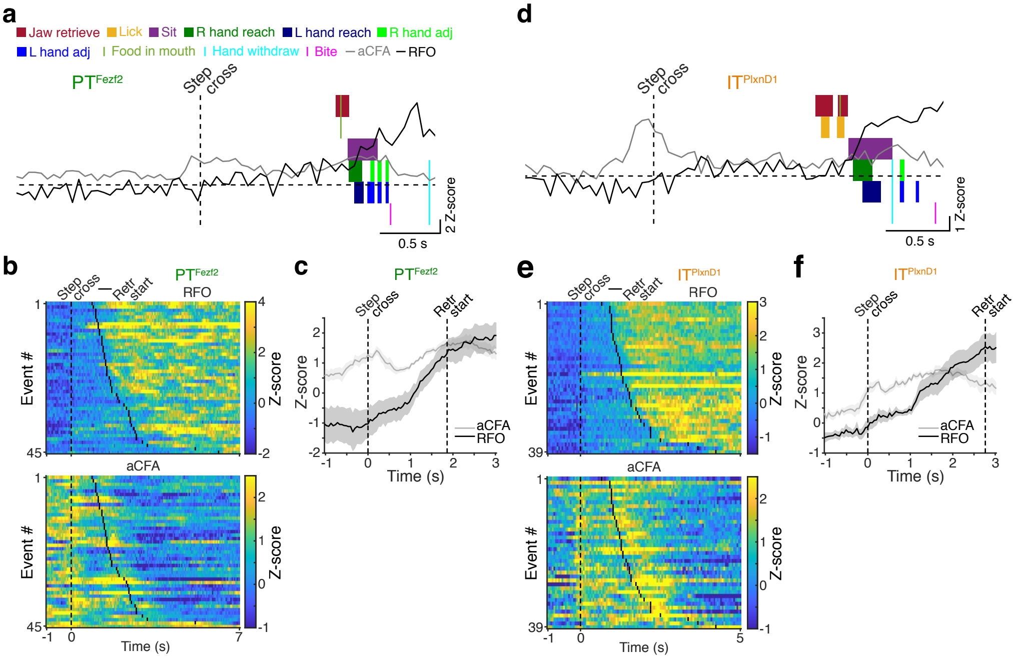

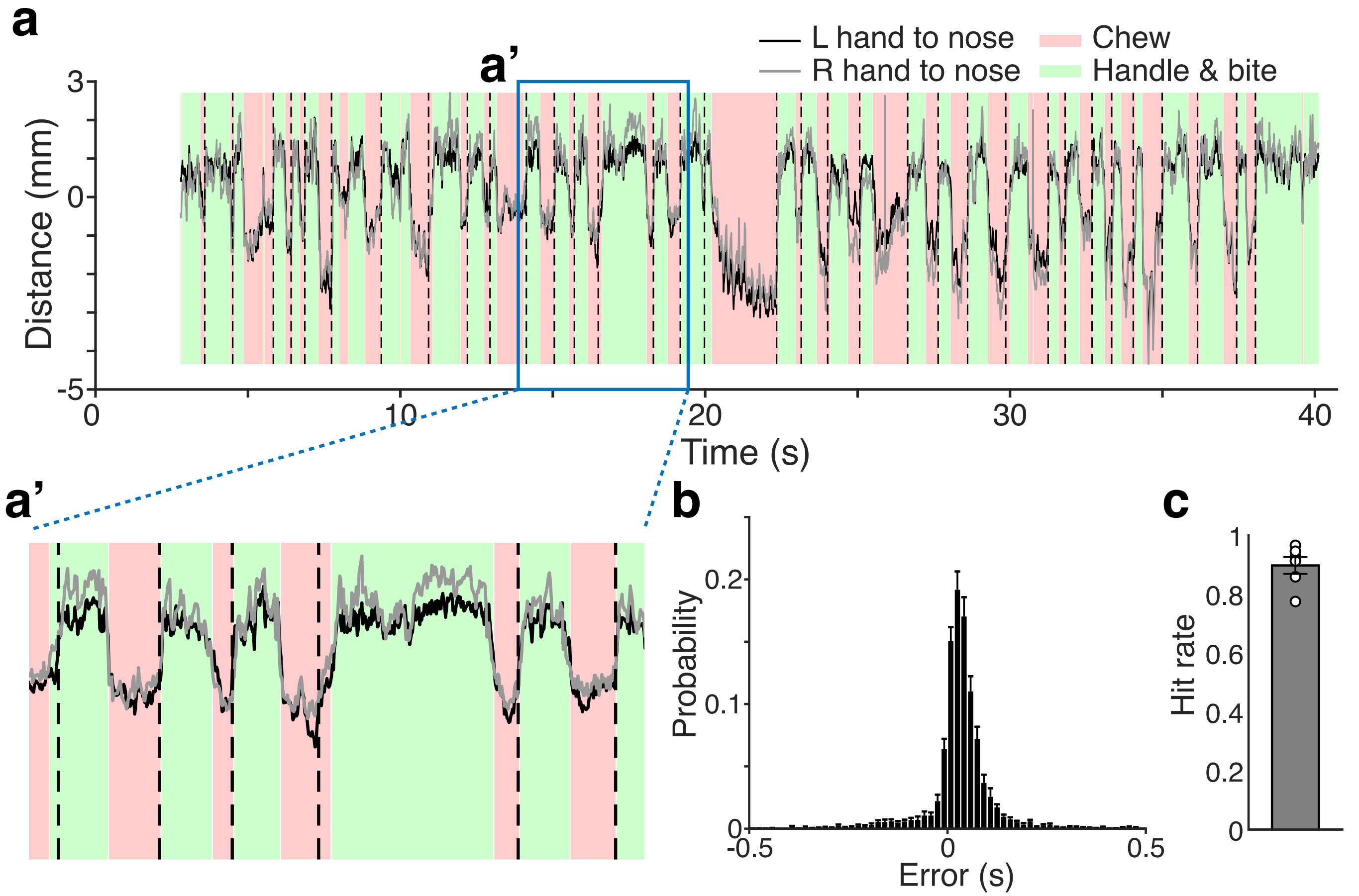

a
L hand to nose
R hand to nose
Chew
Handle & bite
a’
3
0
-5
Distance (mm)
0
10
20
30
40
Time (s)
a’
b
0.2
0.1
0
Probability
-0.5
0
0.5
Error (s)
c
1
0.8
0.6
0.4
0.2
0
Hit rate

Figure 4

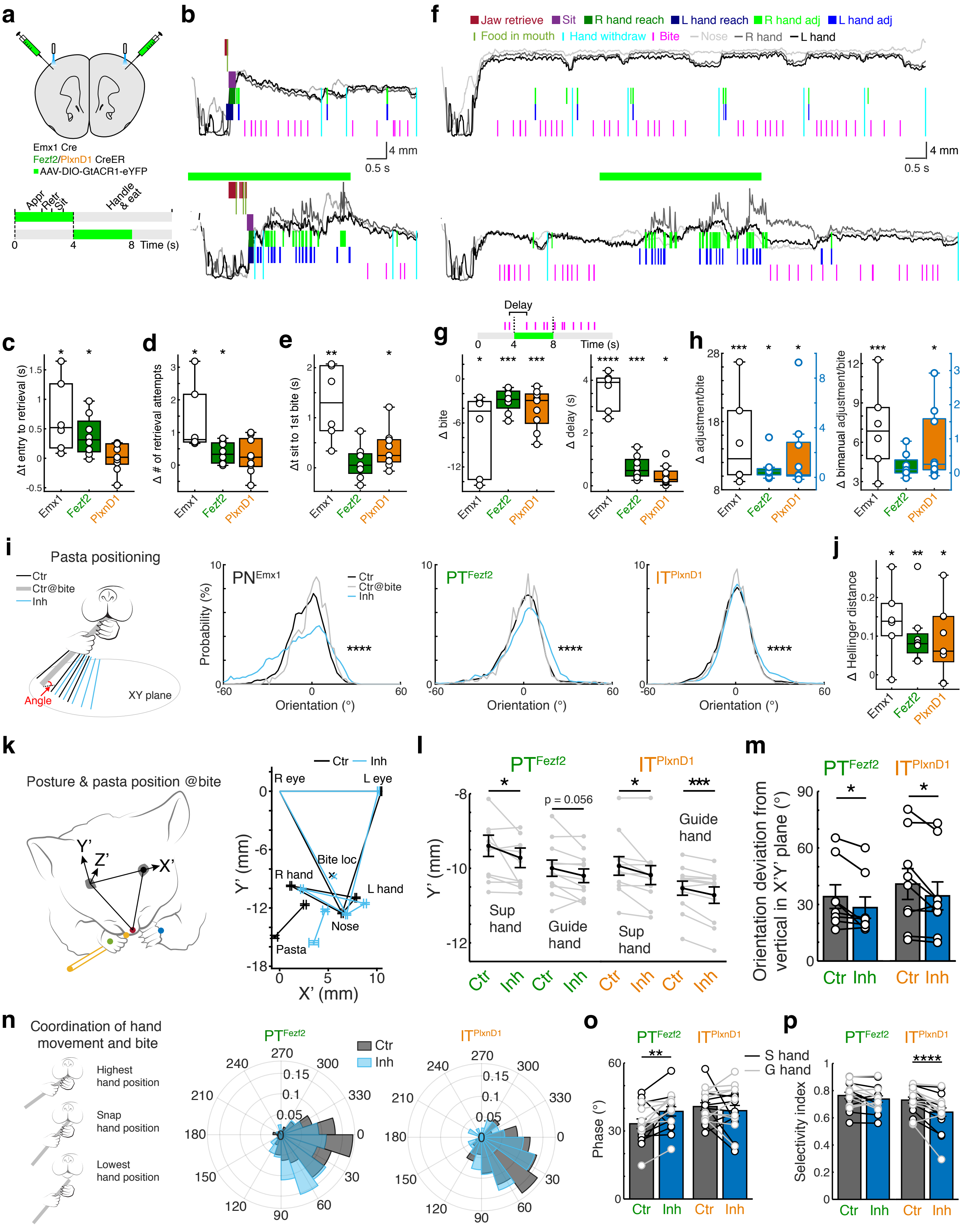

a
Emx1 Cre
Fezf2/PlxnD1 CreER
AAV-DIO-GtACR1-eYFP
Appr
Retr
Sit
Handle & eat
Time (s)
b
4 mm
0.5 s
f
Jaw retrieve
Sit
R hand reach
L hand reach
R hand adj
L hand adj
Food in mouth
Hand withdraw
Bite
Nose
R hand
L hand
c
Δt entry to retrieval (s)
d
Δ # of retrieval attempts
e
Δt sit to 1st bite (s)
g
Delay
Time (s)
Δ bite
Δ delay (s)
h
Δ adjustment/bite
Δ bimanual adjustment/bite
Emx1
Fezf2
PlxnD1
i
Pasta positioning
Ctr
Ctr@bite
Inh
Angle
XY plane
Probability (%)
Orientation (°)
j
Δ Hellinger distance
k
Posture & pasta position @bite
Y'
Z'
X'
R eye
L eye
Bite loc
R hand
L hand
Nose
Pasta
Y' (mm)
X' (mm)
l
Sup hand
Guide hand
p = 0.056
m
Orientation deviation from vertical in X'Y' plane (°)
n
Coordination of hand movement and bite
Highest hand position
Snap hand position
Lowest hand position
o
S hand
G hand
Phase (°)
p
Selectivity index

Extended Data Figure 10

Figure 4-1

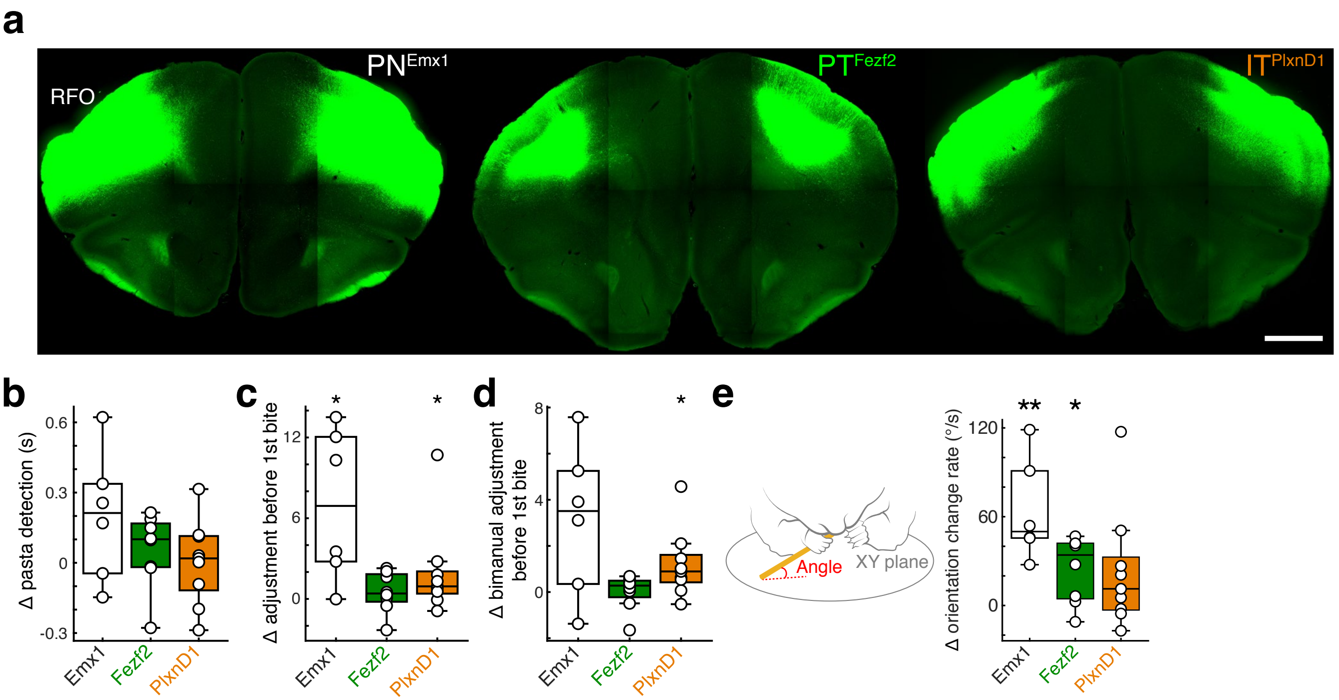

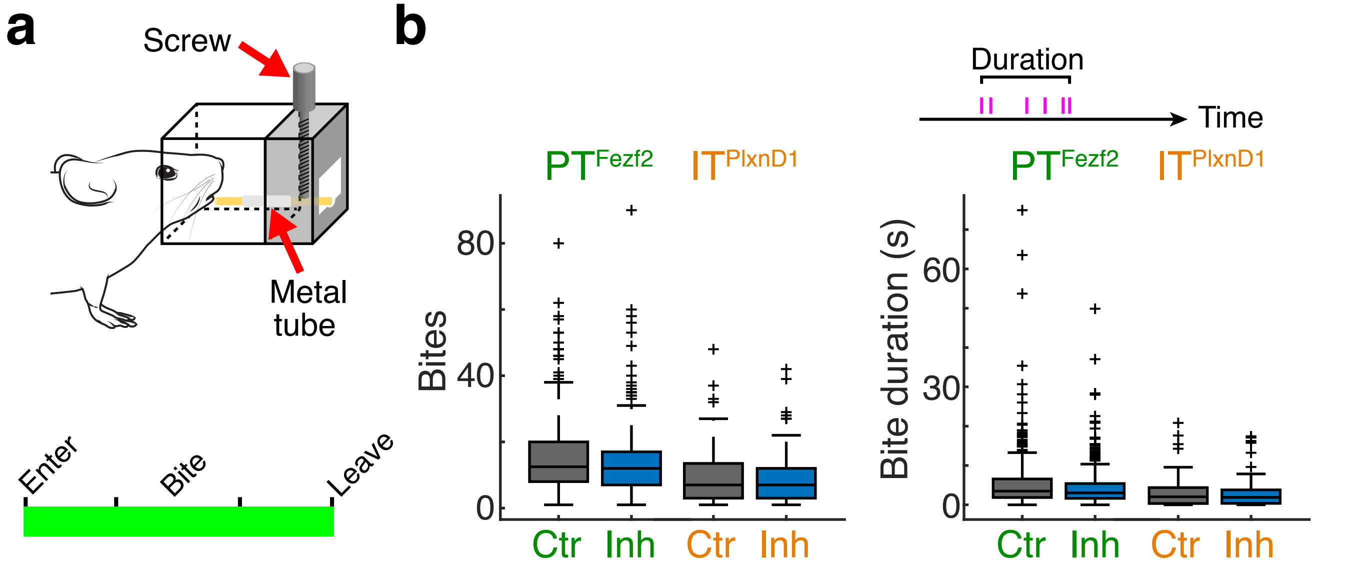

a
Screw
Metal
tube
Enter
Bite
Leave
b
Duration
Time
PTFezf2
ITPlxnD1
Bites
80
40
0
Ctr
Inh
Ctr
Inh
PTFezf2
ITPlxnD1
Bite duration (s)
60
30
0
Ctr
Inh
Ctr
Inh

## Extended Data Figure 12

Figure 4-3

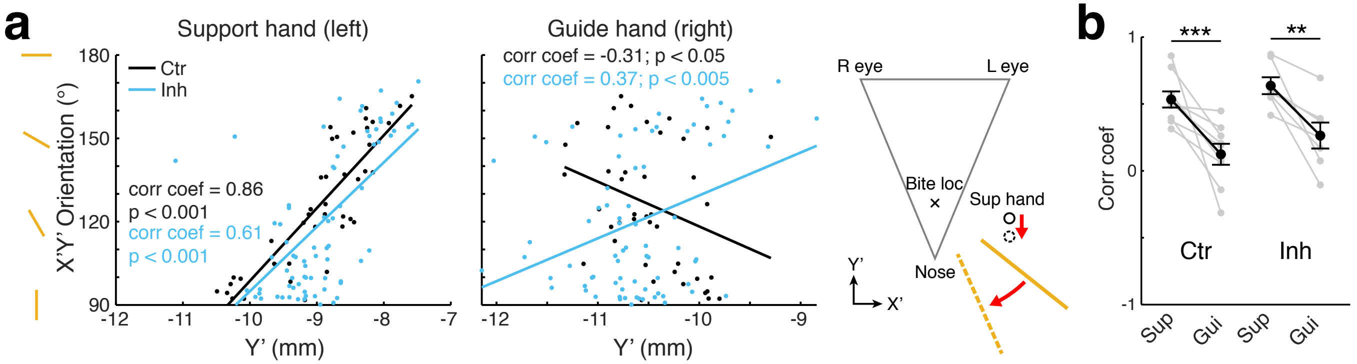

## Extended Data Figure 13

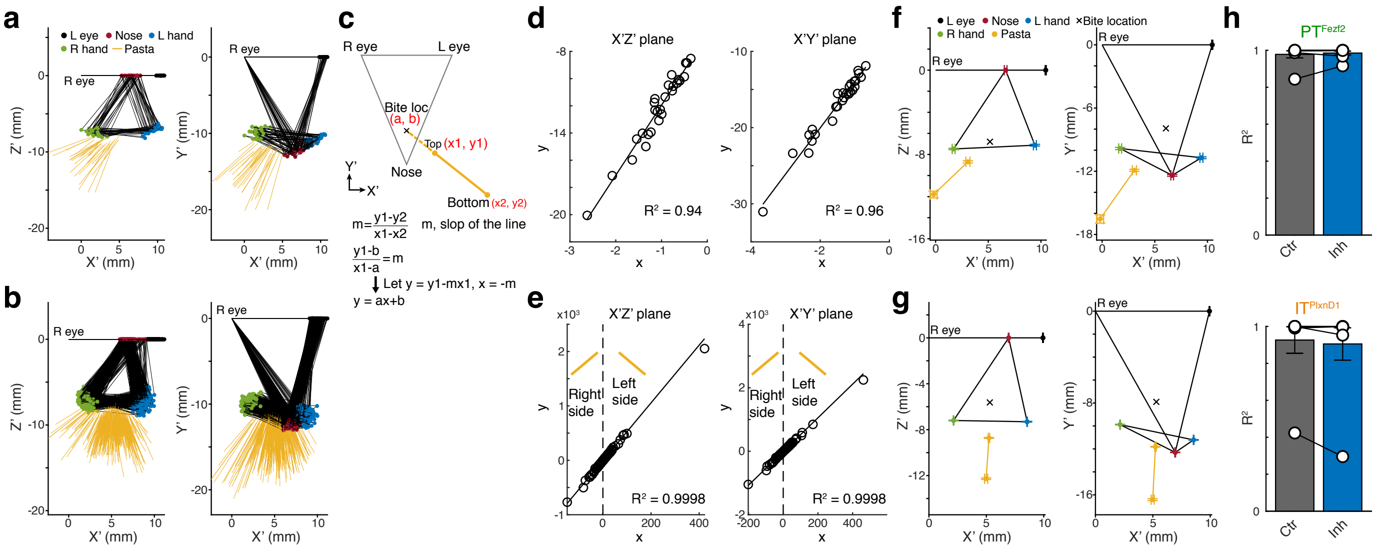

## Figure 5

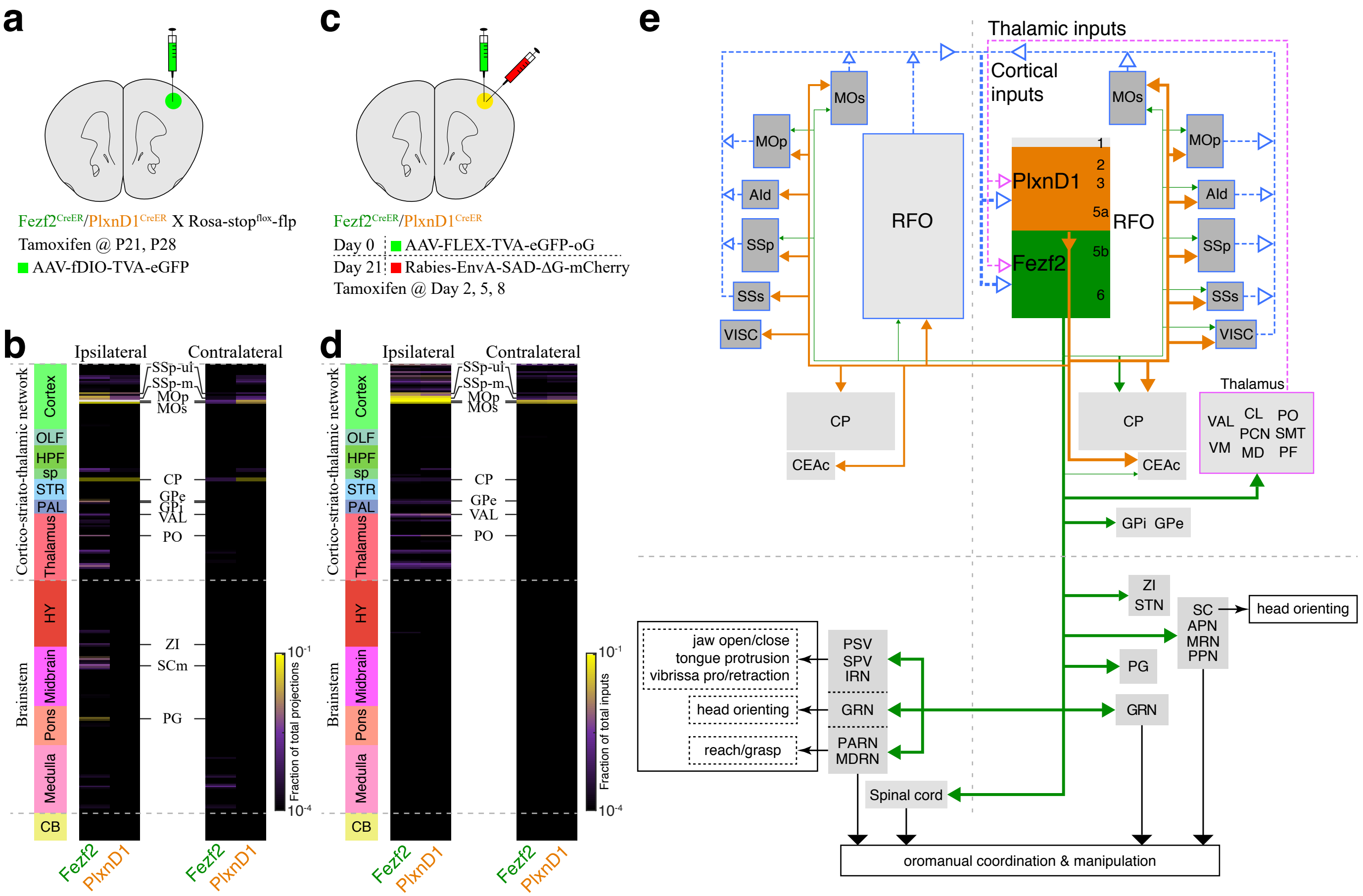

a
Fezf2CreER/PlxnD1CreER X Rosa-stopflox-flp
Tamoxifen @ P21, P28
AAV-fDIO-TVA-eGFP
c
Fezf2CreER/PlxnD1CreER
Day 0 AAV-FLEX-TVA-eGFP-oG
Day 21 Rabies-EnvA-SAD-ΔG-mCherry
Tamoxifen @ Day 2, 5, 8
b
Ipsilateral
Contralateral
Cortico-striato-thalamic network
Brainstem
Cortex
OLF
HPF
sp
STR
PAL
Thalamus
HY
Midbrain
Pons
Medulla
CB
SSp-ul
SSp-m
MOp
MOs
CP
GPe
GPi
VAL
PO
ZI
SCm
PG
Fraction of total projections
Fezf2
PlxnD1
d
Fraction of total inputs
e
Thalamic inputs
Cortical inputs
MOs
MOp
AId
SSp
SSs
VISC
RFO
PlxnD1
Fezf2
1
2
3
5a
5b
6
CP
CEAc
Thalamus
VAL
CL
PO
PCN
SMT
VM
MD
PF
GPi GPe
ZI
STN
SC
APN
MRN
PPN
head orienting
PG
GRN
PSV
SPV
IRN
PARN
MDRN
jaw open/close
tongue protrusion
vibrissa pro/retraction
reach/grasp
Spinal cord
oromanual coordination & manipulation

## Extended Data Figure 14
